# Heterobimetallic Gold/Ruthenium Complexes Synthesized via Post‐functionalization and Applied in Dual Photoredox Gold Catalysis

**DOI:** 10.1002/chem.202201856

**Published:** 2022-09-01

**Authors:** Lea Bayer, Bernhard S. Birenheide, Felix Krämer, Sergei Lebedkin, Frank Breher

**Affiliations:** ^1^ Karlsruhe Institute of Technology (KIT) Institute of Inorganic Chemistry, Division Molecular Chemistry Engesserstraße 15 76131 Karlsruhe Germany; ^2^ Karlsruhe Institute of Technology (KIT) Institute of Nanotechnology Postfach 3630 76021 Karlsruhe Germany

**Keywords:** bimetallic complexes, cooperative effects, metalloligands, photoredox catalysts, post-functionalization

## Abstract

The synthesis of heterobimetallic Au^I^/Ru^II^ complexes of the general formula *syn*‐ and *anti*‐[{AuCl}(**L1**∩**L2**){Ru(bpy)_2_}][PF_6_]_2_ is reported. The ditopic bridging ligand **L1**∩**L2** refers to a P,N hybrid ligand composed of phosphine and bipyridine substructures, which was obtained via a post‐functionalization strategy based on Diels‐Alder reaction between a phosphole and a maleimide moiety. It was found that the stereochemistry at the phosphorus atom of the resulting 7‐phosphanorbornene backbone can be controlled by executing the metal coordination and the cycloaddition reaction in a different order. All precursors, as well as the mono‐ and multimetallic complexes, were isolated and fully characterized by various spectroscopic methods such as NMR, IR, and UV‐vis spectroscopy as well as cyclic voltammetry. Photophysical measurements show efficient phosphorescence for the investigated monometallic complex *anti*‐[(**L1**∩**L2**){Ru(bpy)_2_}][PF_6_]_2_ and the bimetallic analogue *syn*‐[{AuCl}(**L1**∩**L2**){Ru(bpy)_2_}][PF_6_]_2_, thus indicating a small influence of the {AuCl} fragment on the photoluminescence properties. The heterobimetallic Au^I^/Ru^II^ complexes *syn*‐ and *anti*‐[{AuCl}(**L1**∩**L2**){Ru(bpy)_2_}][PF_6_]_2_ are both active catalysts in the *P*‐arylation of aryldiazonium salts promoted by visible light with *H*‐phosphonate affording arylphosphonates in yields of up to 91 %. Both dinuclear complexes outperform their monometallic counterparts.

## Graphical abstract

**Acting in concert**: Novel heterobimetallic Au^I^/Ru^II^ complexes synthesized in a Diels‐Alder reaction are reported. The applied post‐functionalization strategy allows which isomer is obtained to be controlled. Their application as catalysts in carbon‐phosphorus cross‐coupling showed that the dinuclear complexes can outperform their monometallic counterparts

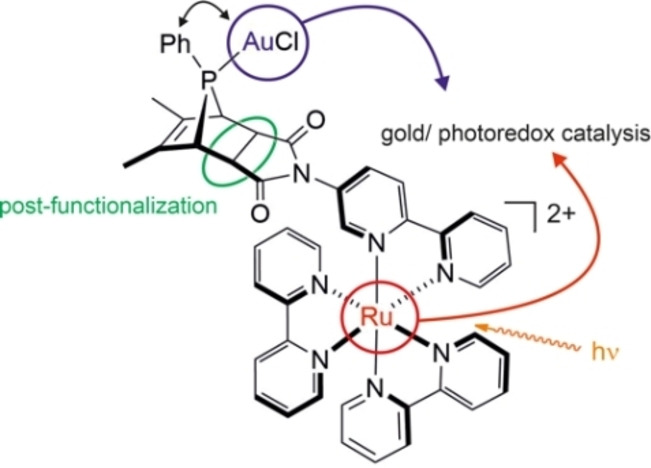

## Introduction

The development of multimetallic complexes has garnered significant interest from the scientific community in recent years.[^1^, ^2^] While the underlying principles in monometallic systems are relatively well explored, complexes containing more than one metal atom often show unexpected properties. Alongside unique optical^[3]^ or magnetic^[4]^ properties, for instance, cooperativity between the metal centers can be exploited for catalytic applications.^[5]^ Multimetallic approaches can enable transformations that are unfeasible with classic protocols as the activity of one metal can be enhanced by the other and new reaction pathways may become accessible through cooperative effects.^[6]^ It has been shown that heterobimetallic complexes can exhibit increased reactivity in catalysis as compared to 1 : 1 mixtures of related monometallic complexes or the analogous homobimetallic counterparts.[^2^, ^7^]

With that in mind, and based on our interest in studying heterobimetallic complexes in homogeneous catalysis,^[8]^ we became interested in studying heterobimetallic complexes in dual photoredox gold catalysis.^[9]^ During the past two decades, much research has been devoted to promote the field of homogeneous gold catalysis beyond π activation of C−C multiple bonds.[^10^, ^11^] One of the biggest challenges approaching gold redox chemistry was to overcome the redox stability of gold(I).^[12]^ The requirement for external oxidizing reagents made this research field less attractive, in particular for the total synthesis of complex molecules bearing a variety of functional groups. Glorius et al. and Toste et al. independently reported on merging gold catalysis with photoredox catalysis.[^13^, ^14^] The use of low‐energy visible light, to generate radical species that facilitate a stepwise oxidation of Au^I^, made Au^I^/Au^III^ coupling reactions accessible without the need for stoichiometric amounts of strong oxidants. As it is the case for most reported dual catalytic systems, the individual catalysts are added as independent species to the reaction mixture, that is, the reactions are typically performed using a simple gold(I) phosphine complex, combined with a [Ru(bpy)_3_]^2+^ system as photocatalyst.[^13^, ^14^, ^15^, ^16^]

Related to Au/Ru architectures, the employment of bimetallic, single compound catalysts that feature two distinct catalytic sites, has not gained much attention so far and only a few examples have been reported to date.[^17^, ^18^] A possible explanation might be that the preparation of well‐defined heterobimetallic complexes still remains a challenge. Commonly, P‐ or N‐based hybrid ligands^[19]^ like phosphines or pyridines are lacking simple protection‐deprotection protocols that allow successive and selective ligation of various metal centers.^[20]^ Thus, we intended to develop a strategy that enables the combination of mononuclear building blocks through post‐functionalization schemes – a modern synthetic approach to “tailor‐made” ligand scaffolds.^[21]^ For instance, Veige and co‐workers were able to synthesize homo‐ and heterodinuclear transition metal complexes by applying 1,3‐dipolar cycloaddition reactions.^[22]^

**Figure 1:**
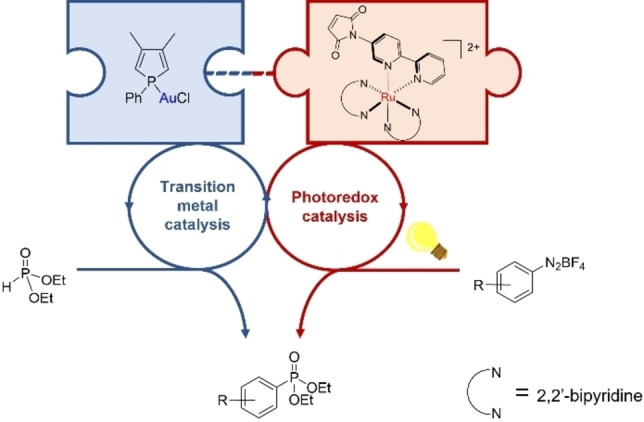
Tethering transition metal catalyst and photoredox catalyst by post‐functionalization.

In the realm of cycloaddition reactions, the Diels‐Alder reaction could also be valuable as its diversity opens up a multitude of different structural motifs.^[23]^ Accordingly, we chose a 1,2‐disubstituted phosphole, namely 3,4‐dimethyl‐1‐phenyl phosphole (dmpp, **L1**), to fulfill the function of being a P‐based ligand with good σ‐donor abilities along with moderate steric hinderance,^[24]^ and at the same time a suitable diene showing only small amount of aromatic character.^[25]^ As it was already shown that dmpp readily undergoes Diels‐Alder reaction with *N*‐phenyl maleimide, we further supposed that replacing the phenyl substituent at the maleimide nitrogen atom with a 2,2’‐bipyridine (bpy) moiety could provide a suitable dienophile simultaneously serving as N‐donor ligand (Figure 1).^[26]^

To assess the catalytic performance of the heterobimetallic complexes, we have chosen a carbon‐phosphorus cross‐coupling reaction mediated by gold and photoredox catalysis. The resulting aryl phosphonates have attracted increasing attention as they are structural motifs found in many pharmaceutically active molecules.^[27]^ Moreover, they find broad application as synthetic intermediates, agrochemicals and in material science.^[28]^

## Results and Discussion

**Scheme 1:**
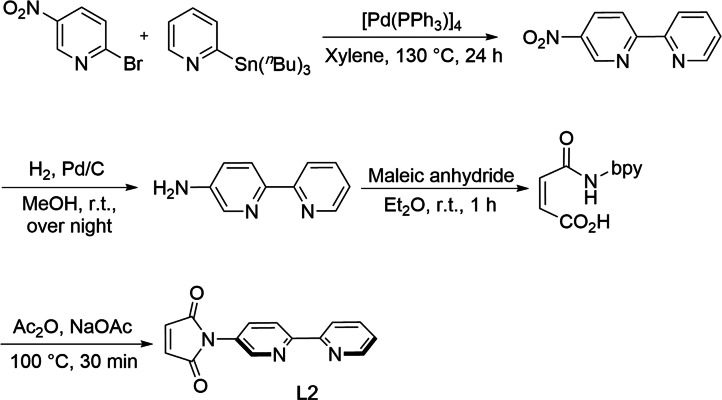
Synthesis of **L2**. bpy=4‐(2,2‘‐bipyridyl).

The diene and P‐donor ligand dmpp (**L1**),^[29]^ as well as its gold(I) complex [(dmpp)AuCl] ([(**L1**){AuCl}], **1**),^[24]^ were prepared according to literature procedures. The synthesis of the desired 2,2’‐bipyridyl‐substituted maleimide, which should accomplish the task of both being a bidentate N‐donor ligand and an electron‐poor dienophile appropriate for Diels‐Alder reaction, was found to be straightforward. The short sequence starts with the synthesis of 5‐nitro‐2,2‘‐bipyridine^[30]^ by Stille‐coupling of 2‐bromo‐5‐nitropyridine with 2‐(tributylstannyl) pyridin, followed by reduction to furnish the corresponding 5‐amino‐2,2‘‐bipyridine.[^30^, ^31^] Treatment of the latter with maleic anhydride subsequently gave the *N*‐bipyridyl maleamic acid, which was converted to *N*‐bipyridyl maleimide (**L2**) as yellow solid in 66 % yield (Scheme 1).

**Scheme 2:**
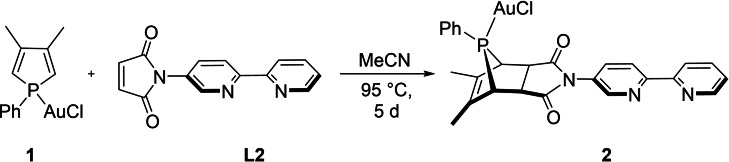
Synthesis of *syn*‐[{AuCl}(**L1**∩**L2**)] (**2**).

In order to provide a metalloligand scaffold suitable to coordinate two metals in a pre‐defined orientation, **L2** and [(**L1**){AuCl}] (**1**) were employed in a Diels‐Alder reaction (Scheme 2).

The resulting “click” product *syn*‐[{AuCl}(**L2**∩**L1**)] (**2**) precipitated directly from the reaction mixture and was isolated as colorless solid in 64 % yield. The ^31^P{^1^H} NMR chemical shift of *δ* ${}^{{}^{31}P}$ =110.4 ppm is strongly downfield‐shifted as compared to **1** (cf. *δ* ${}^{{}^{31}P}$ =25.9 ppm). The deshielding of the P nucleus is typical for trivalent 7‐phosphanorbornenes and particularly distinctive for the *syn* isomers (i. e., those possessing the P−R functionality *syn* to the C=C double bond).^[32]^ Moreover, the C(O)−*C*H sp^3^ carbon atoms exhibit large coupling constants of ^2^ *J*_C‐P_=24.4 Hz to ^31^P, in contrast to the sp^2^ carbon atoms. All these features are characteristic of the *syn* stereochemistry at the P atom.^[33]^

Slow diffusion of *n*‐hexane into a methylene chloride solution of **2** leads to the formation of single crystals as fine needles. Due to the small size of the crystals, X‐ray data were always of poor quality. Nonetheless, the structural motif is identifiable as the *syn‐endo* isomer and thus supports the observations made by NMR spectroscopy (see Section S1 of the Supporting Information).

**Scheme 3:**
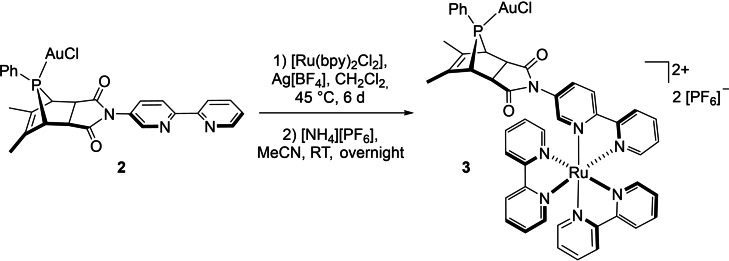
Synthesis of *syn*‐[{AuCl}(**L1**∩**L2**){Ru(bpy)_2_}][PF_6_]_2_ (**3**).

The final step of developing the heterobimetallic version of the photoredox catalyst system was to incorporate the [Ru(bpy)_3_]^2+^ moiety as photocatalytic entity. Reaction of *syn*‐[{AuCl}(**L1**∩**L2**)] (**2**) with [Ru(bpy)_2_Cl_2_] in the presence of Ag[BF_4_], and subsequent treatment with [NH_4_][PF_6_], led to the formation of the targeted heterodinuclear complex *syn*‐[{AuCl}(**L1**∩**L2**){Ru(bpy)_2_}][PF_6_]_2_ (**3**) as orange crystals in 73 % isolated yield (Scheme 3).

As expected, the coordination of the {Ru(bpy)_2_} fragment causes no significant change in the ^31^P{^1^H} NMR spectrum (*δ* ${}^{{}^{31}P}$ =111.4 ppm). Overall, the NMR spectroscopic data compare well with those of **2**. While the C(O)−*C*H sp^3^ carbon atoms show a strong coupling to the phosphorus (^2^ *J*_C‐P_=24.3 Hz), no coupling is detected for the sp^2^ carbon atoms (see also below).

**Figure 2:**
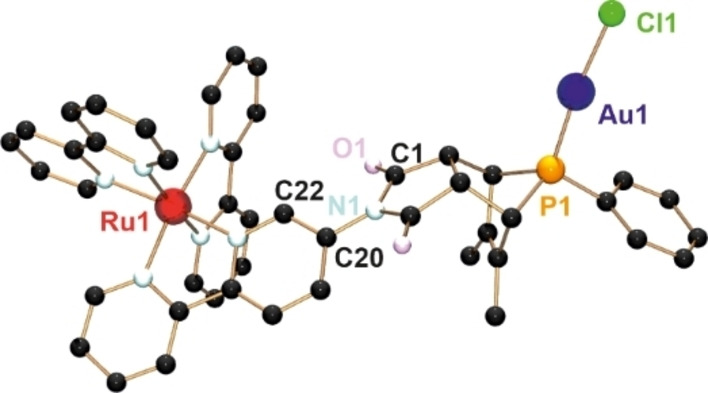
Molecular structure of *syn*‐[{AuCl}(**L1**∩**L2**){Ru(bpy)_2_}][PF_6_]_2_ (**3**). Hydrogen atoms, solvent molecules and counter ions have been omitted for clarity. Selected bond lengths [pm], angles [°] and distances [pm]: P1−Au1 221.53(18), Au1−Cl1 228.07(18), øRu1−N(bpy) 205.7(5); P1−Au1−Cl1 173.94(7), C1−N1−C20−C22 35.94; Au1⋅⋅⋅Ru1 1082.6 pm.

Crystals suitable for X‐ray diffraction were obtained by slow diffusion of an acetonitrile solution into benzene. The heterobimetallic complex **3** crystalizes in the monoclinic space group *P*2_1_/*c* with 1.5 molecules of benzene in the asymmetric unit (Figure 2).

The molecular structure confirms the formation of the *syn‐endo* product. The P1−Au1 and Au1−Cl1 bond lengths of 221.5 and 228.1 pm, respectively, are in the expected range,^[24]^ whereas the P1−Au1−Cl1 angle of 173.9° is slightly distorted from the typical linear coordination mode of gold(I), with the chlorine atom bent towards the phenyl ring. The bipyridine moiety of the bridging ligand **L1**∩**L2** is twisted out of maleimide plane by a torsion angle of 35.9° (C1−N1−C20−C22). The distances between the nitrogen atoms and the central ruthenium atom range from 205.0 to 206.8 pm, resulting in an average Ru1−N bond length of 205.7 pm, which compares well to literature values.^[34]^ The distance between the two metal centers *d*(Au1⋅⋅⋅Ru1) is 1082.6 pm.

**Scheme 4:**
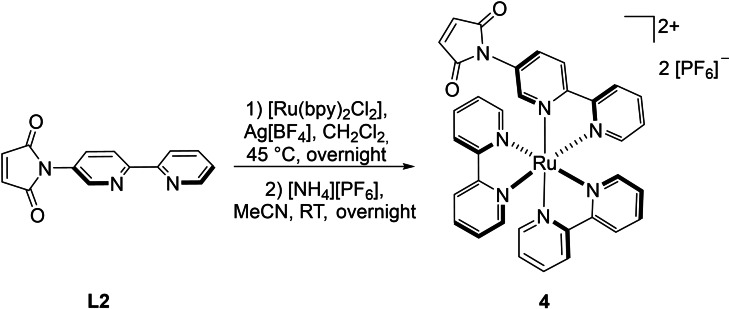
Synthesis of [(**L2**){Ru(bpy)_2_}][PF_6_]_2_ (**4**).

We additionally prepared the ruthenium complex of **L2**, that is, [(**L2**){Ru(bpy)_2_}][PF_6_]_2_ (**4**; Scheme 4) in order to check whether **3** can directly be obtained from the Diels‐Alder reaction of both mononuclear building blocks **1** and **4**.

**Figure 3:**
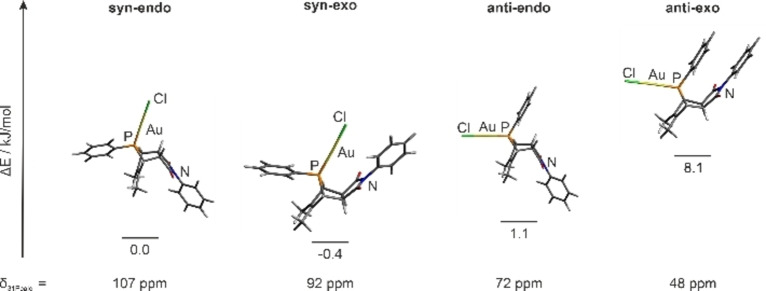
Calculated (BP86/SVP/D3BJ) minimum‐energy structures of the *syn*‐*endo*, *syn*‐*exo*, *anti*‐*endo* and *anti*‐*exo* isomers, their relative energies towards the *syn*‐*endo* isomer, and the calculated (TPSS/TZVP) ^31^P NMR chemical shifts.

[(**L1**){AuCl}] (**1**) and [(**L2**){Ru(bpy)_2_}][PF_6_]_2_ (**4**) were placed in a Schlenk flask and heated to 115 °C in *ortho*‐dichlorobenzene for 8 days and, due to better solubility of the substrates, in MeCN for another 3 days at 95 °C. Indeed, the heterodinuclear complex *syn*‐[{AuCl}(**L1**∩**L2**){Ru(bpy)_2_}][PF_6_]_2_ (**3**) as shown in Figure 2 is the main product of this reaction. However, the conversion takes place much slower as compared to the reaction of **L2** with **1**. Higher temperatures, as an attempt to increase the reaction rate, were not applied since phospholes are known to undergo a sigmatropic rearrangement under these conditions.[^23^, ^35^] A second ^31^P NMR signal at *δ* ${}^{{}^{31}P}$ =77.7 ppm present in the ^31^P{^1^H} NMR spectrum suggested the formation of a by‐product. Due to very similar solubility properties, the by‐product could not be isolated in pure form for further analysis. We thus hypothesized that the molecular structures resemble each other, and that the by‐product is an isomer of **3**. To shed more light on its possible constitution, we calculated the minimum energies on BP86/SVP[^36^, ^37^, ^38^] level of theory including D3BJ^[39]^ dispersion correction and the ^31^P NMR shifts on TPSS/TZVP[^40^, ^41^] level of theory for the isomers with possible *syn/anti* and *endo/exo* combinations. For simplification, the {Ru(bpy)_3_} fragment was replaced by a phenyl ring (Figure 3).

As can be seen from Figure 3, the *syn‐exo* and *anti‐endo* isomers are almost on the same energy level whereas the *anti‐exo* isomer is energetically disfavored by 8.1 kJ mol^−1^ compared to the *syn*‐*endo* isomer, which is the experimentally observed main isomer. So far, only the exclusive formation of the *endo* products has been reported when employing phosphole derivatives in Diels‐Alder reactions. This observation is supported by DFT calculations concluding that the formation of one of the *endo* isomers is energetically favored.[^42^, ^43^] The calculated ^31^P NMR chemical shift for the *syn*‐*endo* isomer of *δ* ${}^{{}^{31}P}$_calcd_=107 ppm compares reasonably well with the experimental one found for **3** (*δ* ${}^{{}^{31}P}$_exp._=111.4 ppm). Based on the calculated NMR chemical shift of *δ* ${}^{{}^{31}P}$_calcd_=72 ppm for the *anti*‐*endo* isomer it appears reasonable to assume that this isomer is found experimentally (*δ* ${}^{{}^{31}P}$_exp._=77.7 ppm), although it is slightly disfavored by 1.5 kJ mol^−1^ with respect to the *syn*‐*exo* isomer (*δ* ${}^{{}^{31}P}$_calcd_=92 ppm).

**Scheme 5:**
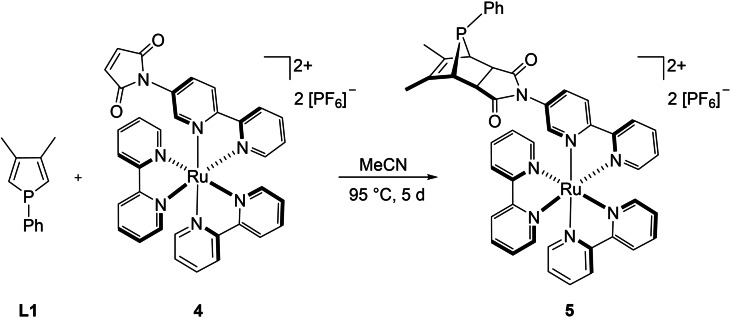
Synthesis of *anti*‐[(**L1**∩**L2**){Ru(bpy)_2_}][PF_6_]_2_ (**5**).

In order to evaluate the influence of the {Ru(bpy)_2_} fragment on the outcome of the Diels‐Alder reaction, *anti*‐[(**L1**∩**L2**){Ru(bpy)_2_}][PF_6_]_2_ (**5**) was synthesized from **L1** and the monometallic ruthenium complex [(**L2**){Ru(bpy)_2_}][PF_6_]_2_ (**4**) by applying the same reaction conditions as for the preparation of the “inverse” gold complex *syn*‐[{AuCl}(**L1**∩**L2**)] (**2**; Scheme 5). The desired product was isolated as red solid in 73 % yield.

Unfortunately, no suitable crystals for X‐ray analysis were obtained. Regardless, the NMR spectroscopic properties are contrasting those of the complexes described above. The ^31^P{^1^H} NMR signal detected at *δ* ${}^{{}^{31}P}$ =49.2 ppm is far less downfield shifted. Besides, the C(O)−*C*H sp^3^ carbon atoms in this complex show weak to no coupling to the phosphorus atom (3.3 and 0 Hz), while the sp^2^ carbon atoms show a large coupling constant of ^2^ *J*_C‐P_=18.5 Hz. These results strongly suggest the formation of the *anti* isomer. This is not surprising as the reaction of trivalent phospholes with dienophiles usually yields the *anti* isomer exclusively or very predominantly.[^25^, ^26^, ^44^] Nevertheless, this example shows the versatility of the applied synthetic strategy and indicates that the coordinated {Ru(bpy)_2_} moiety solitarily does not hamper the Diels‐Alder reaction. Coordination of the {AuCl} fragment to the phosphole seems to suppress the formation of the *anti* product and induces *syn* stereochemistry. The reactivity thus resembles those of phosphole oxides or sulfides.^[43]^ With the {AuCl} fragment oriented towards the {Ru(bpy)_2_} fragment, the approach of the dienophile is obviously hindered rendering the synthesis of *syn*‐[{AuCl}(**L1**∩**L2**){Ru(bpy)_2_}][PF_6_]_2_ (**3**) from the two monometallic precursors **1** and **4** sterically challenging.

**Scheme 6:**
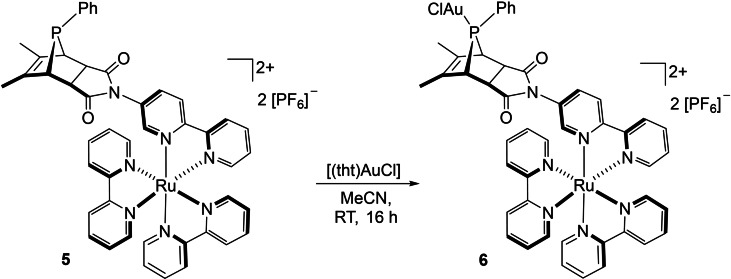
Synthesis of *anti*‐[{AuCl}(**L1**∩**L2**){Ru(bpy)_2_}][PF_6_]_2_ (**6**).

With these results in hand, we were confident to be able to synthesize the *anti* isomer of the heterobimetallic complex on basis of *anti*‐[(**L1**∩**L2**){Ru(bpy)_2_}][PF_6_]_2_ (**5**). Therefore, the monometallic Diels‐Alder product **5** was stirred with [(tht)AuCl] to give *anti*‐[{AuCl}(**L1**∩**L2**){Ru(bpy)_2_}][PF_6_]_2_ (**6**) as a bright red solid in 50 % yield (Scheme 6).

The ^31^P{^1^H} NMR signal of **6** was observed at *δ* ${}^{{}^{31}P}$ =77.7 ppm. This shows that the by‐product in the Diels‐Alder reaction of the monometallic building blocks **1** and **4** is indeed the *anti* isomer, as concluded from the DFT calculations, as it exhibits the same chemical shift as **6**.

**Figure 4:**
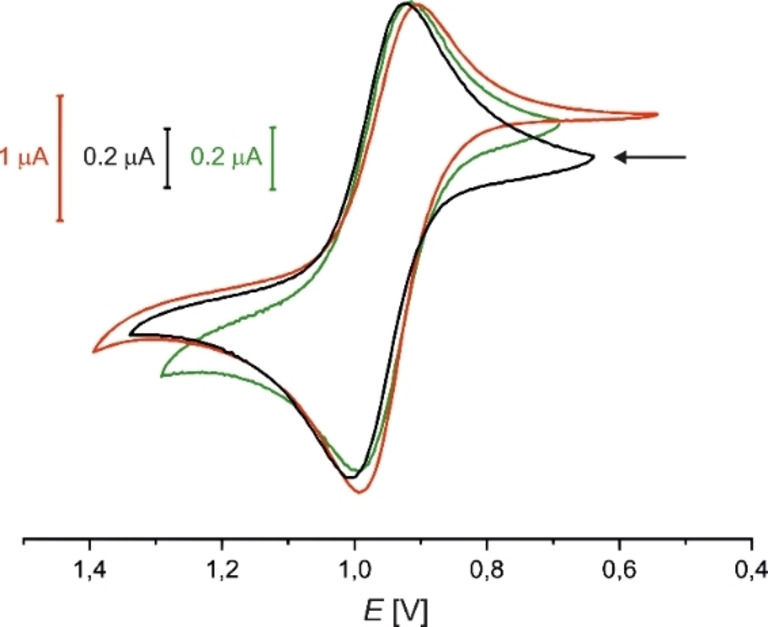
Cyclic voltammograms of **3** (black), **5** (red) and **6** (green) in MeCN vs. Fc/Fc^+^ at room temperature (0.1 M [*n*Bu_4_N][PF_6_]; *v*=250 mV s^−1^; Pt/[*n*Bu_4_N][PF_6_]/Ag).

The redox behavior of the ruthenium‐containing complexes **3** (black), **5** (red) and **6** (green) was investigated with the aid of cyclic voltammetry. Figure 4 shows the quasi‐reversible metal‐based oxidations processes, which are assigned to the Ru^II/III^ redox couple.

For complexes **5** and **6** with *anti* conformation, the E01/2 values were found to be identical at 950 mV, which shows that coordination of the {AuCl} fragment has no influence on the redox potential of the ruthenium center (Table 1).

**Table 1:** Half‐wave potentials, peak potential differences and corresponding *i*_pc_/*i*_pa_ values for the ruthenium‐based oxidation of the synthesized complexes 3, 5 and 6 in MeCN vs. Fc/Fc+ at room temperature.^[a]^

Compound	E01/2 [mV]	Δ*E*_p_ [mV]	*i*_pc_/*i*_pa_
**3** (*syn*)	960	90	∼0.9
**5** (*anti*)	950	100	∼0.8
**6** (*anti*)	950	90	∼0.9

[a] scan rate *v*=250 mV s^−1^, Pt/[*n*Bu_4_N][PF_6_]/Ag.

The redox potential of the bimetallic *syn* complex **3** is slightly anodically shifted by 10 mV, which demonstrates that the *syn*/*anti* isomerism also has a minor influence. The cyclic voltammograms for the reduction of the bipyridyl ligands are shown in Section S2 of the Supporting Information.

**Figure 5:**
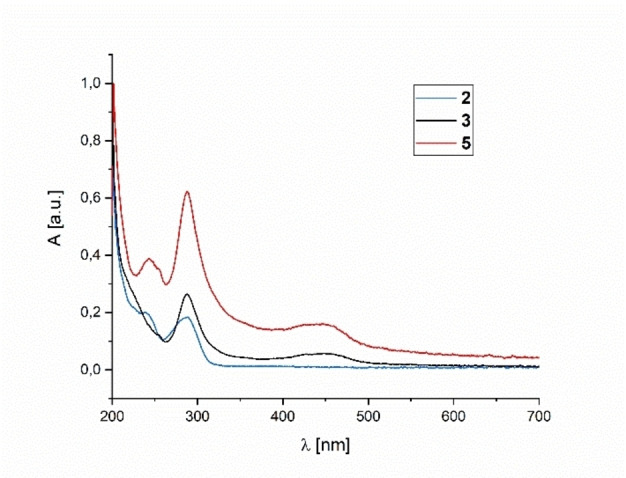
Absorption spectra of **2**, **3**, and **5** in MeCN/EtOH (4 : 1) at 298 K.

To gain further insight into the photophysical properties, absorption spectra of the complexes *syn*‐[{AuCl}(**L1**∩**L2**)] (**2**, blue), *anti*‐[(**L1**∩**L2**){Ru(bpy)_2_}][PF_6_]_2_ (**5**, red) and *syn*‐[{AuCl}(**L1**∩**L2**){Ru(bpy)_2_}][PF_6_]_2_ (**3**, black) were measured in MeCN/EtOH 4 : 1 at 298 K (Figure 5).

Absorption maxima in the UV‐vis spectra and molar extinction coefficients are summarized in Table 2.

**Table 2:** Absorption spectral properties of the metal complexes.^[a]^

Entry	Compound	λabsmax [nm]	*ϵ* [10^5^ M^−1^ cm^−1^]
1	*syn*‐[{AuCl}(**L1**∩**L2**){Ru(bpy)_2_}][PF_6_]_2_ (**3**)	288	1.35
		450	0.27
2	*anti*‐[(**L1**∩**L2**){Ru(bpy)_2_}][PF_6_]_2_ (**5**)	243	0.68
		288	1.10
		446	0.28
3	*syn*‐[{AuCl}(**L1**∩**L2**)] (**2**)	240	0.23
		288	0.21

[a] MeCN/EtOH (4 : 1), 298 K.

The absorption band in the ultraviolet region at 288 nm present in all of the measured complexes arises from a spin‐allowed ligand‐centered (LC) π→π* transition.^[45]^ Additionally, the mononuclear complexes **2** and **5** exhibit a band at 240 and 243 nm, respectively, which can be assigned to intraligand π→π* transitions as well. In the visible region, a band at 446 nm for **5** and a slightly bathochromic shifted one at 450 nm for **3** are found. They are consistent with metal‐to‐ligand charge transfer (MLCT) from the ruthenium atom to the bpy ligands.^[45]^

As the heterobimetallic complex **3** and the monometallic **5** exhibit almost the same molar extinction coefficients, especially for the MLCT bands, neither the coordination of the {AuCl} fragment, nor the stereochemistry at the phosphorous atom has a great impact on the absorption properties of the complexes.

**Figure 6:**
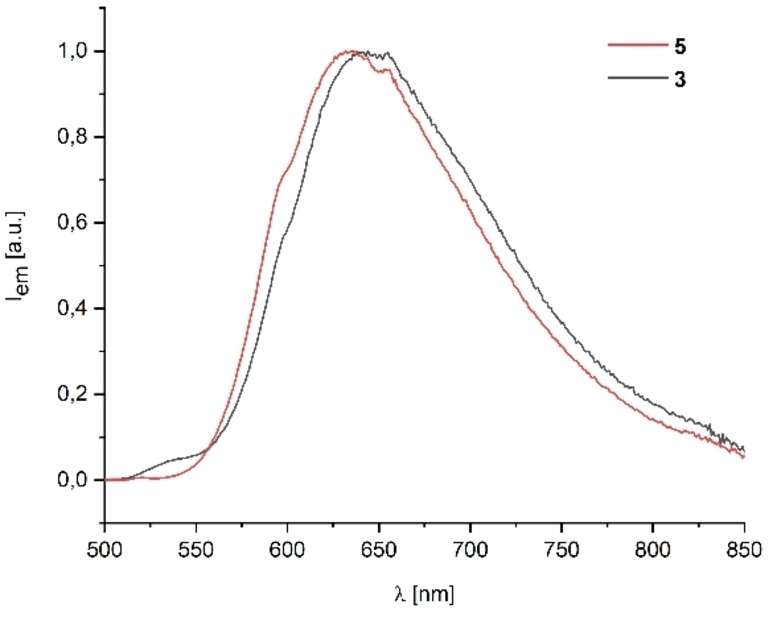
Emission spectra at absorption maximum 450 nm of **3** and **5** in MeCN/EtOH (4 : 1) at 298 K.

Upon excitation at the absorption maximum, **5** gives an emission band at 632 and **3** at 641 nm. Both are significantly red‐shifted and found in the typical region for complexes derived from [Ru(bpy)_3_]^2+^ (Figure 6).^[46]^

As can be seen from Table 3, emission lifetimes and quantum yields of **3** and **5** were measured in MeCN/EtOH (4 : 1) at ambient temperature.

**Table 3:** Optical properties of **3** and **5** in comparison to unmodified [Ru(bpy)_3_][PF_6_]_2_.^[a]^

Entry	Compound	λabsmax [nm]	*Φ*_P_^[b]^	*τ*_P_ [μs]^[c]^
1	[Ru(bpy)_3_][PF_6_]_2_^[d]^	–	0.062^[47]^	0.86^[47]^
2	*syn*‐[{AuCl}(**L1**∩**L2**){Ru(bpy)_2_}][PF_6_]_2_ (**3**)	641	0.047	0.90
3	*anti*‐[(**L1**∩**L2**){Ru(bpy)_2_}][PF_6_]_2_ (**5**)	632	0.043	0.88

[a] MeCN/EtOH, 4 : 1, 298 K. [b] Quantum yield, uncertainty ±0.05. [c] Lifetime of phosphorescence. [d] MeCN.

For both complexes, the decays are monoexponential. The emissive lifetimes are comparable to those obtained for unmodified [Ru(bpy)_3_][PF_6_]_2_ in MeCN (*τ*_P_=0.86 μs at RT) and are supportive that these are ^3^MLCT states in nature.^[47]^ The measured quantum yields of 0.043 for **5** and 0.047 for **3**, respectively, are within the range from *Φ*_P_=0.029 to 0.071 observed for [Ru(bpy)_3_][PF_6_]_2_ in a variety of solvents.^[47]^ Furthermore, they noticeably exceed the photoluminescence efficiency measured for Ru^II^ complexes of ferrocene appended 2,2’‐bipyridine.^[46]^ This indicates that the substitution pattern at the 2,2’‐bipyridine in our ligand system does not facilitate deactivation of ^3^MLCT states. Both the lifetime of phosphorescence and the quantum yield are very similar for the dinuclear complex **3** and its mononuclear counterpart **5**. It is thus clear – and in‐line with the cyclic voltammetry studies – that the influence of the {AuCl} fragment on the photoluminescence properties of the heterobimetallic complex is negligible and that they are dominated by the {Ru(bpy)_3_} moiety. Overall, the observed emission data propose **3** to be a valuable candidate for photocatalytic application. Emission spectra and optical properties of the respective solids in the range from 5 to 295 K can be found in Section S3 of the Supporting Information.

## Application in catalysis

**Scheme 7:**
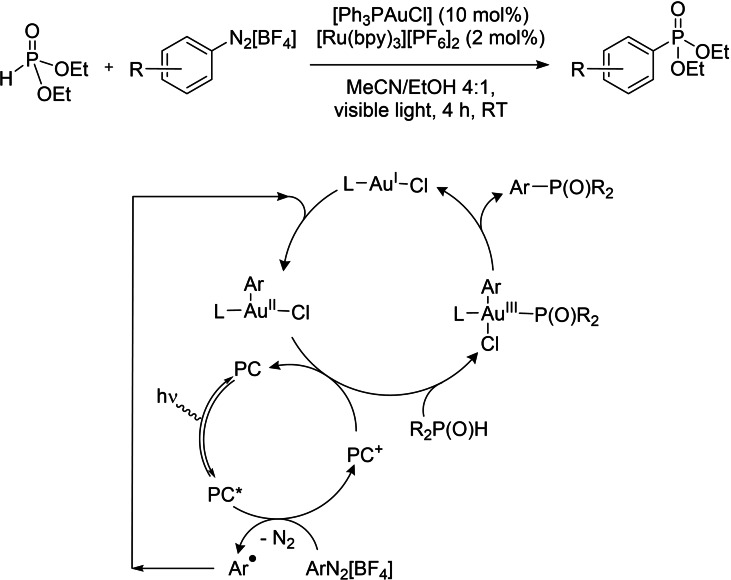
Carbon‐phosphorus photocatalytic cross‐coupling reported by Toste et al.^[15]^ and mechanistic scheme adapted from ref. 11; PC=photocatalyst.

As a model reaction to test the catalytic activity of the bimetallic complexes **3** (*syn*) and **6** (*anti*), a carbon‐phosphorus cross‐coupling was chosen. The resulting organophosphorus compounds are usually accessed through transition metal‐catalyzed coupling processes.^[48]^ More recently, Toste and co‐workers achieved the desired coupling through gold and photoredox catalysis under mild conditions using low‐energy visible light in the absence of base and/or additives at room temperature (Scheme 7).^[15]^

The reaction is proposed to proceed through photoredox‐promoted generation of an aryl gold(III) intermediate from the gold(I) catalyst and the aryldiazonium salt, which undergoes coupling with the *H*‐phosphonate nucleophile.^[11]^

Inspired by these results, we intended to transfer the protocol to our systems and, moreover, to compare the dinuclear complexes with a 1 : 1 mixture of their mononuclear counterparts. Due to easier synthetic access, we initially focused our catalytic studies on **3**. The nature of a bimetallic complex, however, requires a 1 : 1 ratio of Au^I^ to Ru^II^. Hence, an equal amount of the gold(I) catalyst and the generally more active photocatalyst had to be employed. On this account, we added the diazonium salt in portions throughout the reaction to keep the concentration of the aryl radicals generated by the photocatalytic cycle at a moderate level and lowered the reaction time to 3 hours. Indeed, **3** successfully catalyzes the transformation and affords the arylphosphonate **9 a** in 74 % yield (Table 4, entry 1).

**Table 4:** Results of the P‐arylation of aryldiazonium salt **8 a** with *H*‐phosphonate **7**.^[a]^

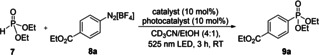				
Entry	Catalyst	Photocatalyst	Conversion [%]^[b]^	Yield [%]^[c]^
1	*syn*‐[{AuCl}(**L1**∩**L2**){Ru(bpy)_2_}][PF_6_]_2_ (**3**)		98	74
2^[d]^	*syn*‐[{AuCl}(**L1**∩**L2**){Ru(bpy)_2_}][PF_6_]_2_ (**3**)		80	33
3^[e]^	*syn*‐[{AuCl}(**L1**∩**L2**){Ru(bpy)_2_}][PF_6_]_2_ (**3**)		68	48
4	none	none	60	0
5	none	[Ru(bpy)_3_][PF_6_]_2_	68	0
6	*syn*‐[{AuCl}(**L1**∩**L2**)] (**2**)	[Ru(bpy)_3_][PF_6_]_2_	78	29
7^[e]^	*syn*‐[{AuCl}(**L1**∩**L2**)] (**2**)	[Ru(bpy)_3_][PF_6_]_2_	58	34
8	*syn*‐[{AuCl}(**L1**∩**L2**)] (**2**)	none	90	66
9^[e]^	*syn*‐[{AuCl}(**L1**∩**L2**)] (**2**)	none	64	36
10	[(**L1**){AuCl}] (**1**)	[Ru(bpy)_3_][PF_6_]_2_	90	45
11^[e]^	[(**L1**){AuCl}] (**1**)	[Ru(bpy)_3_][PF_6_]_2_	54	29
12	[(**L1**){AuCl}] (**1**)	none	86	60
13^[e]^	[(**L1**){AuCl}] (**1**)	none	56	33
14	[Ph_3_PAuCl]	none	82	56
15^[e]^	[Ph_3_PAuCl]	none	39	19

[a] **7** (0.1 mmol), **8 a** (0.2 mmol), after 1 and 2 h another equivalent of **8 a** was added. [b] Determined by ^31^P NMR spectroscopy with triphenylphosphate as internal standard. [c] ^31^P NMR yield. [d] 5 mol% **3**. [e] Reaction run in the dark.

Lowering the catalyst loading to 5 mol% leads to a significantly reduced yield of 33 % (Table 4, entry 2). When carrying out the reaction in the dark still 48 % of **9 a** were formed (entry 3). This is surprising as the photocatalyst should be inactive without irradiation and thus not generating aryl radicals for gold oxidation and subsequent coupling. However, in the absence of a catalyst, or when only [Ru(bpy)_3_][PF_6_]_2_ is employed as photocatalyst, no product can be observed (entries 4 and 5). The conversion in those reactions is still considerably high, which can be attributed to the instability of the diazonium salt in solution that is added in fourfold excess and causes side reactions without the presence a suitable catalyst.

To investigate potential cooperative behavior, the heterobimetallic complex **3** was compared with the catalytic system comprised of a 1 : 1 mixture of the related monometallic complexes **2** and [Ru(bpy)_3_][PF_6_]_2_, which gave only 29 % of arylphosphonate (entry 6). This indicates that having both metal centers in the same scaffold in close spatial proximity to each other plays indeed a crucial role since the yield is even lower than when employing 5 mol% of **3**. Running the reaction in the dark, with or without photocatalyst, gives almost the same yield of 34 and 36 %, respectively (entries 7 and 9), which resembles somewhat what we could already observe for **3**. An unexpectedly high catalytic activity was observed when **2** was used and the mixture was irradiated (66 % yield, Table 4, entry 8). We hypothesized that the high activity can partly be assigned to the uncoordinated bipyridine moiety in the ligand scaffold since bipyridines are known to promote the activation of diazonium salts and have already successfully been applied as additives in this type of reaction.^[49]^ Therefore, we wanted to exclude the possible impact of the attached bipyridine to get a proper comparison of the bimetallic complex to the monometallic system. Moreover, we intended to examine the steric and electronic influence of the ligand backbone formed by the Diels‐Alder reaction. Hence, we additionally conducted the reaction with **1** as gold(I) catalyst and again [Ru(bpy)_3_][PF_6_]_2_ as photocatalyst, which afforded **9 a** in 45 % yield (Table 4, entry 10). Although displaying better activity than the mixture of **2** and [Ru(bpy)_3_][PF_6_]_2_, it is still inferior to the dinuclear catalyst **3**.

Lower yields of 29 and 33 % are obtained when performing the catalysis with **1** in the dark with or without photocatalyst, respectively (entries 11 and 13). Again, employing the gold(I) catalyst **1** under irradiation afforded **9 a** in a comparably good yield of 60 % (entry 12). As all the experiments conducted without photocatalyst (entries 8, 9, 11, 13) and the experiments run with photocatalyst in the dark (entries 3, 7, 11) gave higher yields than we expected, we were intrigued and performed control reactions with our setup using the commonly employed and commercially available [Ph_3_PAuCl] under irradiation and in the dark. Although giving lower yields than the abovementioned gold(I) complexes, the results still display the observed trend (entries 14 and 15). Furthermore, the yields exceed those described in the literature, which can most likely be assigned to different procedure protocols and the light sources that are used.[^15^, ^49^] Nevertheless, these results suggest that other redox processes are taking place in those experiments or other gold‐based mechanistic pathways are contributing to the reaction.

Direct activation of aryldiazonium salts occurs at elevated temperature or under UV irradiation. Initiation under visible light, usually in the range of 450–475 nm, can occur through the formation of weak charge‐transfer complexes with aromatic compounds present in the reaction mixture.^[50]^ Hence, we purposely chose to irradiate with a green LED at 525 nm at room temperature to avoid direct activation of the diazonium salt and minimize side reactions in general. Moreover, the UV‐vis spectrum of an equimolar mixture of the substrates together with triphenylphosphate used as internal NMR standard does not show any absorption above 290 nm (see Section S4 of the Supporting Information). Hashmi and co‐workers also proposed a gold‐induced single electron transfer (SET) to the aryldiazonium salt generating an aryl diazo radical and a gold(II) species, followed by recombination upon irradiation with blue LEDs.^[51]^ Furthermore, the *H*‐phosphonate could act as nucleophilic diazonium activator even in the absence of light. Though these processes are commonly observed at higher temperature, it is also conceivable that a SET from the solvent is involved in radical initiation. Once the catalytic cycle is initiated, the reaction can proceed through a radical chain mechanism.[^49^, ^52^]

As **3** proved to be a good catalyst for the carbon‐phosphorus cross‐coupling, we investigated the scope of the diazonium substrates. The results are summarized in Table 5.

**Table 5:** P‐arylation of various aryldiazonium salts with diethyl phosphite.^[a]^

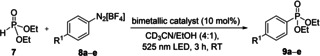				
Entry	R^1^	Catalyst	Conversion [%]^[b]^	Yield [%]^[c]^
1	CO_2_Et	**3** (*syn*)	98	**9 a**, 74
2	OEt	**3** (*syn*)	>99	**9 b**, 89 (92)^[d]^
3	OEt	**6** (*anti*)	>99	**9 b**, 91
4	Me	**3** (*syn*)	>99	**9 c**, 84
5	Br	**3** (*syn*)	>99	**9 d**, 78
6	H	**3** (*syn*)	88	**9 e**, 63
7	H	**6** (*anti*)	94	**9 e**, 70

[a] **7** (0.1 mmol), **8 a**–**e** (0.2 mmol), after 1 and 2 h another equivalent of **8 a**–**e** was added, respectively. [b] Determined by ^31^P NMR spectroscopy with triphenylphosphate as internal standard. [c] ^31^P NMR yield. [d] Reaction was carried out in a Schlenk tube under stirring: **7** (0.4 mmol), **8 b** (0.8 mmol), after 1 and 2 h another equivalent of **8 b** was added, respectively. Non‐deuterated MeCN was used. Isolated yield.

Aryldiazonium salts bearing electron‐donating groups such as ethoxy and methyl in the *para* position were coupled with diethyl phosphite affording the corresponding products **9 b** and **9 c** in excellent yields of 89 and 84 %, respectively (Table 5, entries 2 and 4). When the reaction employing **8 b** was performed on preparative scale, even 92 % of product could be isolated, which can partly be assigned to stirring throughout the reaction in this experiment (Table 5, entry 2). This result is consistent with those of the Toste group.^[15]^ A slight decrease in yield was observed when diazonium salt **8 a**, bearing an electron withdrawing group, or diazonium salt **8 d**, containing a bromide in the *para*‐position, were used (Table 5, entries 1 and 5). The lowest yields were obtained for the unsubstituted benzene diazonium salt **8 e** affording 63 % of **9 e** (Table 5, entry 6).

We were also interested in investigating how a change of the M⋅⋅⋅M distance within the heterobimetallic complex affects the catalytic activity. On this account we repeated the experiments using the *anti* isomer **6** with an increased distance of estimated 11.5 Å (cf. ∼11 Å for **3**) between the gold and the ruthenium atom as catalyst. For comparison, the 4‐ethoxyphenyldiazonium salt **8 b**, which gave the best results for **3** (*syn*), and the benzene diazonium salt **8 e**, which was the worst substrate, were chosen. In both reactions, **6** (*anti*) gave slightly better results than the *syn* isomer **3**. 91 % of **9 b** could be obtained with **6** (Table 5, entry 3), whereas **3** afforded 89 % yield (Table 5, entry 1). The difference in activity becomes more pronounced when the less reactive substrate **8 e** was used in the reaction. While the *syn* isomer **3** gave 63 % of the corresponding product (Table 5, entry 6), 70 % of **9 e** were obtained with the *anti* isomer **6** as catalyst (Table 5, entry 7). These results contrast the expectation that decreasing the distance between the catalytic centers would facilitate the reaction. In fact, Bräse and co‐workers could observe the same trend when probing different isomers of their bimetallic Au^I^/Ru^III^ cyclophanyl complexes in a Meyer‐Schuster rearrangement. Decreasing the M⋅⋅⋅M distance led to a drop in yield, showing that electronic parameters and steric effects have to be considered as well.^[17]^ Nonetheless, our studies have demonstrated that the heterobimetallic complexes overall achieve better results in the investigated carbon‐phosphorus cross‐coupling than their mononuclear counterparts. This clearly indicates that having both metal centers in close proximity to each other and a predefined spatial orientation appears to be beneficial for the outcome of the reaction. Preliminary time‐dependent reaction profile studies support the view that the effect is due to kinetic reasons (see Figure S12 in the Supporting Information). However, the exact nature of the observed effect is not clear at this stage.

## Conclusion

In this work, we have presented the synthesis of two isomeric heterobimetallic complexes *syn*‐ and *anti*‐[{AuCl}(**L1**∩**L2**){Ru(bpy)_2_}][PF_6_]_2_ (**3** and **6**) by applying a post‐functionalization strategy through a Diels‐Alder reaction. With this strategy, we were able to obtain both isomers exclusively and selectively by altering the synthetic route. We were able to examine how the stereochemistry and, consequently, the different M⋅⋅⋅M distances influence the properties of the heterodinuclear complexes. The electrochemical behavior of the synthesized ruthenium‐containing compounds **3** (*syn*), **5** (*anti*), and **6** (*anti*) was investigated revealing a small influence of the stereochemistry on the redox properties. The photophysical data show that the *syn* isomer **3** possesses a low energy absorption at 450 nm, moderate efficiency of photoluminescence (*Φ*_P_=0.047) and a relatively long‐lived excited state (*τ*_P_=0.9 μs); these qualify the complex as valuable for application in photoredox catalysis.

The performance of both complexes **3** and **6** was evaluated in a dual gold photoredox catalysis and compared to 1 : 1 mixtures of the monometallic relatives. The investigated carbon‐phosphorus cross‐coupling was successfully catalyzed by **3** and even better by **6**, with yields of up to 91 %. Moreover, both dinuclear complexes outperform their monometallic counterparts. These results show that incorporating different metals into the same ligand scaffold, each executing precise sequences of multiple reactions in a one‐pot fashion, is most desirable.

The influence of modulating the metal‐to‐metal distance in complexes that combine multiple metal centers in a single molecule on the activity and selectivity in catalysis is yet to be understood. Further investigations to that end are an important objective to develop heterobimetallic catalysts for broader applicability. As we are confident that these results can be transferred to other reactions catalyzed by a dual metal system, attempts to expand the synthetic approach to combine other metals in one ligand backbone are ongoing in our laboratories.

## Experimental Section

**General methods and materials**: All manipulations, except of aqueous workups or unless noted otherwise, were carried out with standard Schlenk techniques. Air‐sensitive compounds were stored and weighed in a glovebox (Braun MB150 G‐I and Unilab system). Methylene chloride and acetonitrile were freshly distilled under argon from calcium hydride, methanol was distilled from NaOMe and ethanol from sodium/diethyl phthalate. Toluene, benzene, diethyl ether, *n*‐hexane and xylene were dried using sodium/benzophenone ketyl. [D_8_]THF and CDCl_3_ were purchased from commercial sources and used as received. CD_3_CN was vacuum transferred from calcium hydride into thoroughly dried glassware equipped with Young Teflon valves, while [D_6_]DMSO was dried over 4 Å molecular sieves. Reagents were purchased from commercial sources and used as received. 5‐Amino‐2,2‘‐bipyridine,[^30^, ^31^] 3,4‐dimethyl‐1‐phenylphosphole (dmpp, **L1**),^[29]^ chloro‐3,4‐dimethyl‐1‐phenylphosphole gold(I) ([(**L1**){AuCl}], **1**),^[24]^ [Ru(bpy)_2_Cl_2_],^[53]^ and diazonium salts^[13]^ were synthesized according to literature procedures.

NMR spectra were measured on an Avance Neo 400 or an Avance 300 spectrometer. ^1^H and ^13^C chemical shifts are referred to TMS, those of ^31^P to H_3_PO_4_, those of ^19^F to CFCl_3_. Coupling constants *J* are given in Hz as positive values regardless of their real individual signs. NMR samples were prepared in oven‐dried 5‐mm NMR tubes. Air or moisture sensitive samples were prepared inside a glovebox using screw cap or Young valve NMR tubes. Unless otherwise stated, standard Bruker software routines (TOPSPIN and XWIN NMR) were used for the 1D and 2D NMR measurements. IR spectra were measured with a Bruker Alpha spectrometer using the attenuated total reflection (ATR) technique on powdered samples, and the data are quoted in wavenumbers (cm^−1^). The UV‐vis spectra were recorded using a Mettler‐Toledo spectrophotometer UV7 and quartz cuvettes (*d*=1 cm) for solutions. To subtract the solvent, the sample was measured relative to the pure solvent. Elemental analyses were done by the institutional technical laboratories of the Karlsruhe Institute of Technology (KIT).

Cyclic voltammetry measurements were performed with a Metrohm potentiostat (PGSTAT101) and an electrochemical cell inside a glovebox. A freshly polished Pt disk working electrode, a Pt wire as counter electrode and a Ag wire as (pseudo)reference electrode were used ([^*n*^Bu_4_N][PF_6_] (0.1 M) as electrolyte). Potentials were calibrated against the ferrocene/ferrocenium (Fc/Fc^+^) couple as internal standard.

**Synthesis of 5‐nitro‐2,2’‐bipyridine**: 5‐Nitro‐2,2’‐bipyridine was prepared by a modified literature procedure. A 2‐neck Schlenk‐flask equipped with a reflux condenser was charged with 2‐bromo‐5‐nitropyridin (2.74 g, 13.51 mmol), 2‐(tributylstannyl)pyridine (3.50 mL, 3.98 g, 10.81 mmol), [Pd(PPh_3_)_4_] (0.312 g, 0.270 mmol) and 60 mL xylene (mixture of isomers). The reaction mixture was heated to 130 °C under stirring. After 24 h, the mixture was cooled and poured into 2 M NaOH. The phases were separated, and the aqueous layer was extracted with toluene (3×50 mL). The combined organic phases were dried over MgSO_4_ and evaporated under reduced pressure. The crude product was filtered through a silica gel bed (eluted with methylene chloride) and subsequently purified by silica gel chromatography, eluting with methylene chloride, to afford 5‐nitro‐2,2’‐bipyridine (1.72 g, 79 %) as an orange solid. The ^1^H NMR spectrum is in accordance with literature.^[30]^

**Synthesis of** ***N*** **‐bipyridinylmaleamic acid**: A 2‐neck‐flask equipped with a reflux condenser and a dropping funnel was charged with maleic anhydride (0.624 g, 6.37 mmol) and 60 mL diethyl ether. 5‐Amino‐2,2’‐bipyridine (1.09 g, 6.37 mmol) in 60 mL diethyl ether was added dropwise and the reaction mixture was allowed to stir for 1 h at room temperature. After filtration the solvent was removed under reduced pressure. The crude product was recrystallized from diethyl ether to yield the desired product (1.13 g, 66 %) as a yellow solid.

^1^H NMR (300 MHz, [D_8_]THF): *δ*=10.48 (s, 1H, O*H*), 8.79 (d, ^4^ *J*_HH_=2.6 Hz, 1H, *H*_bpy_), 8.58–8.60 (m, 1H, *H*_bpy_), 8.47 (dd, ^3^ *J*_HH_=8.7 Hz, ^5^ *J*_HH_ *=* 0.7 Hz, 1H, *H*_bpy_), 8.42 (dt, ^3^ *J*_HH_=8.0 Hz, ^4^ *J*_HH_=1.1 Hz, 1H, *H*_bpy_), 8.28 (dd, ^3^ *J*_HH_=8.7 Hz, ^4^ *J*_HH_=2.5 Hz, 1H, *H*_bpy_), 7.79 (td, ^3^ *J*_HH_=7.7 Hz, ^4^ *J*_HH_=1.8 Hz, 1H, *H*_bpy_), 7.24–7.29 (m, 1H, *H*_bpy_), 6.47 (d, ^3^ *J*_HH_=12.6 Hz, 1H, C=C*H*), 6.28 ppm (d, ^3^ *J*_HH_=12.7 Hz, 1H, C=C*H*). ^13^C{^1^H} NMR (75 MHz, [D_8_]THF): *δ*=166.5 (*C*OOH), 164.9 (*C*ONH), 156.9 (*C*_bpy_), 152.9 (*C*_bpy_), 150.1 (*C*H_bpy_), 141.6 (*C*H_bpy_), 137.5 (*C*_bpy_), 136.6 (*C*H_bpy_), 135.2 (C=CH), 131.5 (C=CH), 128.1 (*C*H_bpy_), 124.2 (*C*H_bpy_), 121.6 (*C*H_bpy_), 121.1 ppm (*C*H_bpy_). IR‐ATR *ν*=3024 (vw), 1684 (vw), 1613 (s), 1573 (m), 1549 (s), 1465 (m), 1435 (w), 1397 (m), 1374 (m), 1327 (vw), 1270 (m), 1243 (m), 1203 (m), 1121 (w), 1096 (w), 1037 (m), 989 (vw), 937 (w), 894 (vw), 853 (vs), 792 (vs), 742 (s), 731 (s), 663 (s), 637 (vs), 555 (vw), 511 (m), 459 (m), 415 (vw), 401 cm^−1^ (m). Elemental analysis calcd (%) for C_14_H_11_N_3_O_3_: C 62.45, H 4.12, N 15.61; found: C 62.22, H 4.05, N 15.68.

**Synthesis of L2**: A 2‐neck flask equipped with a reflux condenser was charged with *N*‐bipyridylmaleamic acid (1.21 g, 4.50 mmol), sodium acetate (0.185 g, 2.25 mmol) and acetic anhydride (7.32 mL, 7.90 g, 77.4 mmol). The reaction mixture was heated to 100 °C under stirring. After 30 min, the mixture was cooled to room temperature and poured into ice water. The resulting precipitate was filtered and washed with ice water and *n*‐hexane. The crude product was recrystallized from cyclohexane to afford **L2** (0.741 g, 66 %) as a yellow solid. ^1^H NMR (300 MHz, CDCl_3_): *δ*=8.76 (dd, ^4^ *J*_HH_=2.5 Hz, ^5^ *J*_HH_=0.8 Hz, 1H, *H*_bpy_), 8.68–8.71 (m, 1H, *H*_bpy_), 8.53 (dd, ^3^ *J*_HH_=8.6 Hz, ^5^ *J*_HH_=0.8 Hz, 1H, *H*_bpy_), 8.43 (dt, ^3^ *J*_HH_=8.0 Hz, ^5^ *J*_HH_=1.1 Hz, 1H, *H*_bpy_), 7.92–7.79 (m, 2H, *H*_bpy_), 7.31–7.35 (m, 1H, *H*_bpy_), 6.93 ppm (s, 2H, C=C*H*). ^13^C{^1^H} NMR (75 MHz, CDCl_3_): *δ*=169.0 (*C*=O), 155.4 (*C*_bpy_), 155.1 (*C*_bpy_), 149.4 (*C*H_bpy_), 145.9 (*C*H_bpy_), 137.1 (*C*H_bpy_), 134.7 (C=CH), 133.7 (*C*H_bpy_), 128.5(*C*_bpy_), 124.1 (*C*H_bpy_), 121.4 (*C*H_bpy_), 121.3 ppm (*C*H_bpy_). IR ATR: *ν*=3064 (vw), 1718 (vs), 1587 (vw), 1557 (w), 1495 (m), 1463 (m), 1434 (w), 1395 (s), 1374 (m), 1320 (vw), 1297 (vw), 1247 (vw), 1215 (vw), 1133 (m), 1079 (vw), 1061 (w), 1045 (vw), 1029 (vw), 991 (vw), 948 (vw), 925 (vw), 850 (w), 837 (s), 791 (w), 763 (vw), 738 (vs), 691 (vs), 645 (w), 589 (w), 494 (vw), 479 (vw), 462 (vw), 398 cm^−1^ (m). Elemental analysis calcd (%) for C_14_H_9_N_3_O_2_ **⋅** 1/3 H_2_O: C 65.37, H 3.79, N 16.33; found: C 65.04, H 3.46, N 16.23.

**Synthesis of 2**: A Schlenk tube was charged with **1** (204.8 mg, 0.487 mmol), **L2** (122.3 mg, 0.487 mmol) and 8 mL acetonitrile. The reaction mixture was heated to 95 °C under stirring. After 5 days, the mixture was cooled to room temperature. The precipitate was filtered and washed with 3 mL acetonitrile to yield *syn*‐[{AuCl}(**L2**∩**L1**)] (210.4 mg, 64 %) as a colorless solid. ^1^H NMR (300 MHz, [D_6_]DMSO): *δ*=8.71–8.73 (m, 1H, *H*_bpy_), 8.53 (dd, ^3^ *J*_HH_=8.5 Hz, ^5^ *J*_HH_=0.8 Hz, 1H, *H*_bpy_), 8.44–8.34 (m, 2H, *H*_bpy_), 7.98 (td, ^3^ *J*_HH_=7.5 Hz, ^4^ *J*_HH_=1.9 Hz, 1H, *H*_bpy_), 7.76–7.64 (m, 3H, *H*_bpy_, *H*_Phenyl_), 7.61–7.46 (m, 4H, *H*_bpy_, *H*_Phenyl_), 4.25 (s, 2H, C(O)−C*H*), 4.10 (s, 2H, P‐C*H*), 1.57 ppm (d, ^4^ *J*_PH_=1.1 Hz, 6H, C*H*_3_). ^31^P{^1^H} NMR (121 MHz, [D_6_]DMSO): *δ*=110.4 ppm (s). ^13^C{^1^H} NMR (75 MHz, [D_6_]DMSO): *δ*=174.3 (d, ^3^ *J*_PC_=17.3 Hz, *C*=O), 155.2 (*C*_bpy_), 154.2 (*C*_bpy_), 149.5 (*C*H_bpy_), 146.8 (*C*H_bpy_), 137.5 (*C*H_bpy_), 135.7 (*C*H_bpy_), 132.7 (*C*=C), 131.7 (d, ^3^ *J*_PC_=10.7 Hz, *C*H_Phenyl_), 131.6 (d, ^4^ *J*_PC_=2.4 Hz, *C*H_Phenyl_), 129.0 (d, ^2^ *J*_PC_=10.8 Hz, *C*H_Phenyl_), 128.9 (d, ^5^ *J*_PC_=1.6 Hz, *C*_bpy_), 127.1 (d, ^1^ *J*_PC_=55.4 Hz, *C*_Phenyl_), 124.7 (*C*H_bpy_), 121.0 (*C*H_bpy_), 120.8 (*C*H_bpy_), 49.3 (d, ^1^ *J*_PC_=38.4 Hz, P−*C*H), 45.8 (d, ^2^ *J*_PC_=24.4 Hz, C(O)−*C*H), 14.8 ppm (d, ^3^ *J*_PC_=3.0 Hz, *C*H_3_). IR ATR: *ν*=1780 (vw), 1713 (vs), 1588 (vw), 1575 (vw), 1558 (vw), 1489 (w), 1457 (m), 1437 (w), 1398 (m), 1336 (vw), 1301 (vw), 1283 (vw), 1244 (vw), 1222 (vw), 1194 (s), 1144 (w), 1116 (vw), 1091 (vw), 1062 (vw), 1024 (vw), 992 (vw), 968 (vw), 878 (vw), 858 (vw), 827 (vw), 797 (m), 753 (m), 745 (m), 717 (w), 691 (s), 645 (vw), 627 (w), 559 (vs), 515 (w), 449 (s), 399 cm^−1^ (w). Elemental analysis calcd (%) for C_26_H_22_N_3_O_2_PAuCl: C 46.48, H 3.30, N 6.25; found: C 46.53, H 3.21, N 6.21.

**Synthesis of 3**: A Schlenk tube was charged with **2** (63.1 mg, 0.094 mmol), [Ru(bpy)_2_Cl_2_] (54 mg, 0.113 mmol), Ag[BF_4_] (37.5 mg, 0.193 mmol) and 20 mL methylene chloride. The reaction mixture was heated to reflux under stirring. After 6 days, the solution was cooled to room temperature, filtered through Celite and the solvent was removed under reduced pressure. The solid was taken up in 7 mL acetonitrile and ammonium hexafluorophosphate (45.9 mg, 0.282 mmol) in 1.5 mL methanol was added. After stirring at room temperature over night, the solvent was removed under reduced pressure. The solid was washed with degassed water, dissolved in 1.5 mL acetonitrile, and crystallized by slow diffusion of the acetonitrile solution into benzene to yield the product (101.1 mg, 78 %) as orange prisms/ crystals. ^1^H NMR (300 MHz, CD_3_CN): *δ*=8.59–8.45 (m, 6H, *H*_bpy_), 8.11–8.02 (m, 5H, *H*_bpy_), 8.01–7.94 (m, 1H„ *H*_bpy_), 7.79–7.82 (m, 1H, *H*_bpy_), 7.76–7.68 (m, 4H„ *H*_bpy_), 7.59–7.47 (m, 6H, *H*_bpy,_ *H*_Phenyl_), 7.45–7.32 (m, 5H, *H*_bpy,_ *H*_Phenyl_), 4.22–4.15 (m, 2H, C(O)−C*H*), 3.78–3.74 (m, 2H, P‐C*H*), 1.31 ppm (d, ^4^ *J*_PH_=9.7 Hz, 6H, C*H*_3_). ^31^P{^1^H} NMR (121 MHz, CD_3_CN): *δ*=111.4 (s), −144.6 ppm (sept, ^1^ *J*_FP_ = 706.6 Hz). ^19^F{^1^H} NMR (282 MHz, CD_3_CN): *δ*=−72.81 ppm (d, ^1^ *J*_PF_=706.7 Hz). ^13^C{^1^H} NMR (101 MHz, CD_3_CN): *δ*=174.4 (d, ^3^ *J*_PC_=17.4 Hz, *C*=O), 174.3 (d, ^3^ *J*_PC_=17.2 Hz, *C*=O) 158.1 (*C*_bpy_), 157.9 (*C*_bpy_), 157.9 (*C*_bpy_), 157.9 (*C*_bpy_), 157.5 (*C*_bpy_), 157.1 (*C*_bpy_), 152.9 (*C*H_bpy_), 152.8 (*C*H_bpy_), 152.8 (*C*H_bpy_), 152.8 (*C*H_bpy_), 152.6 (*C*H_bpy_), 148.6 (*C*H_bpy_), 139.1 (*C*H_bpy_), 139.1 (*C*H_bpy_), 139.0 (*C*H_bpy_), 139.0 (*C*H_bpy_), 138.9 (*C*H_bpy_), 135.3 (*C*H_bpy_), 134.0 (*C*=C), 133.9 (*C*=C), 132.9 (d, ^4^ *J*_PC_=2.5 Hz, *C*H_Phenyl_) 132.8 (d, ^3^ *J*_PC_=11.0 Hz, *C*H_Phenyl_), 132.1 (d, ^5^ *J*_PC_=1.9 Hz, *C*_bpy_), 130.2 (d, ^2^ *J*_PC_=10.9 Hz, *C*H_Phenyl_),129.9 (*C*H_bpy_), 129.2 (*C*H_bpy_), 128.9 (*C*H_bpy_), 128.8 (*C*H_bpy_), 128.7 (*C*H_bpy_), 128.6 (*C*H_bpy_)., 127.6 (d, ^1^ *J*_PC_=55.8 Hz, *C*_Phenyl_),126.3 (*C*H_bpy_), 125.9 (*C*H_bpy_), 125.5 (*C*H_bpy_), 125.4 (*C*H_bpy_), 125.2 (*C*H_bpy_), 50.9 (d, ^1^ *J*_PC_=38.7 Hz, P‐*C*H), 46.7 (d, ^2^ *J*_PC_=24.3 Hz, C(O)−*C*H), 46.7 (d, ^2^ *J*_PC_=24.3 Hz, C(O)−*C*H) 15.5 (d, ^3^ *J*_PC_=3.1 Hz, *C*H_3_), 15.5 ppm (d, ^3^ *J*_PC_=3.1 Hz, *C*H_3_). IR ATR: *ν*=1782 (vw), 1719 (m), 1605 (vw), 1578 (vw), 1499 (vw), 1466 (w), 1446 (w), 1387 (w), 1314 (vw), 1275 (vw), 1243 (vw), 1210 (vw), 1189 (w), 1156 (vw), 1114 (vw), 1064 (vw), 962 (vw), 895 (vw), 877 (vw), 829 (vs), 782 (w), 762 (s), 744 (m), 729 (w), 689 (w), 660 (vw), 647 (vw), 623 (vw), 608 (vw), 555 (vs), 449 (w), 431 (vw), 396 cm^−1^ (w). Elemental analysis calcd (%) for C_46_H_38_AuClF_12_N_7_O_2_P_3_Ru⋅1/2 C_6_H_6_: C 41.61, H 2.92, N 6.93; found: C 41.40, H 2.79, N 7.04.

**Synthesis of 4**: A Schlenk tube was charged with **L2** (100 mg, 0.398 mmol), [Ru(bpy)_2_Cl_2_] (231.3 mg, 0.478 mmol), Ag[BF_4_] (158.8 mg, 0.816 mmol) and 24 mL methylene chloride. The reaction mixture was heated to reflux and allowed to stir over night. The solution was cooled to room temperature, filtered through Celite and the solvent was removed under reduced pressure. The solid was taken up in 10 mL acetonitrile and ammonium hexafluorophosphate (194.6 mg, 1.19 mmol) in 2 mL methanol was added. After stirring at room temperature overnight, the solvent was removed under reduced pressure. The crude product was washed with degassed water (2×5 mL), dissolved in 6 mL acetonitrile, and precipitated with diethyl ether to afford **4** (263.5 mg, 69 %) as an orange solid. ^1^H NMR (300 MHz, CD_3_CN): *δ*=8.62–8.46 (m, 6H, *H*_bpy_), 8.17 (dd, ^3^ *J*_HH_=8.9 Hz, ^4^ *J*_HH_=2.2 Hz, 1H, *H*_bpy_), 8.13–8.01 (m, 5H, *H*_bpy_), 7.88–7.67 (m, 6H, *H*_bpy_), 7.47–7.36 (m, 5H, *H*_bpy_), 6.91 ppm (s, 2H, C=C*H*). ^31^P{^1^H} NMR (121 MHz, CD_3_CN): *δ*=−144.6 ppm (sept, ^1^ *J*_FP_=706.7 Hz). ^19^F{^1^H} NMR (282 MHz, CD_3_CN): *δ*=−72.92 ppm (d, ^1^ *J*_PF_=706.5 Hz). ^13^C{^1^H} NMR (75 MHz, CD_3_CN): *δ*=169.4 (*C*=O), 158.0 (*C*_bpy_), 158.0 (*C*_bpy_), 157.9 (*C*_bpy_), 157.9 (*C*_bpy_), 157.4 (*C*_bpy_), 155.6 (*C*_bpy_), 152.8 (*C*H_bpy_), 152.8 (*C*H_bpy_), 152.6 (*C*H_bpy_), 152.6 (*C*H_bpy_), 152.5 (*C*H_bpy_), 147.7 (*C*H_bpy_), 138.9 (*C*H_bpy_), 138.8 (*C*H_bpy_), 138.8 (*C*H_bpy_), 136.0 (C=*C*H), 133.6 (*C*H_bpy_), 132.7 (*C*_bpy_), 128.6 (*C*H_bpy_), 128.5 (*C*H_bpy_), 128.5 (*C*H_bpy_), 125.4 (*C*H_bpy_), 125.3 (*C*H_bpy_), 125.1 ppm (*C*H_bpy_). IR ATR: *ν*=1722 (m), 1604 (vw), 1575 (vw), 1498 (vw), 1466 (w), 1446 (w), 1398 (w), 1382 (w), 1315 (vw), 1275 (vw), 1245 (vw), 1138 (vw), 1070 (vw), 1030 (vw), 967 (vw), 896 (vw), 878 (vw), 829 (vs), 760 (s), 729 (m), 694 (w), 661 (vw), 648 (vw), 633 (vw), 599 (vw), 555 (vs), 422 (vw), 402 cm^−1^ (vw). Elemental analysis: due to the high fluorine content of this compound, no elemental analysis could be performed.

**Synthesis of 5**: A Schlenk tube was charged with **4** (80.0 mg, 0.0838 mmol), **L1** (23.7 mg, 0.126 mmol) and 6 mL acetonitrile. The reaction mixture was heated to reflux under stirring. After 5 days, the solution was cooled to room temperature, the solvent was reduced to a minimal amount and the product was precipitated with diethyl ether as a red solid (69.6 mg, 73 %). ^1^H NMR (300 MHz, CD_3_CN): *δ*=8.56–8.42 (m, 7H, *H*_bpy_), 8.14–7.98 (m, 6H, *H*_bpy_), 7.90 (dd, ^3^ *J*_HH_=8.8 Hz, ^4^ *J*_HH_=2.2 Hz, 1H, *H*_bpy_), 7.84–7.63 (m, 6H, *H*_bpy_), 7.52 (dd, ^4^ *J*_HH_=2.2, ^5^ *J*_HH_=0.6 Hz, 1H, *H*_bpy_), 7.48–7.23 (m, 11H, *H*_bpy_, *H*_Phenyl_), 3.40–3.28 (m, 4H, P‐C*H*, C(O)−C*H*), 1.51 ppm (dp, ^4^ *J*_PH_=10.9 Hz, ^5^ *J*_HH_=1.2 Hz, 6H, C*H*_3_). ^31^P{^1^H} NMR (162 MHz, CD_3_CN): *δ*=49.2, −144.6 ppm (sept, ^1^ *J*_FP_=706.6 Hz). ^19^F{^1^H} NMR (282 MHz, CD_3_CN): *δ*=−72.86 ppm (d, ^1^ *J*_PF_=706.6 Hz). ^13^C{^1^H} NMR (101 MHz, CD_3_CN): *δ*=176.6 (*C*=O), 158.0 (*C*_bpy_), 157.9 (*C*_bpy_), 157.9 (*C*_bpy_),157.9 (*C*_bpy_), 157.2 (*C*_bpy_), 157.0 (*C*_bpy_), 152.9 (*C*H_bpy_), 152.8 (*C*H_bpy_), 152.8 (*C*H_bpy_), 152.7 (*C*H_bpy_), 152.6 (*C*H_bpy_), 148.6 (*C*H_bpy_), 142.2 (d, ^1^ *J*_PC_=37.8 Hz, *C*_Phenyl_), 139.1 (*C*H_bpy_), 139.0 (*C*H_bpy_), 139.0 (*C*H_bpy_), 139.0 (*C*H_bpy_), 138.9 (*C*H_bpy_),135.2 (*C*H_bpy_), 135.1 (d, ^2^ *J*_PC_=18.5 Hz, *C=C*), 135.0 (d, ^2^ *J*_PC_=18.5 Hz, *C=C*), 132.5 (*C*_bpy_), 130.1 (d, ^3^ *J*_PC_=2.5 Hz, *C*H_Phenyl_), 129.7 (d, ^2^ *J*_PC_=12.0 Hz, *C*H_Phenyl_), 129.2 (*C*H_Phenyl_), 128.8 (*C*H_bpy_), 128.7 (*C*H_bpy_), 128.7 (*C*H_bpy_), 128.6 (*C*H_bpy_), 128.6 (*C*H_bpy_), 125.8 (*C*H_bpy_), 125.4 (*C*H_bpy_), 125.4 (*C*H_bpy_), 125.3 (*C*H_bpy_), 125.2 (*C*H_bpy_), 50.0 (C(O)−*C*H), 49.9 (d, ^2^ *J*_PC_=3.3 Hz, C(O)−*C*H), 49.9 (d, ^1^ *J*_PC_=13.8 Hz, P‐*C*H), 15.6 (d, ^3^ *J*_PC_=1.3 Hz, *C*H_3_), 15.6 ppm (d, ^3^ *J*_PC_=1.3 Hz, *C*H_3_). IR‐ATR: *ν*=1713 (m), 1604 (vw), 1497 (vw), 1464 (w), 1445 (w), 1370 (vw), 1313 (vw), 1243 (vw), 1206 (vw), 1160 (vw), 877 (vw), 829 (vs), 759 (m), 729 (m), 695 (w), 661 (vw), 647 (vw), 619 (vw), 555 (vs), 495 (vw), 464 (w), 410 (vw), 384 cm^−1^ (vw). Elemental analysis calcd (%) for C_46_H_38_F_12_N_7_O_2_P_3_Ru: C 48.35, H 3.35, N 8.58; found: C 48.03, H 3.386, N 8.34.

**Synthesis of 6**: A Schlenk tube was charged with [(tht)AuCl] (22.4 mg, 0.070 mmol) in 5 mL acetonitrile. **5** (80.0 mg, 0.070 mmol) in 3 mL acetonitrile was added dropwise. The reaction mixture was stirred at room temperature for 16 h. Afterwards the solvent was reduced. The crude product was precipitated with diethyl ether and washed with toluene to afford **6** as bright red solid (48 mg, 50 %). ^1^H NMR (300 MHz, CD_3_CN): *δ*=8.57–8.42 (m, 6H, *H*_bpy_), 8.13–7.99 (m, 5H, *H*_bpy_), 7.91 (dd, ^3^ *J*_HH_=8.8 Hz, ^4^ *J*_HH_=2.2 Hz, 1H, *H*_bpy_), 7.83–7.77 (m, 1H, *H*_bpy_), 7.76–7.66 (m, 4H, *H*_bpy_), 7.60–7.52 (m, 5H, *H*_Phenyl_), 7.50 (d, ^4^ *J*_HH_=2.1 Hz, 1H, *H*_bpy_), 7.46–7.33 (m, 6H, *H*_bpy_), 3.85–3.79 (m, 2H, P‐C*H*), 3.44–3.36 (m, 2H, C(O)−C*H*), 1.56 ppm (d, ^4^ *J*_PH_=10.6 Hz, 6H, C*H*_3_). ^31^P{^1^H} NMR (121 MHz, CD_3_CN): *δ*=77.7 (s), −144.6 ppm (sept, ^1^ *J*_FP_=706.7 Hz). ^19^F{^1^H} NMR (282 MHz, CD_3_CN): *δ*=−72.87 ppm (d, ^1^ *J*_PF_=706.5 Hz). ^13^C{^1^H} NMR (101 MHz, CD_3_CN): *δ*=174.9 (d, ^3^ *J*_PC_=11.3 Hz, *C*=O), 174.9 (d, ^3^ *J*_PC_=11.0 Hz, *C*=O), 158.0 (*C*_bpy_), 157.9 (*C*_bpy_), 157.9 (*C*_bpy_), 157.9 (*C*_bpy_), 157.4 (*C*_bpy_), 157.1 (*C*_bpy_), 152.9 (*C*H_bpy_), 152.8 (*C*H_bpy_), 152.8 (*C*H_bpy_), 152.8 (*C*H_bpy_),152.7 (*C*H_bpy_), 152.6 (*C*H_bpy_), 148.6 (*C*H_bpy_), 139.2 (*C*H_bpy_), 139.1 (*C*H_bpy_), 139.0 (*C*H_bpy_), 139.0 (*C*H_bpy_), 138.9 (*C*H_bpy_), 138.8 (*C*H_bpy_), 135.5 (d, ^2^ *J*_PC_=11.0 Hz, *C*=C),135.4 (d, ^2^ *J*_PC_=11.3 Hz, *C*=C), 135.2 (*C*H_bpy_), 133.0 (d, ^4^ *J*_PC_=2.1 Hz, *C*H_Phenyl_), 132.1 (*C*_bpy_), 131.9 (d, ^1^ *J*_PC_=25.9 Hz, *C*_Phenyl_), 130.9 (d, ^3^ *J*_PC_=9.5 Hz, *C*H_Phenyl_), 130.6 (d, ^2^ *J*_PC_=11.1 Hz, *C*H_Phenyl_),128.9 (*C*H_bpy_), 128.8 (*C*H_bpy_), 128.7 (*C*H_bpy_), 128.6 (*C*H_bpy_), 128.6 (*C*H_bpy_), 125.9 (*C*H_bpy_), 125.5 (*C*H_bpy_), 125.4 (*C*H_bpy_), 125.2 (*C*H_bpy_), 49.2 (d, ^1^ *J*_PC_=36.7 Hz, P−*C*H), 47.0 (d, ^2^ *J*_PC_=9.7 Hz, C(O)−*C*H), 47.0 (d, ^2^ *J*_PC_=9.8 Hz, C(O)−*C*H), 16.0 (d, ^3^ *J*_PC_=2.5 Hz, *C*H_3_), 16.0 ppm (d, ^3^ *J*_PC_=2.5 Hz, *C*H_3_). IR ATR: *ν*=1719 (vw), 1466 (vw), 1445 (vw), 1376 (vw), 1171 (vw), 834 (vs), 786 (vw), 762 (w), 729 (w), 713 (vw), 697 (vw), 661 (vw), 623 (vw), 585 (vw), 556 (vs), 486 (w), 467 (vw), 446 (vw), 428 (vw), 418 (vw), 407 (vw), 400 (vw), 390 (vw), 380 cm^−1^ (vw). Elemental analysis calcd (%) for C_46_H_38_AuClF_12_N_7_O_2_P_3_Ru**⋅** 1/2 (C_2_H_5_)_2_O: C 40.82, H 3.07, N 6.94; found: C 41.21, H 2.725, N 6.95.

**General procedure for P‐arylation of** ***H*** **‐phosphonate**: An oven‐dried Young NMR tube or brown glass NMR tube was charged with ethyl 4‐carboxybenzenediazonium tetrafluoroborate (53.9 mg, 0.204 mmol), triphenylphosphate (33.3 mg, 0.102 mmol), 0.50 mL of a 0.204 M solution of diethylphosphonate (14.1 mg, 0.102 mmol) in CD_3_CN and 0.12 mL ethanol in the dark. After measuring a ^31^P NMR spectrum for reference, 10 mol% of the catalyst were added and the solution was either irradiated with a green 20 W LED light‐source (525 nm) or remained in the dark for 3 h at room temperature. After the first and second hour, another equivalent of diazonium salt was added. NMR yields were obtained by ^31^P NMR analysis of the crude mixture with the internal standard of triphenylphosphate.

**Testing effects of various diazoniumsalts**: An oven‐dried Young NMR tube was charged with diazonium salt (0.202 mmol, 2 equiv.), triphenylphosphate (33.0 mg, 0.101 mmol), 0.50 mL of a 0.202 M solution of diethylphosphonate (13.9 mg, 0.101 mmol) in CD_3_CN and 0.12 mL ethanol in the dark. After measuring the ^31^P NMR spectrum for reference, 10 mol% of **3** or **6** (13.9 mg, 0.0101 mmol) were added and the solution was irradiated with a green 20 W LED light‐source (525 nm) for 3 h at room temperature. After the first and second hour of irradiation, another equivalent of diazonium salt was added. NMR yields were obtained by ^31^P NMR analysis of the crude mixture with the internal standard of triphenylphosphate.

**Diethyl (4‐ethoxyphenyl)phosphonate (9 b)**: An oven‐dried Schlenk tube was charged with 4‐ethoxybenzenediazonium tetrafluoroborate (193.5 mg, 0.820 mmol) **8 b**, **3** (56.4 mg, 0.041 mmol), 2.00 mL of a 0.205 M solution of diethylphosphonate (56.6 mg, 0.410 mmol) in MeCN and 0.50 mL ethanol in the dark. The solution was irradiated with a green 20 W LED light‐source (525 nm) for 3 h at room temperature. After the first and second hour of irradiation, another equivalent of diazonium salt **8 b** was added. Afterwards, the mixture was quenched with water and aqueous K_2_CO_3_ solution, extracted with methylene chloride, dried over Na_2_SO_4_, filtered, and concentrated in vacuum. The crude product was purified by column chromatography over silica gel using *n*‐hexane and ethyl acetate as eluent to afford **9 b** (97.2 mg, 92 %) as an orange oil. ^1^H NMR (300 MHz, CDCl_3_): *δ*=7.80–7.66 (m, 2H, *H*_Phenyl_), 6.98–6.90 (m, 2H, *H*_Phenyl_), 4.20–3.95 (m, 6H, C*H*_2_), 1.43 (t, ^3^ *J*_HH_=7.0 Hz, 3H, Ph−O−CH_2_−C*H*_3_), 1.31 ppm (td, ^3^ *J*_HH_=7.1 Hz, ^4^ *J*_PH_=0.6 Hz, 6H, P−O‐CH_2_‐C*H*_3_). ^31^P{^1^H} NMR (121 MHz, CDCl_3_): *δ*=19.9 ppm.

**Crystallographic details**: Crystallographic data, data collection, and refinement details can be found in Section S1 of the Supporting Information. Deposition Numbers 2179061 (for **2**) and 2179060 (for **3**) contain the supplementary crystallographic data for this paper. These data are provided free of charge by the joint Cambridge Crystallographic Data Centre and Fachinformationszentrum Karlsruhe Access Structures service.

**Quantum chemical calculations**: For the calculations we used the ORCA 4.2 program.^[54]^ The DFT[^55^, ^56^] calculations were carried out with the functional BP86[^36^, ^37^] or TPSS^[40]^ and the basis set def2‐SVP^[38]^ or def2‐TZVP^[41]^ including D3BJ dispersion correction.^[39]^ Calculations were done with the following settings of calculation parameters: gridsize^[56]^ 4, threshold for SCF energy change 10^‐6^; convergence thresholds for the structure optimization: energy change 10^−6^ gradient norm 10^−4^.

## Conflict of interest

The authors declare no conflict of interest.

1

## Supporting information

As a service to our authors and readers, this journal provides supporting information supplied by the authors. Such materials are peer reviewed and may be re‐organized for online delivery, but are not copy‐edited or typeset. Technical support issues arising from supporting information (other than missing files) should be addressed to the authors.

Supporting Information

## Data Availability

The data that support the findings of this study are available in the supplementary material of this article.
