# Supplementary material for: Heterobimetallic Gold/Ruthenium Complexes Synthesized via Post‐functionalization and Applied in Dual Photoredox Gold Catalysis
Source: Chemistry. 2022 Sep 1;28(57):e202201856. doi: 10.1002/chem.202201856 (PMC9804866; doi:10.1002/chem.202201856)
Supplement: Supplementary file 1 — Supporting Information [file CHEM-28-0-s001.pdf]

Chemistry–A European Journal

Supporting Information

Heterobimetallic Gold/Ruthenium Complexes Synthesized via Post-functionalization and Applied in Dual Photoredox Gold Catalysis

Lea Bayer, Bernhard S. Birenheide, Felix Krämer, Sergei Lebedkin, and Frank Breher*

Supporting Information

Table of Contents

S1. Crystallographic data 1
S2. Cyclic voltammetry 3
S3. Photoluminescence measurements 4
S4 UV-vis spectrum of catalysis substrates 9
S5. Kinetic studies 10
S6. NMR spectra 11
S7. Coordinates of calculated compounds 27
References 34

S1. Crystallographic data

Crystals suitable for X-ray diffraction were obtained in the manner described in the Experimental Section. In order to avoid quality degradation, the single crystals were mounted in perfluoropolyalkylether oil on top of a Mitegen holder loop and then brought into the cold nitrogen stream of a low-temperature device (Oxford Cryosystems Cryostream unit), so that the oil solidified. Diffraction data were measured using a Stoe IPDS II diffractometer and graphite-monochromated MoKα (0.71073 Å) radiation or Stoe STADIVARI diffractometer and GaKα (1.34134 Å) radiation. Absorption corrections were carried out using the STOE LANA[1] software package. Structure solution were carried out using OLEX2 1.3[2] by dual-space direct methods with SHELXT[3] or SHELXS,[4] by full-matrix least-squares refinement using SHELXL-2014/7.[5] All non-hydrogen atoms were refined anisotropically. The contribution of the hydrogen atoms, in their calculated positions, was included in the refinement using a riding model.

*Table S1: Crystal data and structure refinement for **2** and **3**.*

Compund	**2**	**3**
Empirical formula	$C_{26}H_{22}AuClN_3O_2P$	$C_{55}H_{47}AuClF_{12}N_7O_2P_3Ru$
Formula weight	671.85	1492.39
Temperature/K	180	200
Crystal system	monoclinic	monoclinic
Space group	$P2_1/a$	$P2_1/c$
a/Å	7.7933(6)	17.0573(6)
b/Å	17.5099(12)	15.8800(4)
c/Å	18.0481(16)	21.9608(7)
α/°	90	90
β/°	91.963(6)	110.903(3)
γ/°	90	90
Volume/Å^3	2461.4(3)	5557.0(3)
Z	4	4
ρ_{calc}g/cm^3	1.813	1.784
μ/mm^{-1}	9.174	3.133
F(000)	1304.0	2940.0
Crystal size/mm^3	0.15 × 0.06 × 0.01	0.2 × 0.167 × 0.1
Radiation	GaKα (λ = 1.34143)	MoKα (λ = 0.71073)
2Θ range for data collection/°	6.12 to 104.95	3.244 to 51.996
Index ranges	-9 ≤ h ≤ 3, -20 ≤ k ≤ 20, -21 ≤ l ≤ 21	-21 ≤ h ≤ 21, -19 ≤ k ≤ 17, -27 ≤ l ≤ 27
Reflections collected	19857	23849
Independent reflections	19857 [R_{int} = 0.111, R_{sigma} = 0.1702]	10919 [R_{int} = 0.0328, R_{sigma} = 0.0426]
Data/restraints/parameters	19857/114/310	10919/157/766
Goodness-of-fit on F^2	0.943	1.084
Final R indexes [I>=2σ (I)]	R_1 = 0.1087, wR_2 = 0.2765	R_1 = 0.0490, wR_2 = 0.0947
Final R indexes [all data]	R_1 = 0.2067, wR_2 = 0.3332	R_1 = 0.0763, wR_2 = 0.1077
Largest diff. peak/hole / e Å^{-3}	4.52/-2.71	1.93/-1.96
CCDC	2179061	2179060

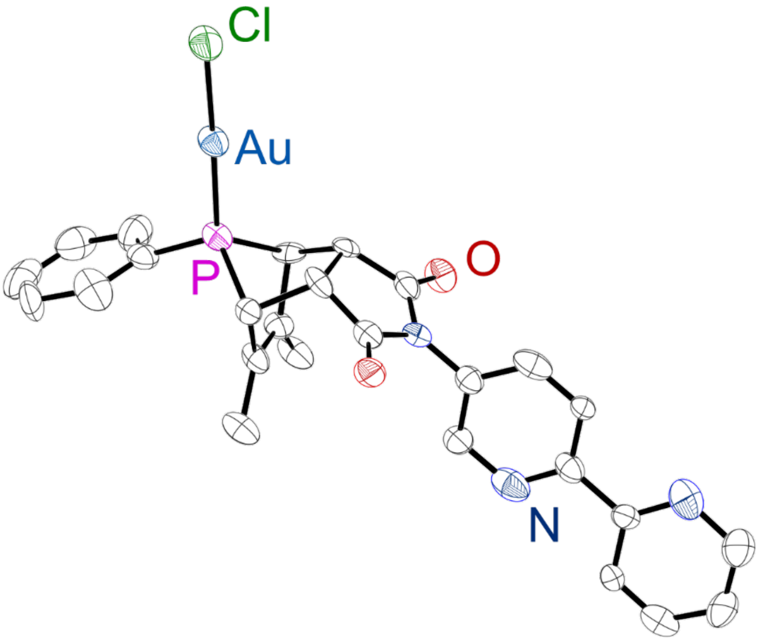

*Figure S1: Molecular structure of syn-[{AuCl}(**L2**∩**L1**)] (**2**). Hydrogen atoms have been omitted for clarity.*

S2. Cyclic voltammetry

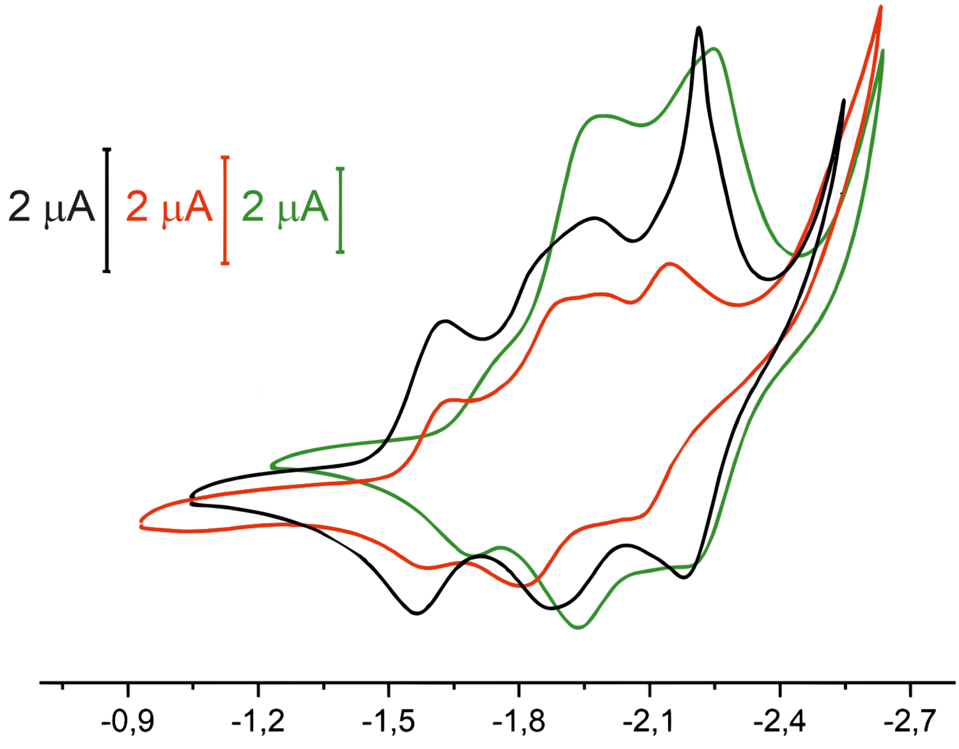

*Figure S2: Cyclic voltammograms of **3** (black), **5** (red) and **6** (green) in MeCN vs. Fc/Fc$^+$ at room temperature. Electrolyte: 0.1M [^{n}Bu$_4$N][PF$_6$], scan rate v = 250 mV/s.*

*Table S2: Half-wave potentials and peak potential differences for the bipyridyl-based reductions of the synthesized complexes **3**, **5** and **6** in MeCN vs. Fc/Fc+ at room temperature.*[a]

Compound	$E^0_{1/2}$ [V]	ΔE_p [mV]
**3** (*syn*)	-2.190	40
	-1.870	100
	-1.580	60
**5** (*anti*)	-2.130	70
	-1.880	70
	-1.620	40
**6** (*anti*)	-2.210	70
	-1.950	50
	-1.660	50

[a] scan rate v = 250 mV/s, Pt/[^{n}Bu$_4$N][PF$_6$]/Ag.

S3. Photoluminescence measurements

Photoluminescence (PL) measurements were performed with a Horiba Jobin Yvon Fluorolog-322 spectrometer equipped with a closed-cycle optical cryostat operating within a temperature range of ca. 5-300 K. The solid samples (crystalline powders) were measured as dispersions in a thin layer of viscous polyfluoroester oil placed between two 1 mm quartz plates. The latter were mounted on the cold finger of the cryostat. Solutions were transferred into a standard sealable cuvette and argon-purged before measurements. All emission spectra were corrected for the wavelength-dependent response of the spectrometer and detector (in relative photon flux units). Emission decay traces were recorded by connecting the detector (photomultiplier) to a fast oscilloscope (via a 50, 500 or 2.500 Ohm load depending on the decay time scale) and using a nitrogen laser (ca. 2 nsec, 5 µJ per pulse) for pulsed excitation. PL efficiencies of solid complexes at ambient temperature were determined with an integrating sphere out of optical PTFE, which was installed into the sample chamber of the spectrometer. PL efficiencies of solutions at ambient temperature were referred to that of Rhodamine 6G in ethanol (taken as 0.95). The uncertainty of these measurements was estimated to be ±10% and ±5%, respectively.

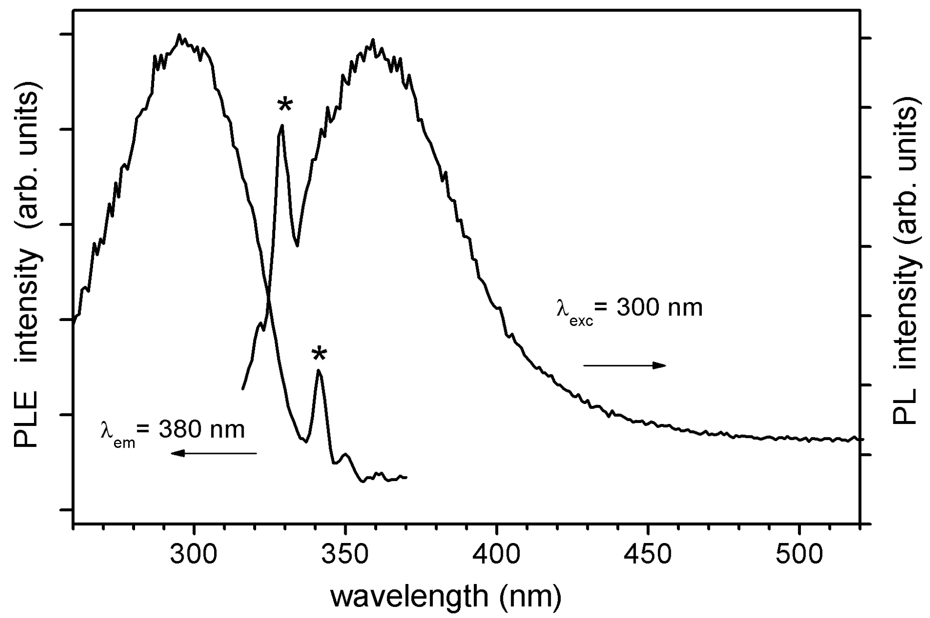

*Figure S3:Emission spectrum of syn-[{AuCl}(**L2**∩**L1**)] (**2**) in MeCN/EtOH 4:1 (c = 3.86*10^{-6} M) at ambient temperature upon excitation at 300 nm. * = Raman signal of solvent.*

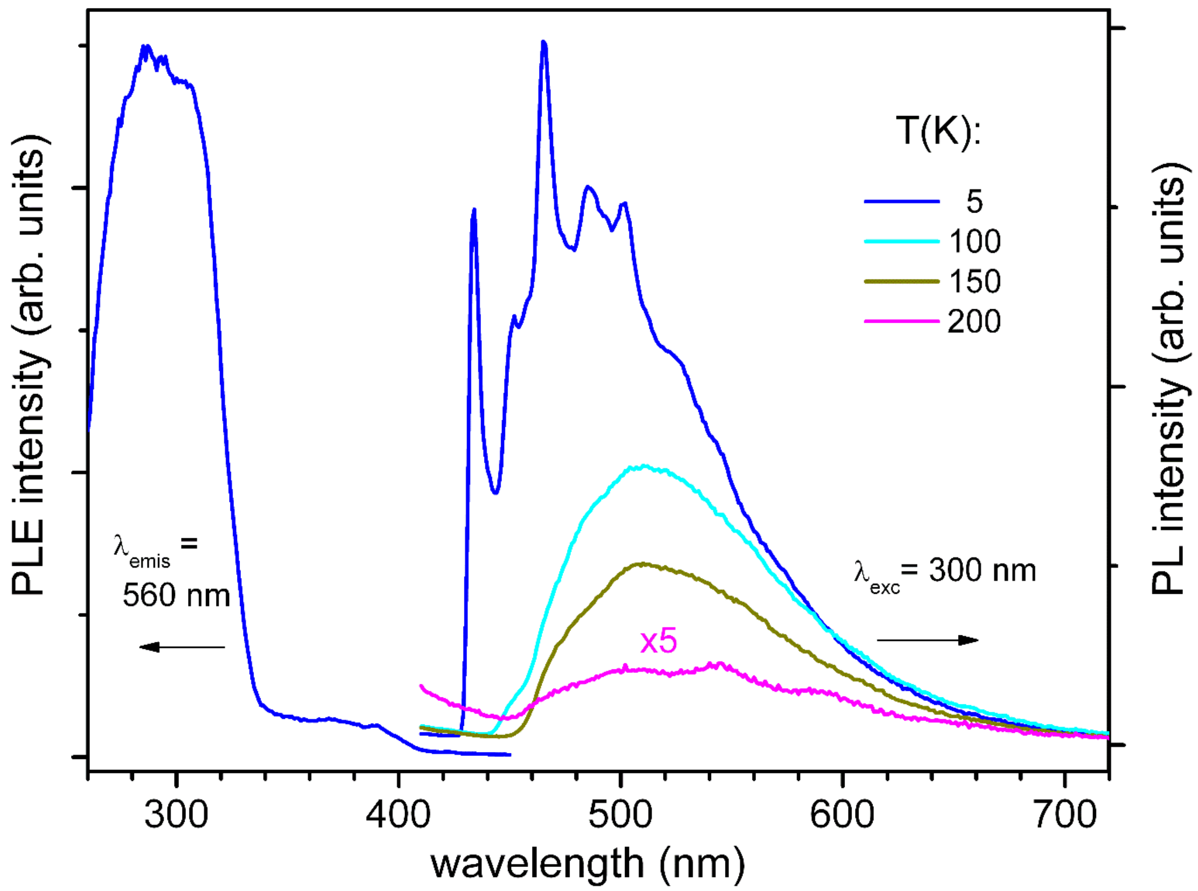

*Figure S4: Emission spectra of solid syn-[{AuCl}(**L2**∩**L1**)] (**2**) at 5 - 298 K; Excitation at 300 nm.*

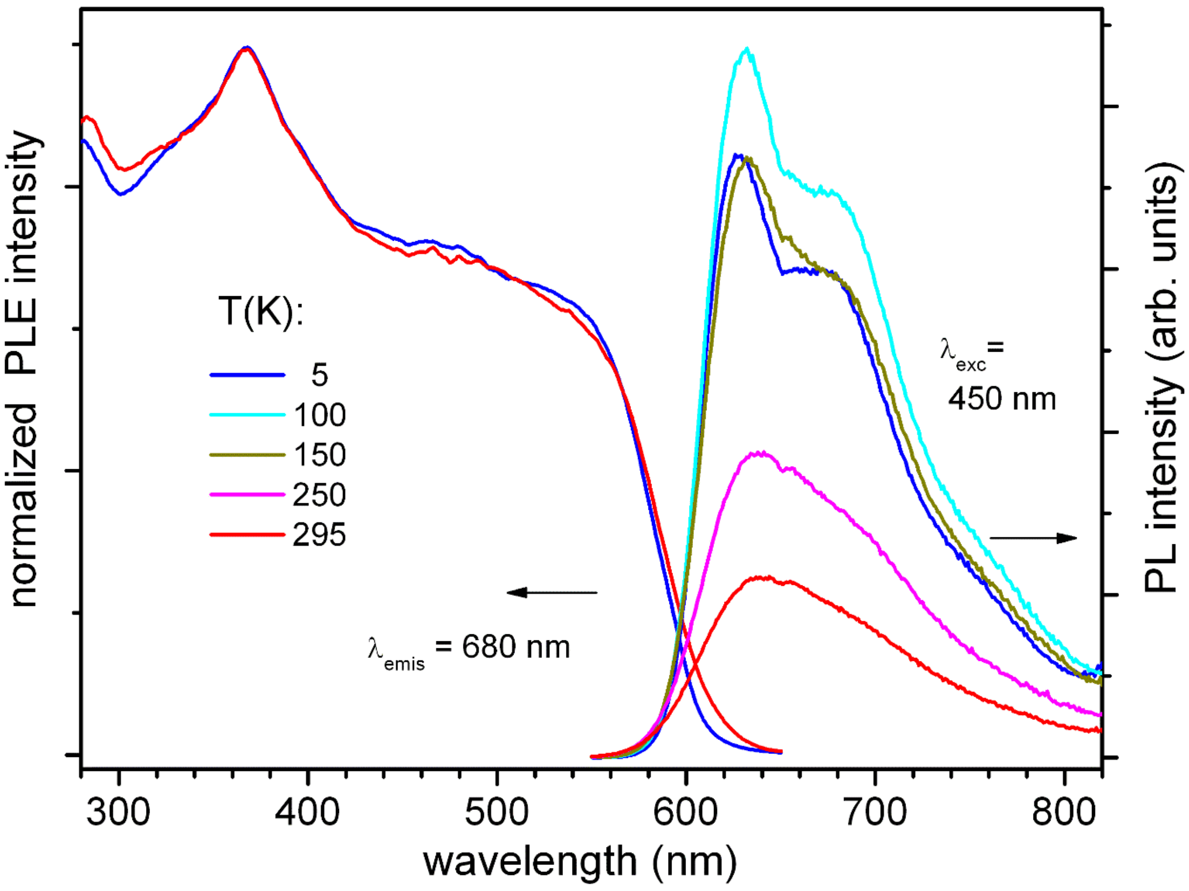

*Figure S5: Emission spectra at absorption maximum 450 nm of solid anti-[(**L1**∩**L2**){Ru(bpy)$_2$}][PF$_6$]$_2$ (**5**) at 5 - 298 K.*

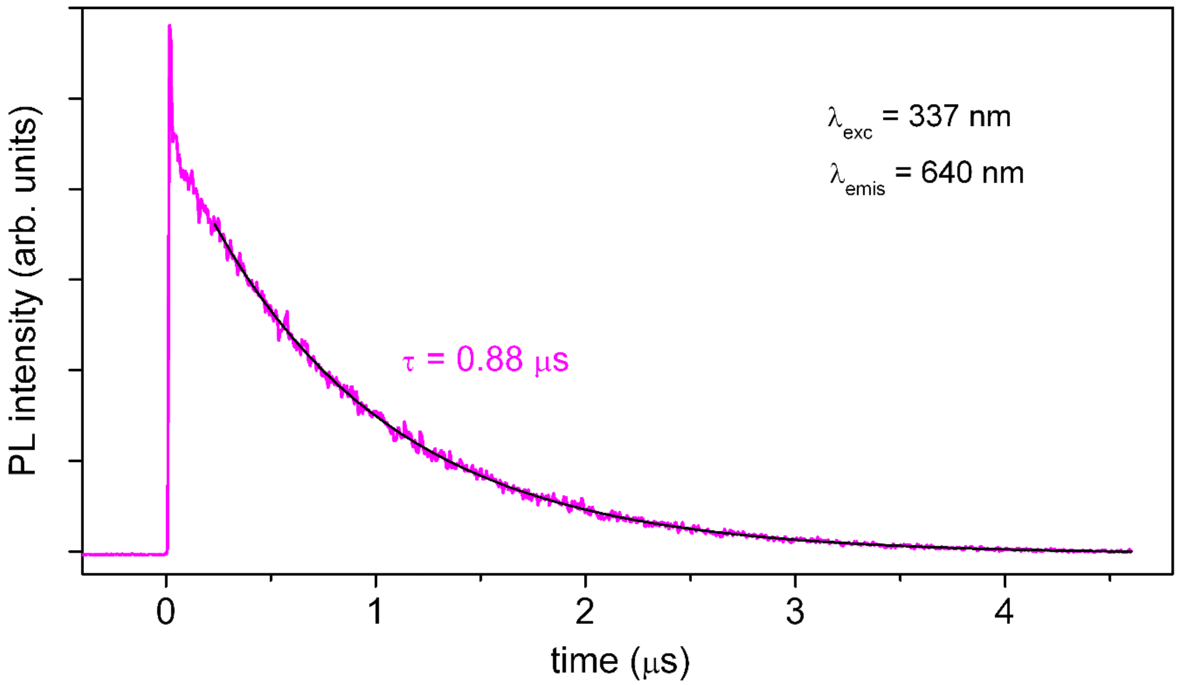

*Figure S6:Decay of anti-[(**L1**∩**L2**){Ru(bpy)$_2$}][PF$_6$]$_2$ (**5**) in MeCN/EtOH 4:1 (C = 3.68*10^{-6} M) at ambient temperature.*

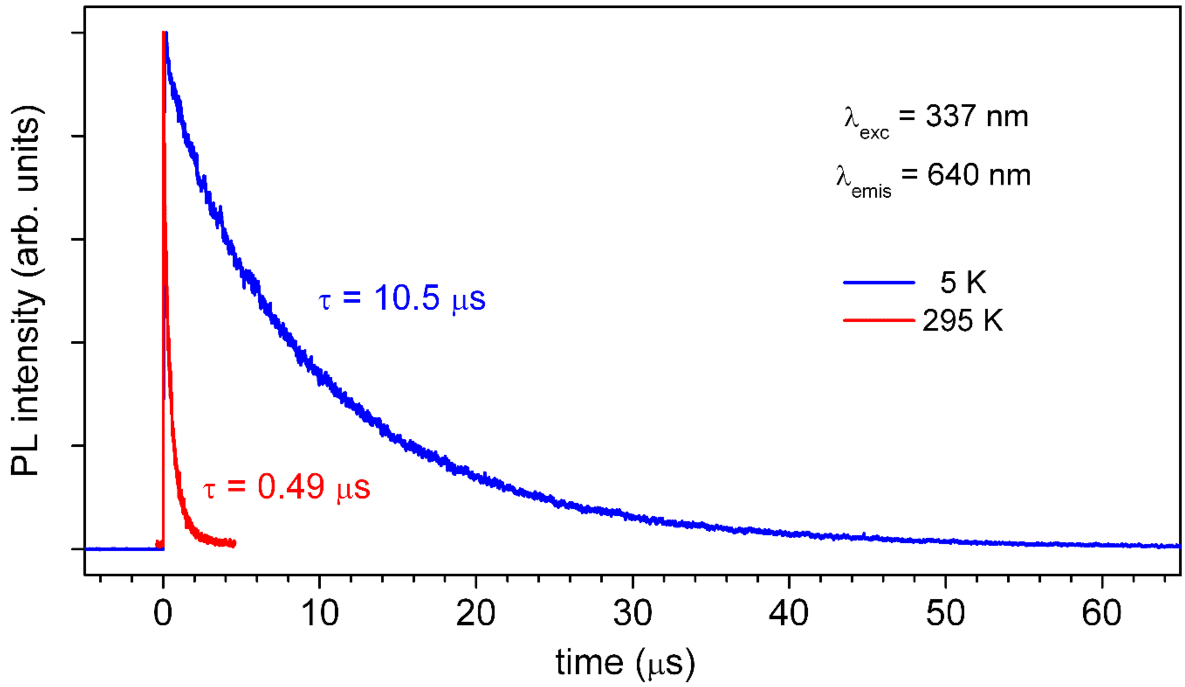

*Figure S7: Decays in solid state of anti-[(**L1**∩**L2**){Ru(bpy)$_2$}][PF$_6$]$_2$ (**5**) at 5 and 295 K.*

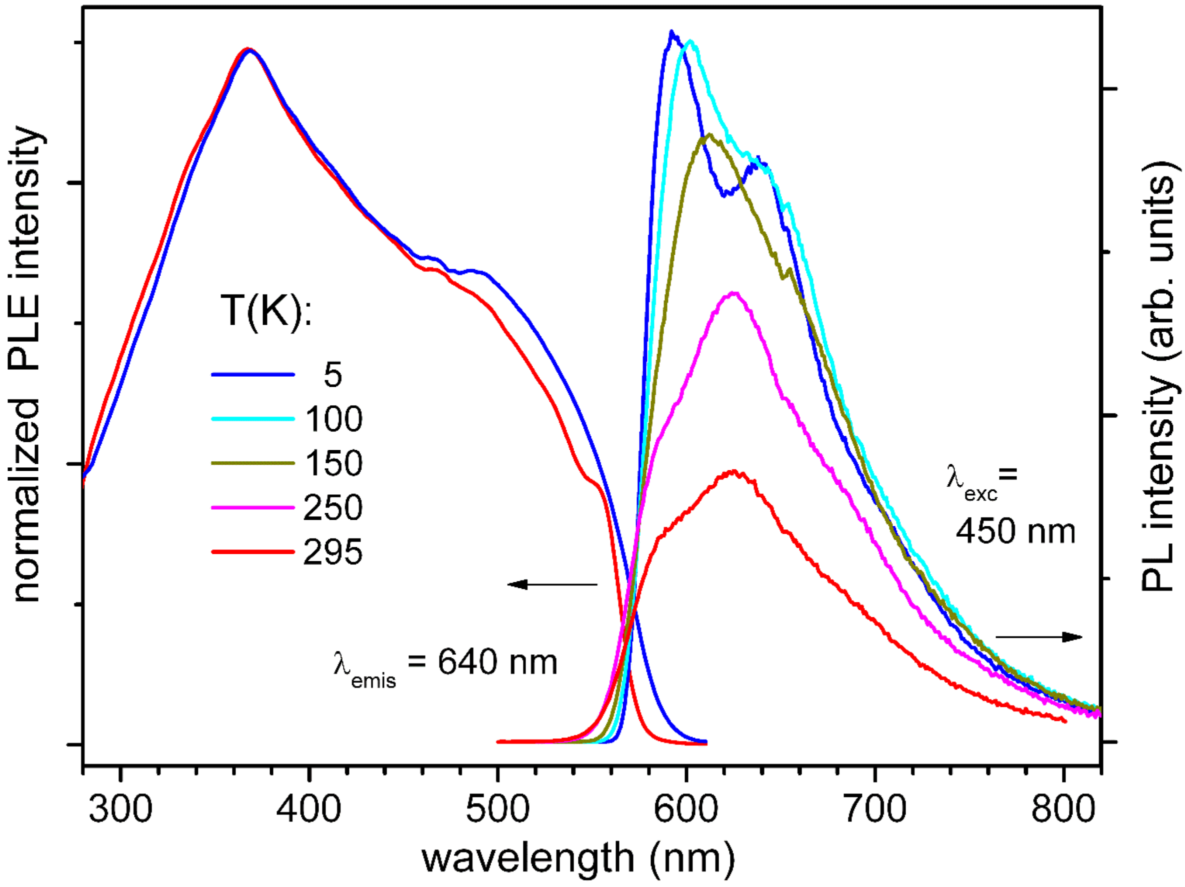

*Figure S8: Emission spectra at absorption maximum 450 nm of solid syn-[{AuCl}(**L1**∩**L2**){Ru(bpy)$_2$}][PF$_6$]$_2$ (**3**) at 5 - 298 K.*

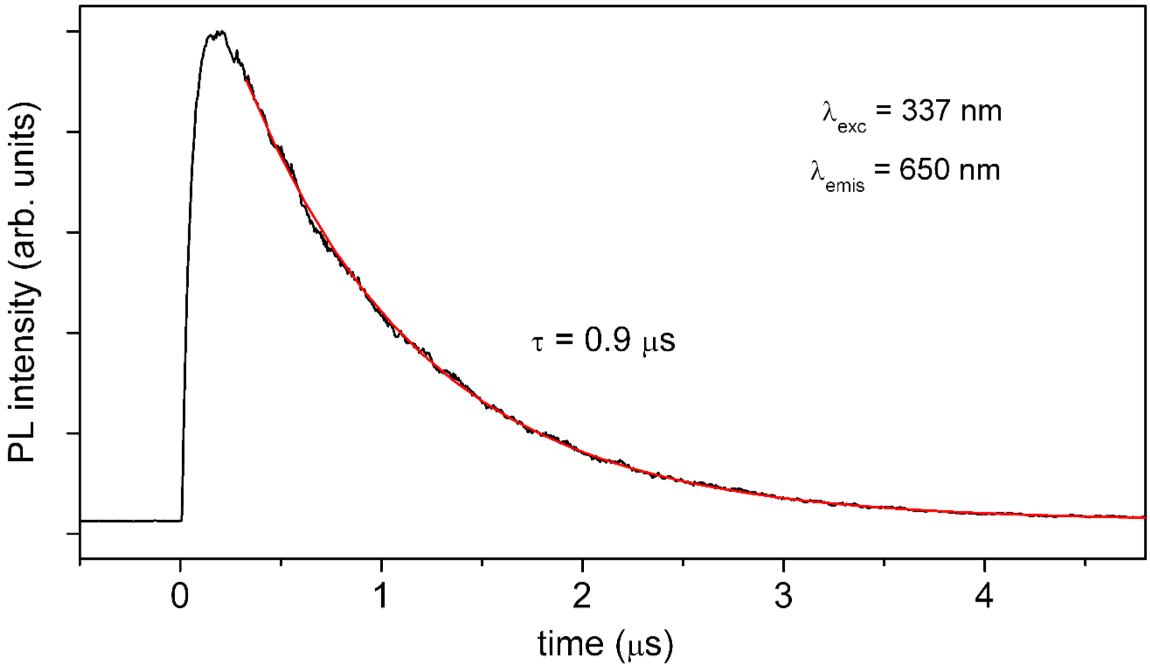

*Figure S9: Decay of syn-[{AuCl}(**L1**∩**L2**){Ru(bpy)$_2$}][PF_6]$_2$ (**3**) in MeCN/EtOH 4:1 (c = 2.04x10^{-6} M) at ambient temperature.*

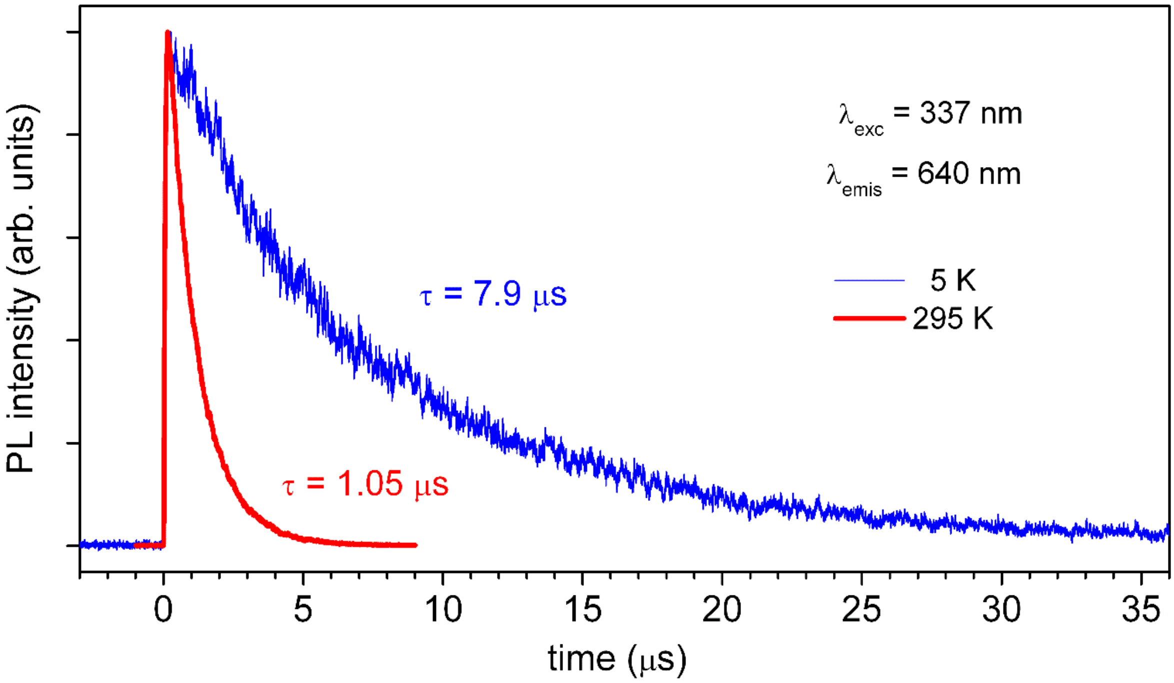

*Figure S10: Decays in solid state of syn-[{AuCl}(**L1**∩**L2**){Ru(bpy)$_2$}][PF_6]$_2$ (**3**) at 5 and 295 K.*

*Table S3: Optical properties of the solid compounds syn-[{AuCl}(**L1**∩**L2**){Ru(bpy)$_2$}][PF$_6$]$_2$ (**3**) and anti-[(**L1**∩**L2**){Ru(bpy)$_2$}][PF$_6$]$_2$ (**5**).*

Compound	Temperature [K]	Φ_P[a]	τ_P [μs][b]
**3**	5	-	7.9
	295	0.18	1.05
**5**	5	-	10.5
	295	0.18	0.49

[a] Quantum yield, uncertainty ± 0.10. [b]Lifetime of phosphorescence.

S4 UV-vis spectrum of catalysis substrates

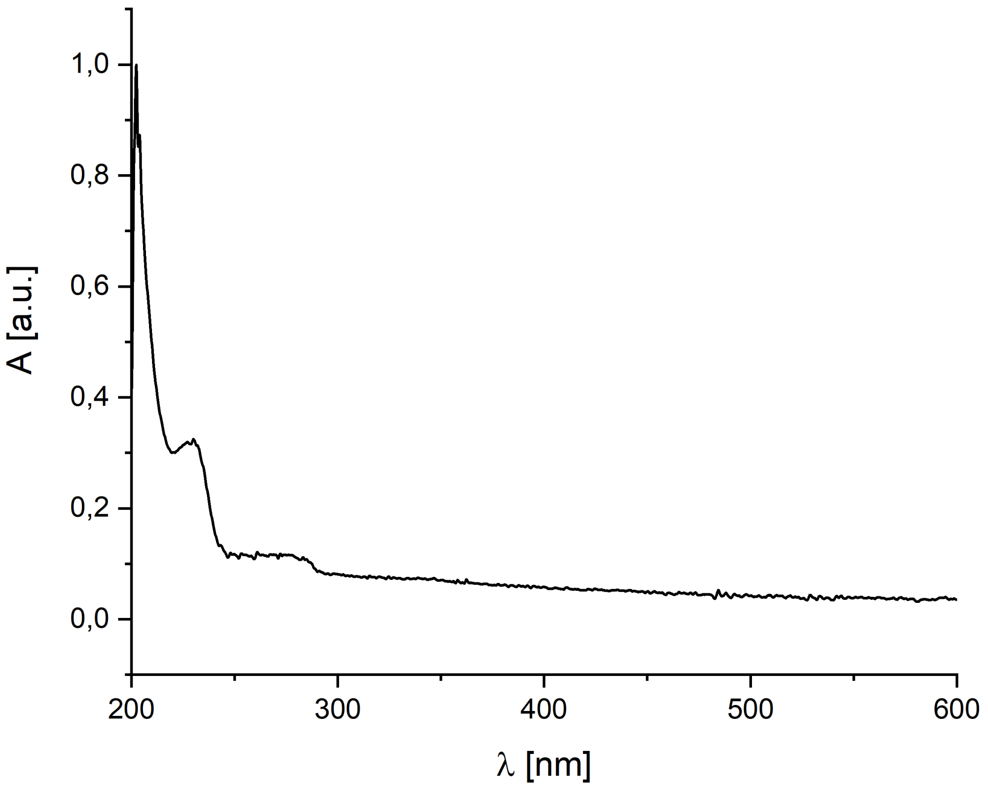

*Figure S11: Absorption spectrum of **7**, **8a** and triphenylphosphate in MeCN/EtOH, 4:1 at 298 K (c(**7**) = c(**8a**) = c(triphenylphosphate) = 1.17*10^{-5} M).S*

S5. Kinetic studies

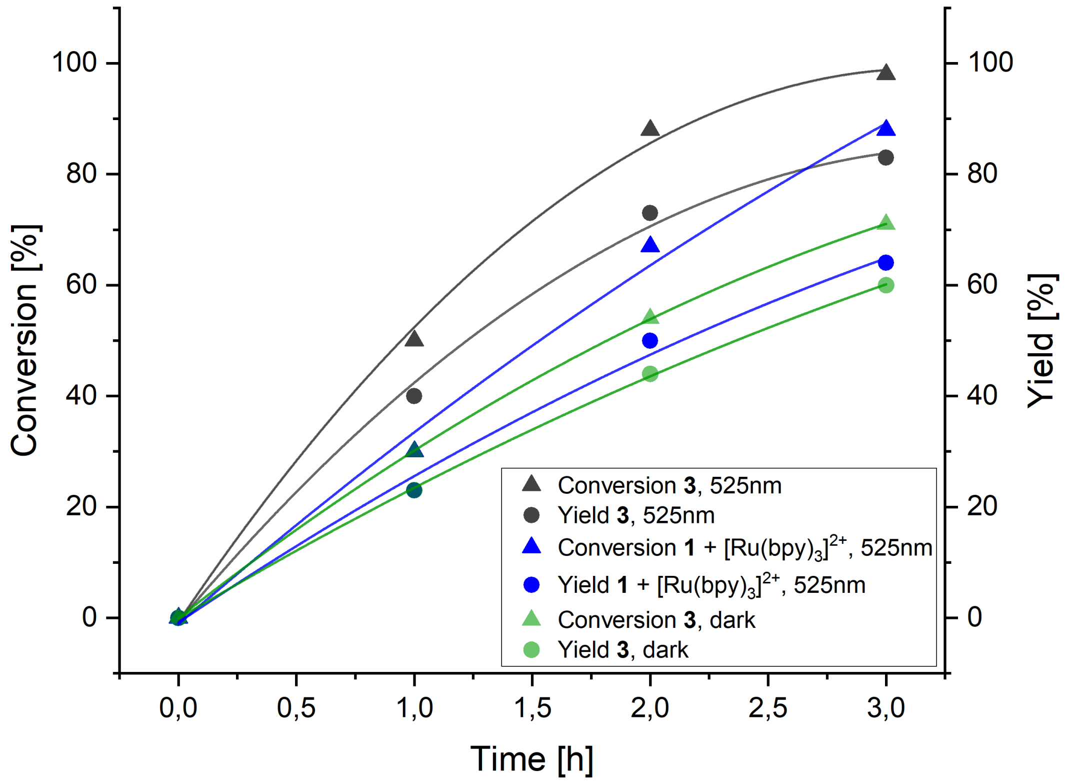

*Figure S12: **7** (0.1 mmol), **8a** (0.15 mmol); after 1 hour another 1.5 equivalents and after 2 hours 1 equivalent of **8a** was added, respectively. Conversions and yields were determined by ^{31}P NMR spectroscopy with triphenylphosphate as internal standard.*

S6. NMR spectra

N-Bipyridinylmaleamic acid

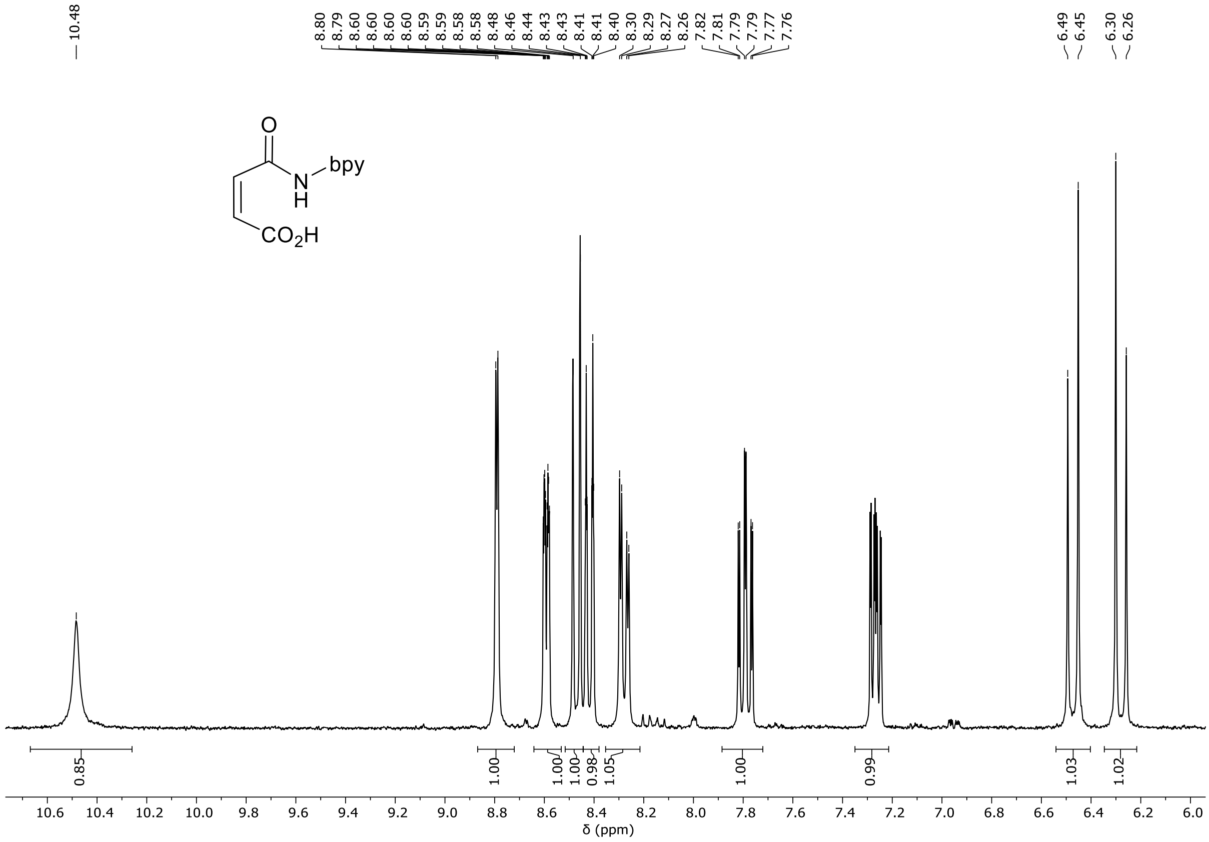

*Figure S13: ¹H NMR spectrum (300 MHz, THF-d_8) of **N-Bipyridinylmaleamic acid**.*

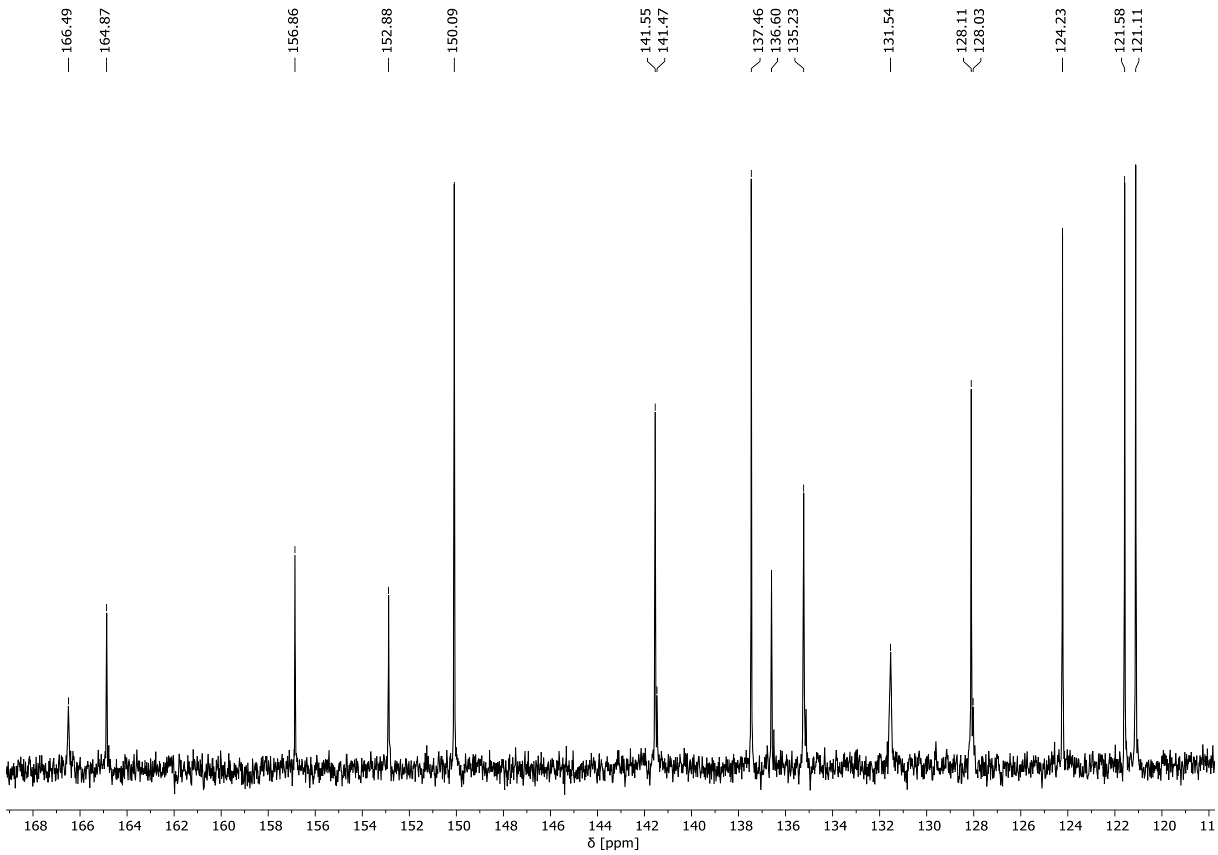

*Figure S14: $^{13}C\{^{1}H\}$ NMR spectrum (75 MHz, THF-d_8) of **N-Bipyridinylmaleamic acid**.*

L2

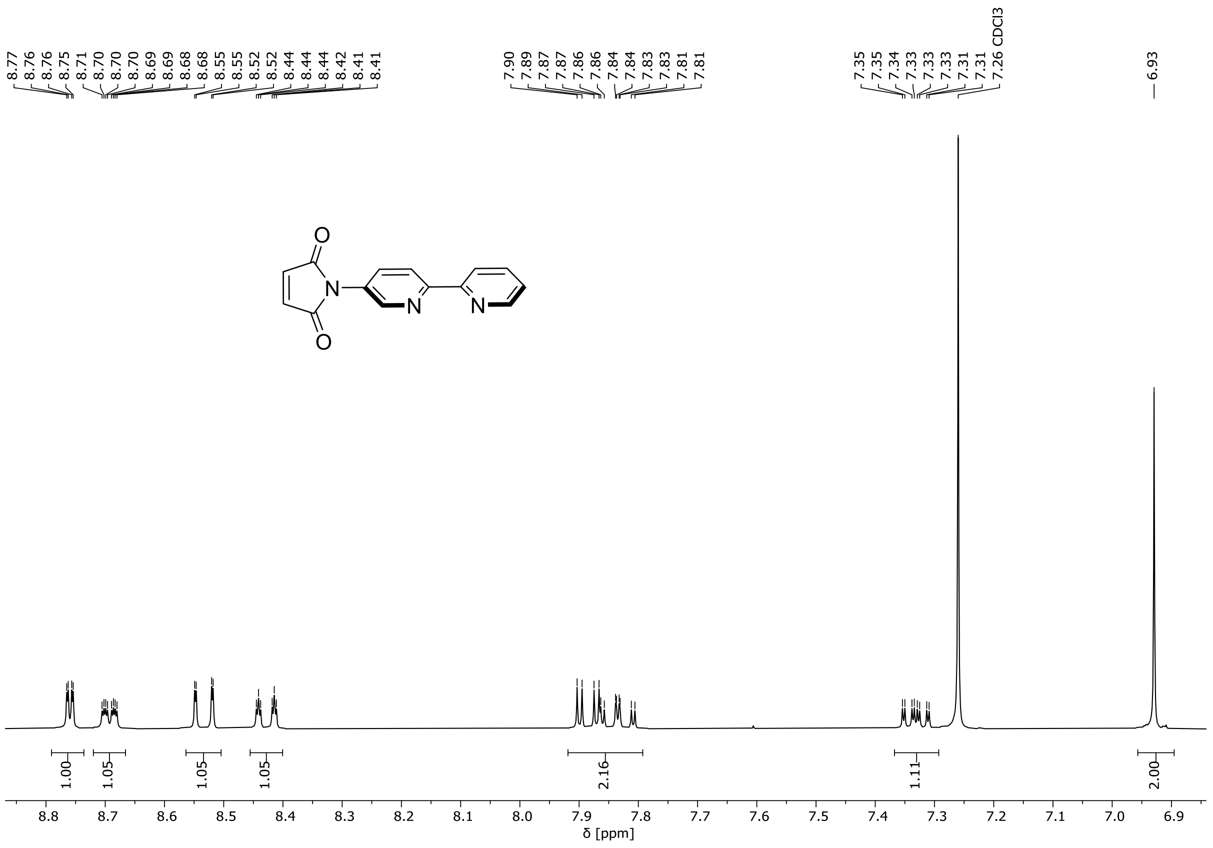

*Figure S15: ^{1}H NMR spectrum (300 MHz, CDCl3) of **L2**.*

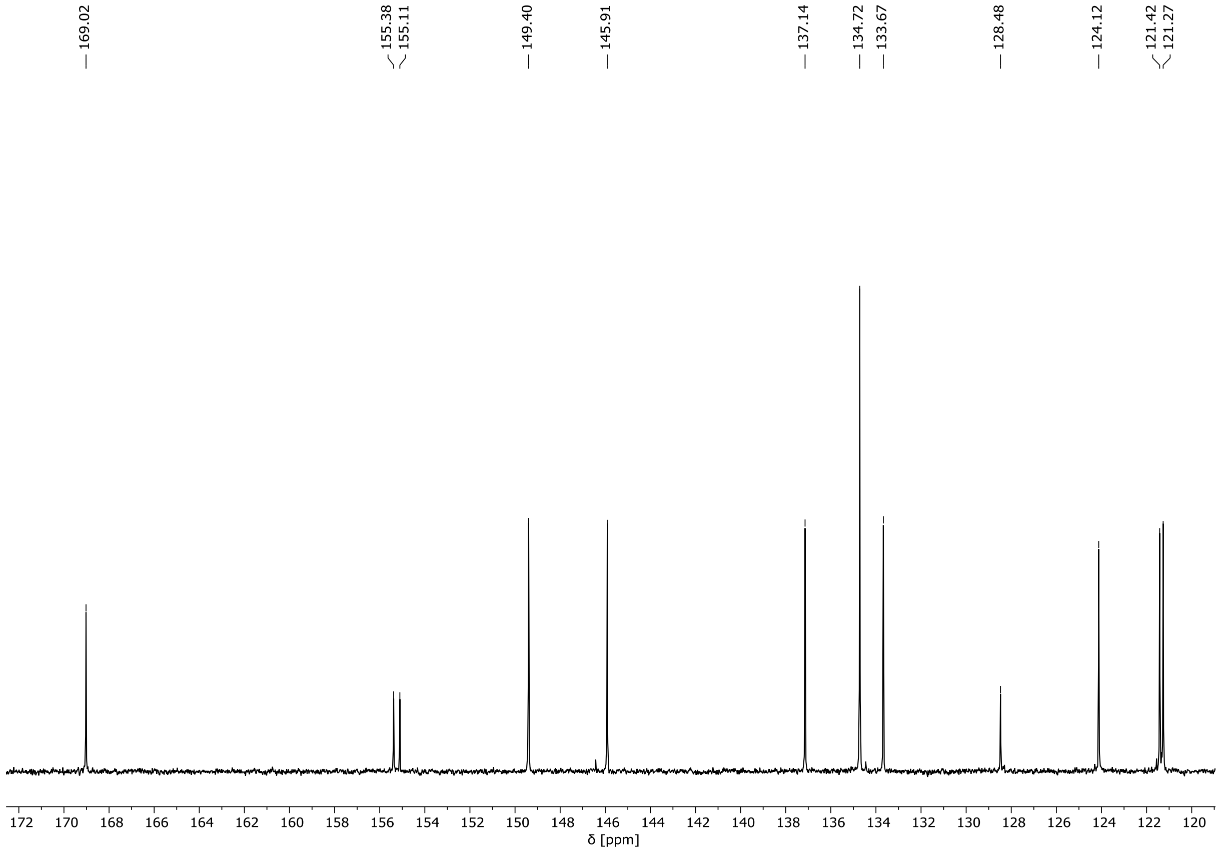

*Figure S16: $^{13}C\{^{1}H\}$ NMR spectrum (75 MHz, CDCl3) of **L2**.*

syn-[{AuCl}(**L2**∩**L1**)] (**2**)

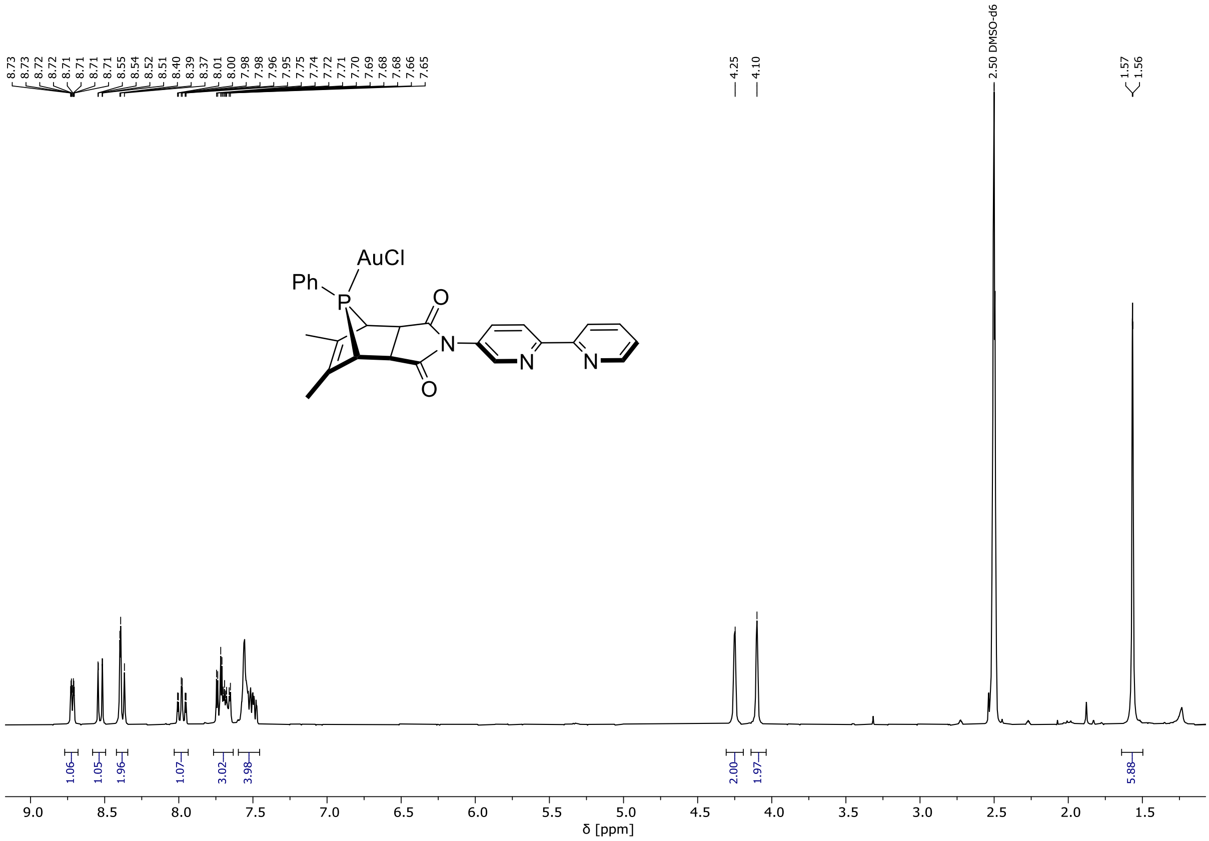

*Figure S17: ¹H NMR spectrum (300 MHz, DMSO-d6) of syn-[{AuCl}(**L2**∩**L1**)] (**2**).*

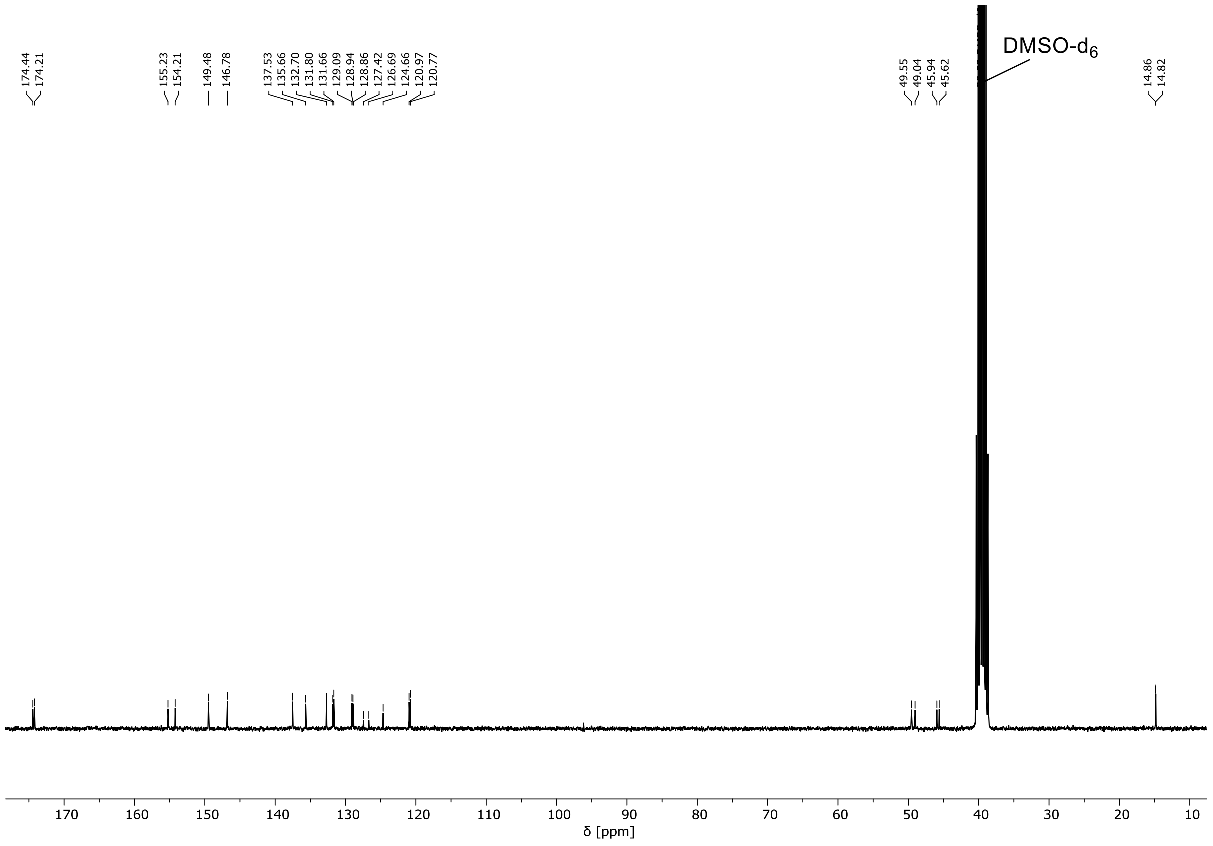

*Figure S18: $^{13}C\{^1H\}$ NMR spectrum (75 MHz, DMSO-d_6) of syn-[{AuCl}(**L2**∩**L1**)] (**2**).*

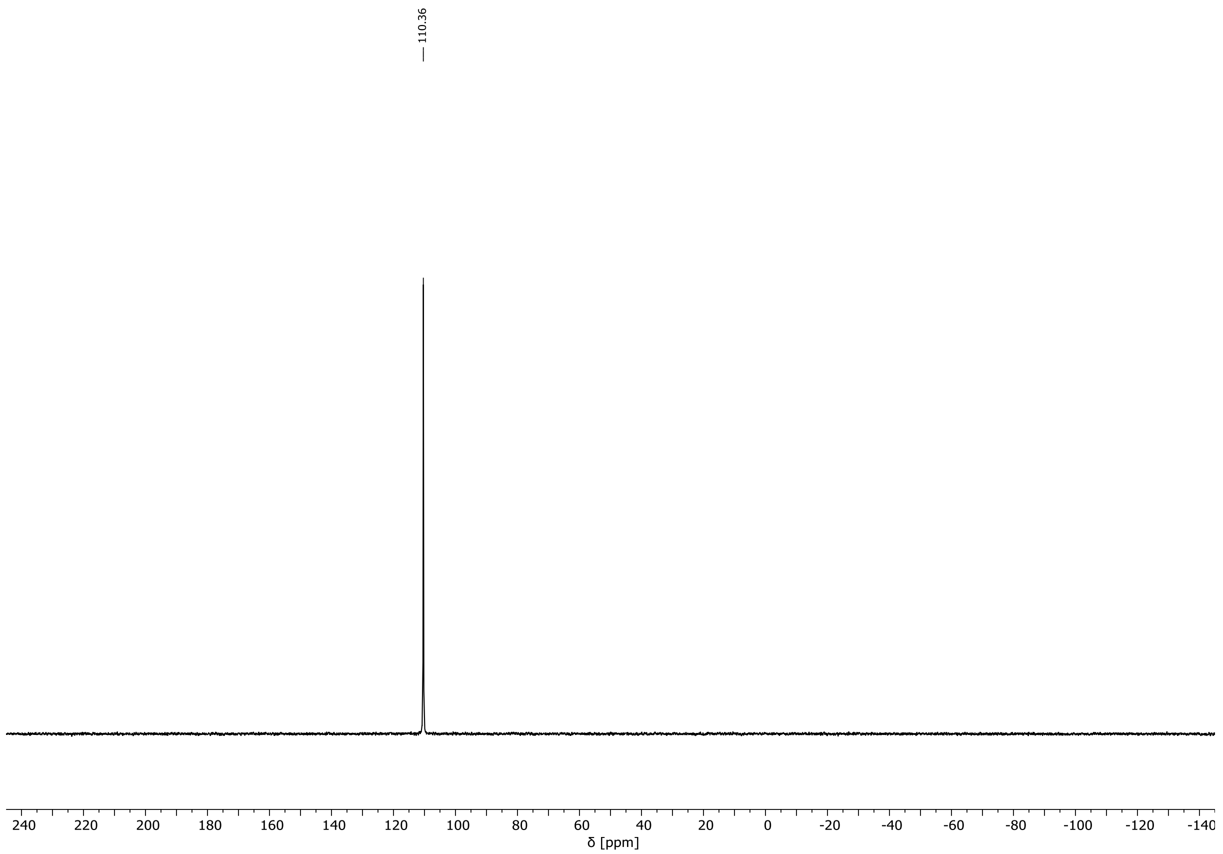

*Figure S19: $^{31}P\{^1H\}$ NMR spectrum (121 MHz, DMSO-d6) of syn-[{AuCl}(**L2**∩**L1**)] (**2**).*

[(**L2**){Ru(bpy)$_2$}][PF$_6$]$_2$ (**4**)

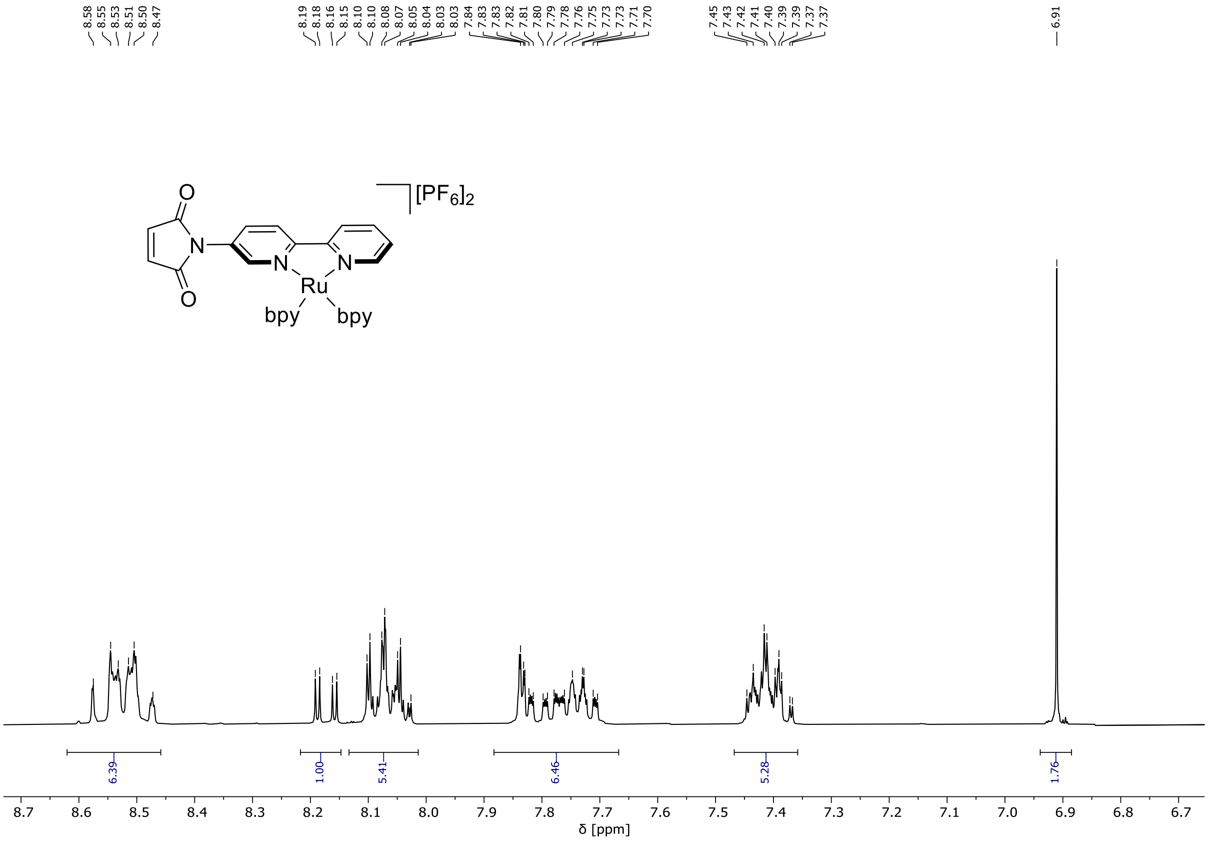

*Figure S20: ^{1}H NMR spectrum (300 MHz, CD_3CN) of [(**L2**){Ru(bpy)$_2$}][PF$_6$]$_2$ (**4**).*

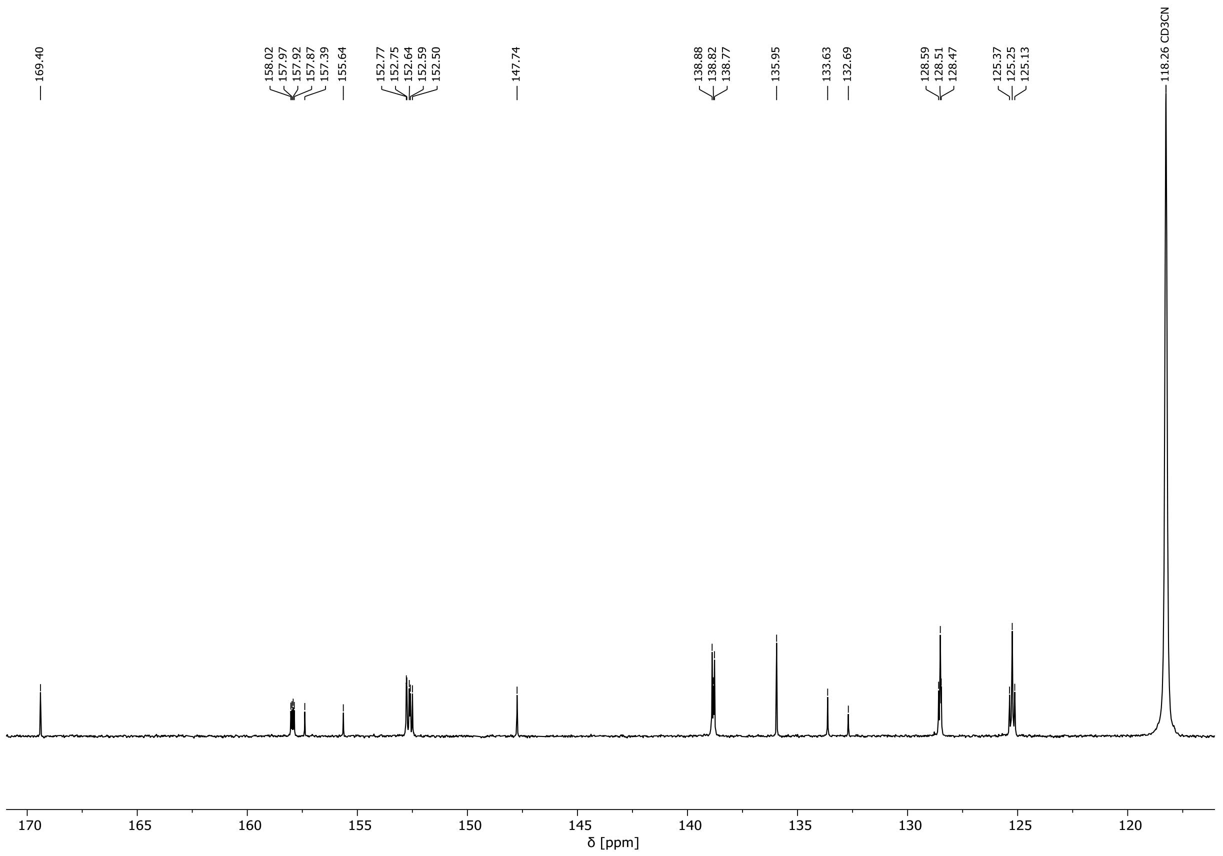

*Figure S21: $^{13}C\{^1H\}$ NMR spectrum (75 MHz, CD_3CN) of [(**L2**){Ru(bpy)$_2$}][PF_6]$_2$ (**4**).*

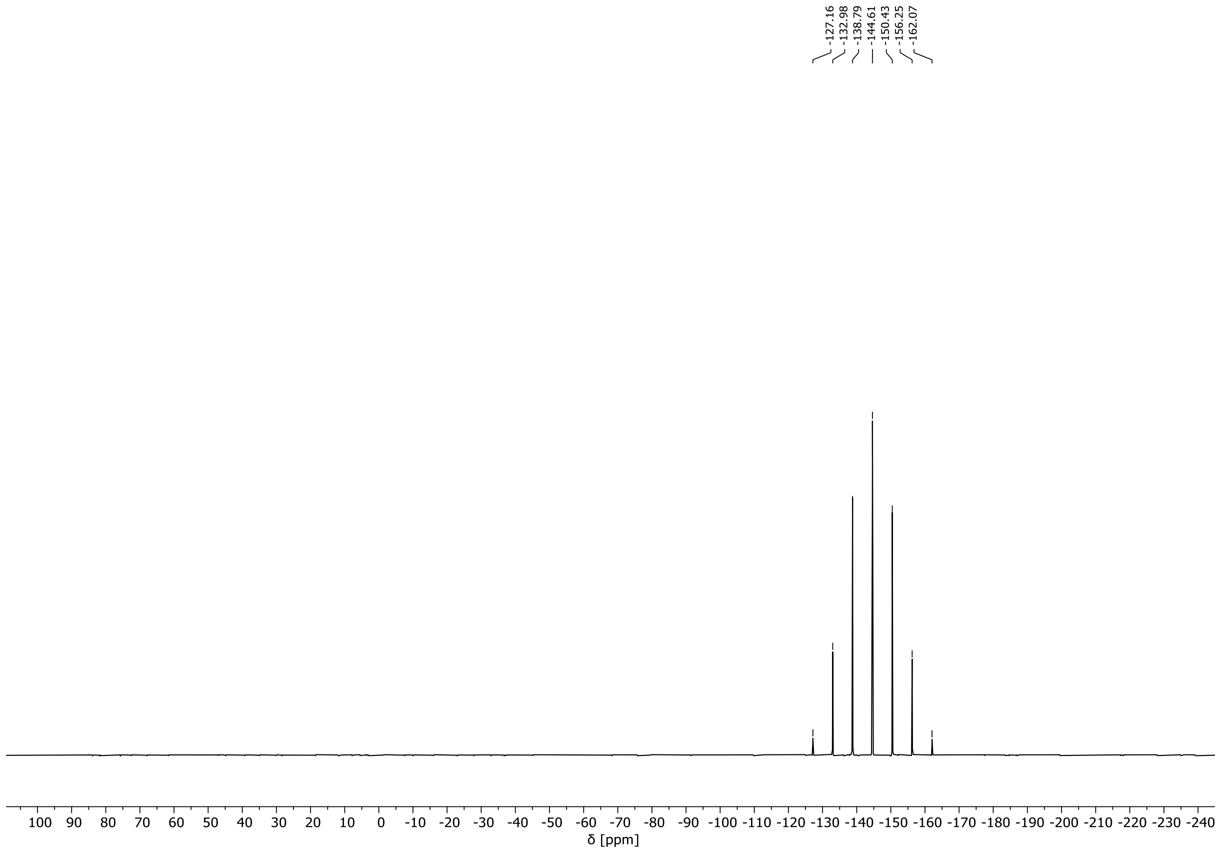

*Figure S22: $^{31}P\{^1H\}$ NMR spectrum (121 MHz, CD3CN) of [(**L2**){Ru(bpy)$_2$}][PF_6]$_2$ (**4**).*

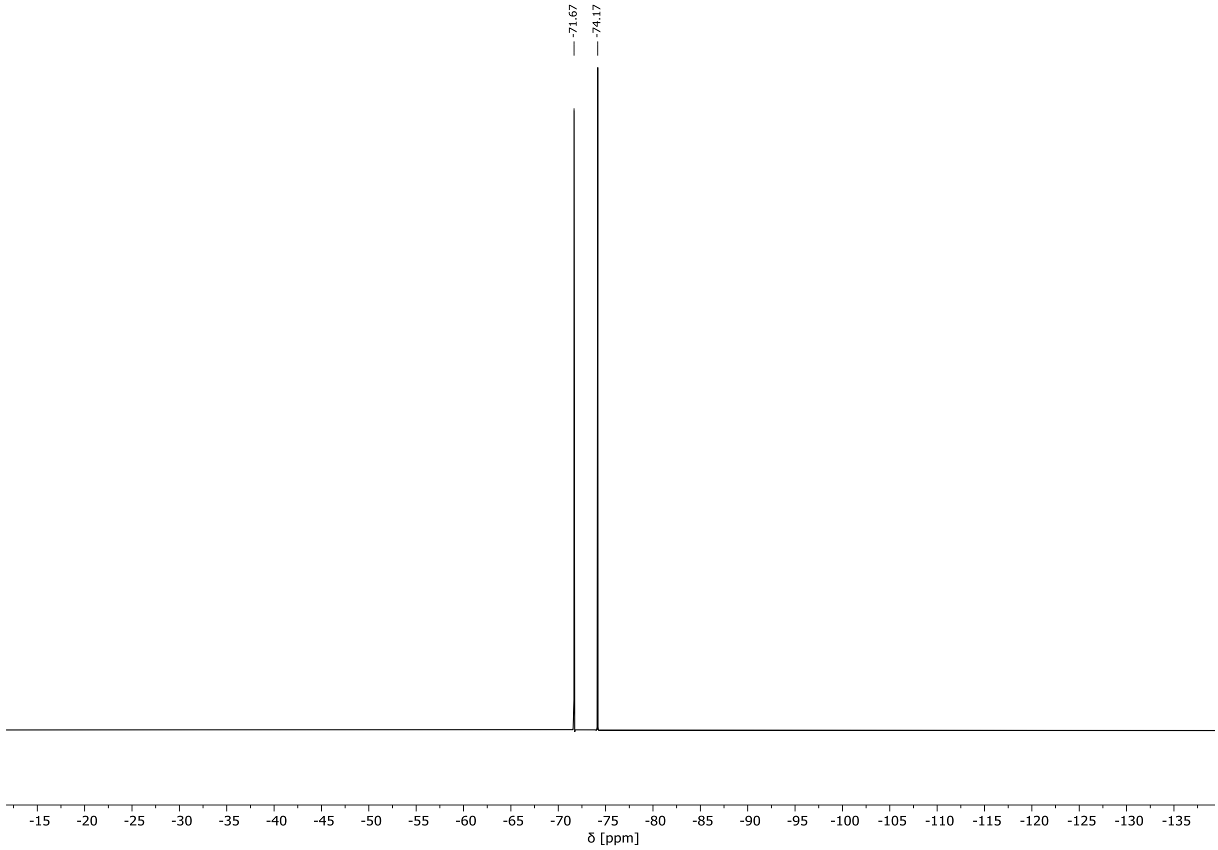

*Figure S23: $^{19}F\{^1H\}$ NMR spectrum (282 MHz, CD3CN) of [(**L2**){Ru(bpy)$_2$}][PF$_6$]$_2$ (**4**).*

anti-[(**L1**∩**L2**){Ru(bpy)$_2$}][PF$_6$]$_2$ (**5**)

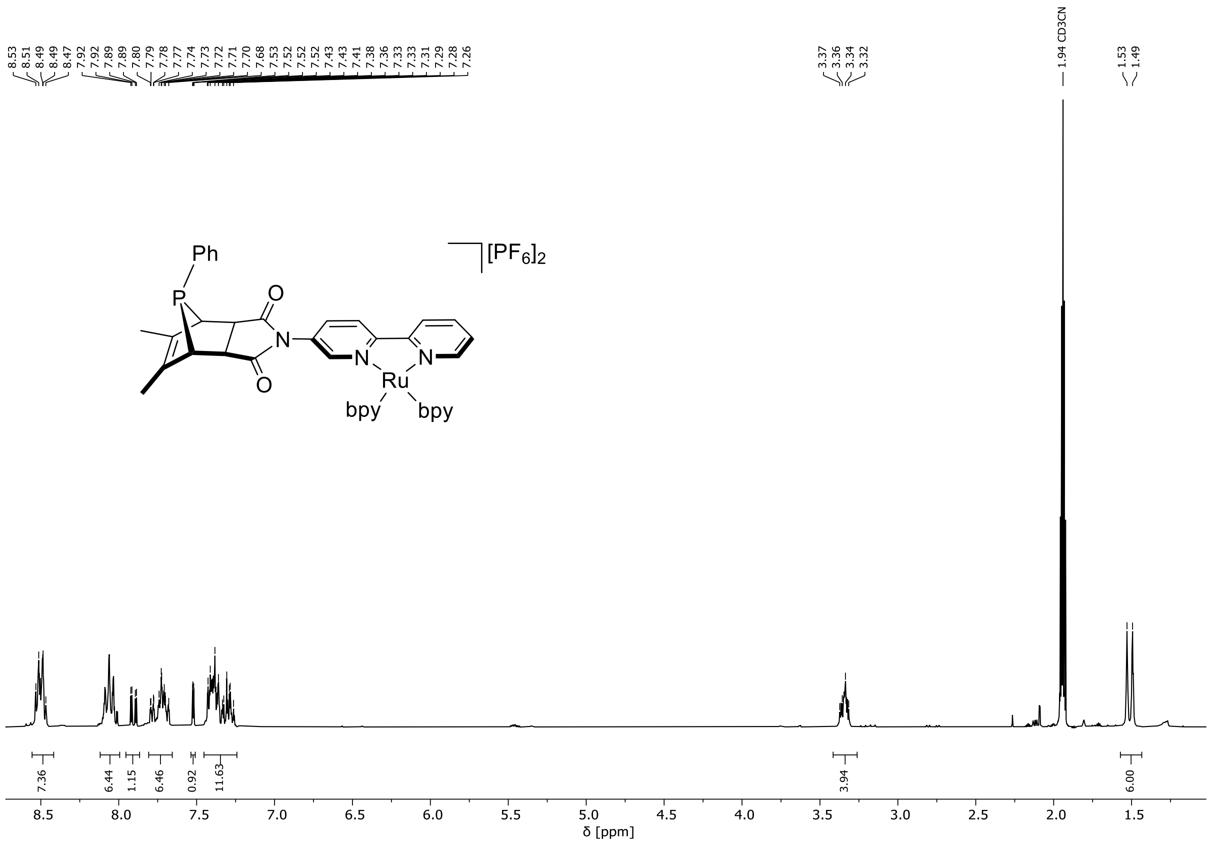

*Figure S24: ^{1}H NMR spectrum (300 MHz, CD_3CN) of anti-[(**L1**∩**L2**){Ru(bpy)$_2$}][PF_6]$_2$ (**5**).*

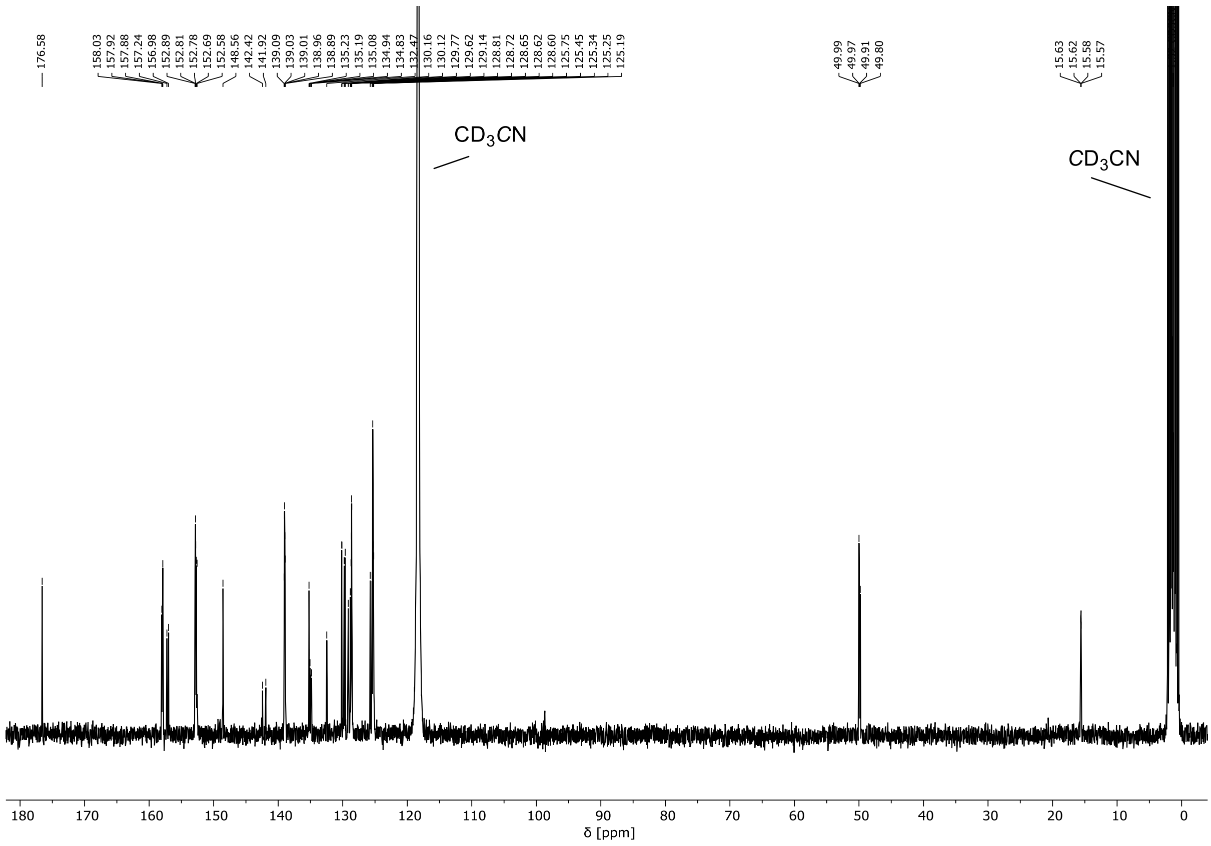

*Figure S25: $^{13}C\{^{1}H\}$ NMR spectrum (101 MHz, CD_3CN) of anti-[(**L1**∩**L2**){Ru(bpy)$_2$}][PF_6]$_2$ (**5**).*

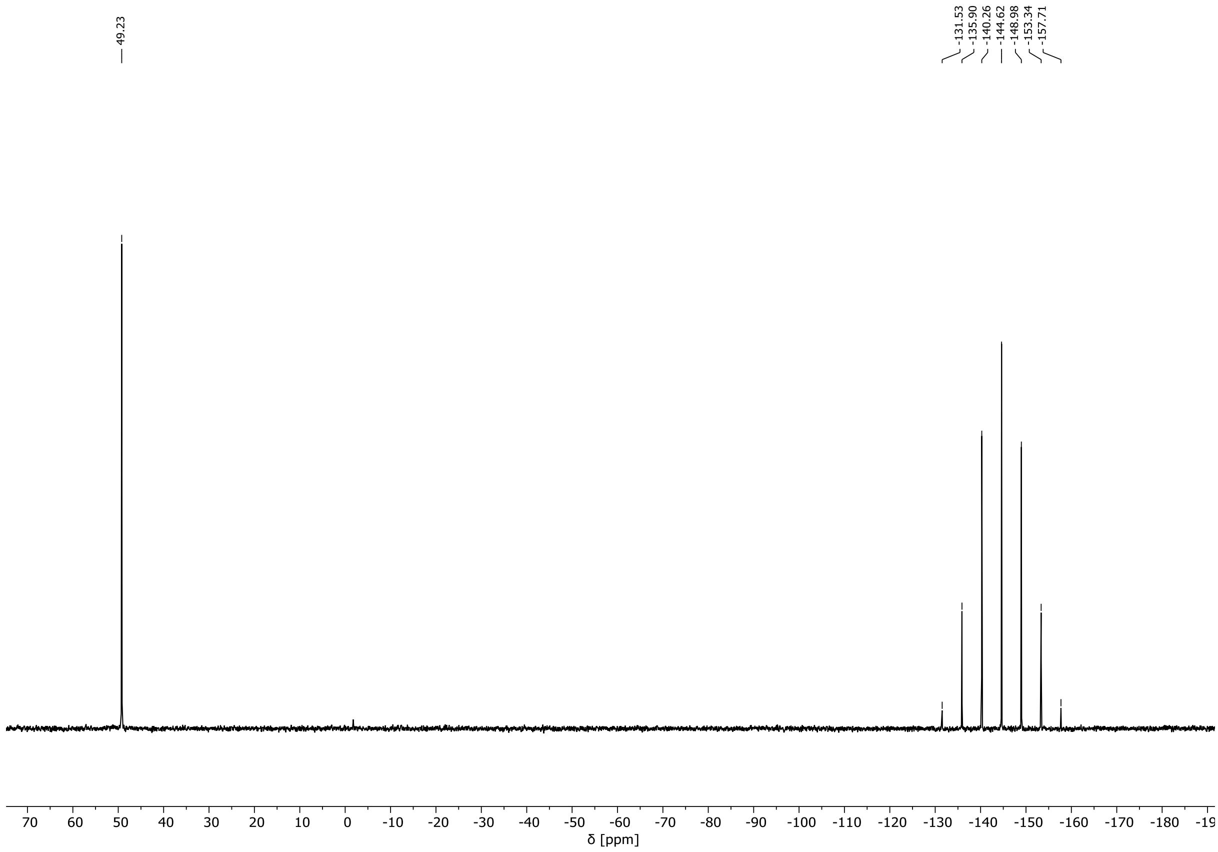

*Figure S26: $^{31}P\{^{1}H\}$ NMR spectrum (162 MHz, CD3CN) of anti-[(**L1**∩**L2**){Ru(bpy)$_2$}][PF$_6$]$_2$ (**5**).*

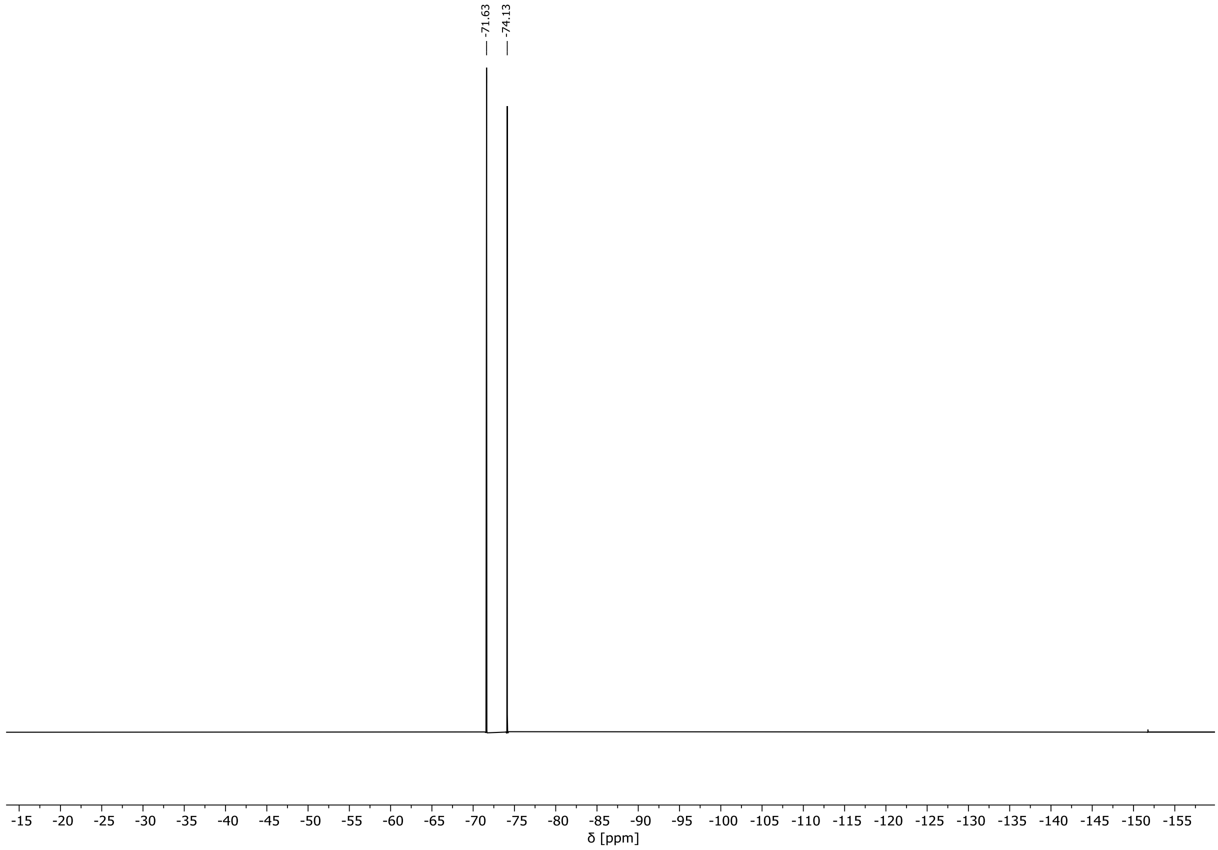

*Figure S27: $^{19}F\{^{1}H\}$ NMR spectrum (282 MHz, CD_3CN) of anti-[(**L1**∩**L2**){Ru(bpy)$_2$}][PF_6]$_2$ (**5**).*

syn-[{AuCl}(**L1**∩**L2**){Ru(bpy)$_2$}][PF_6]$_2$ (**3**)

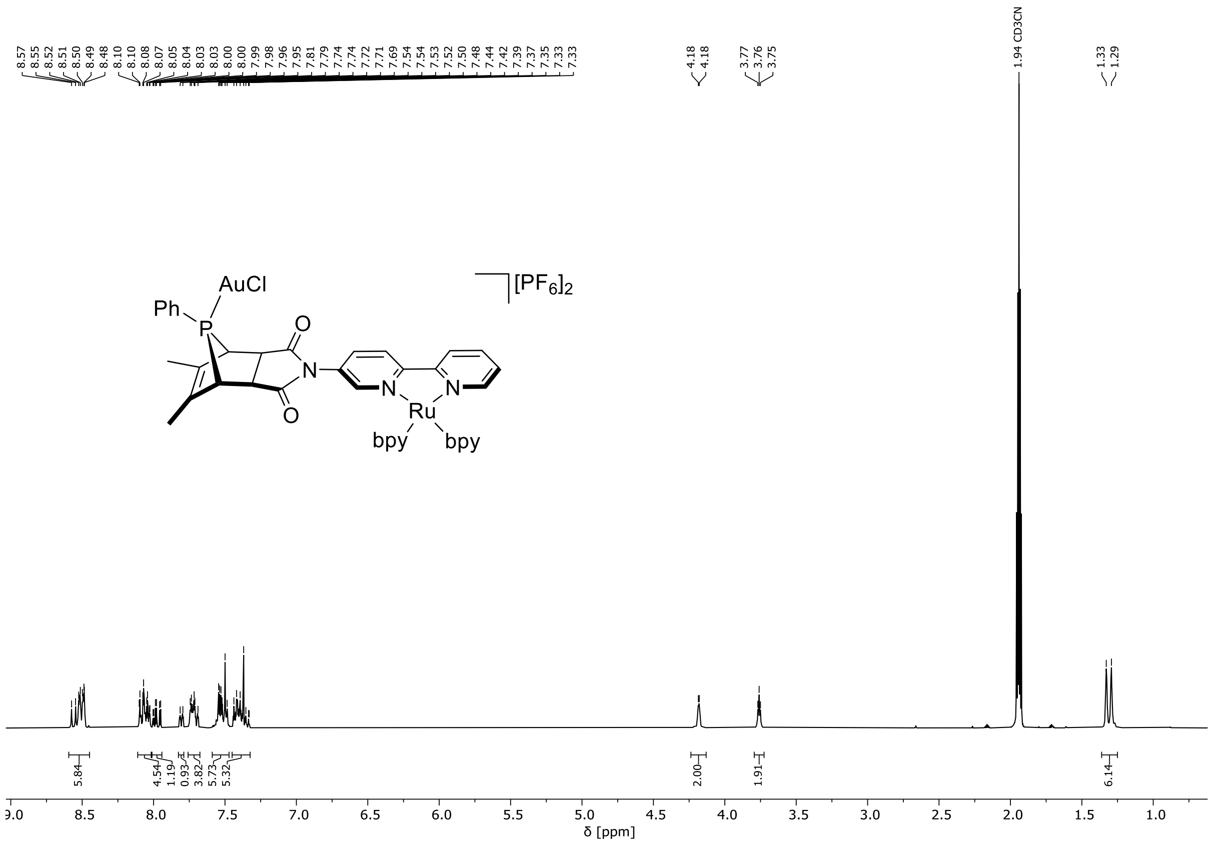

*Figure S28: ^{1}H NMR spectrum (300 MHz, CD_3CN) of syn-[{AuCl}(**L1**∩**L2**){Ru(bpy)$_2$}][PF_6]$_2$ (**3**).*

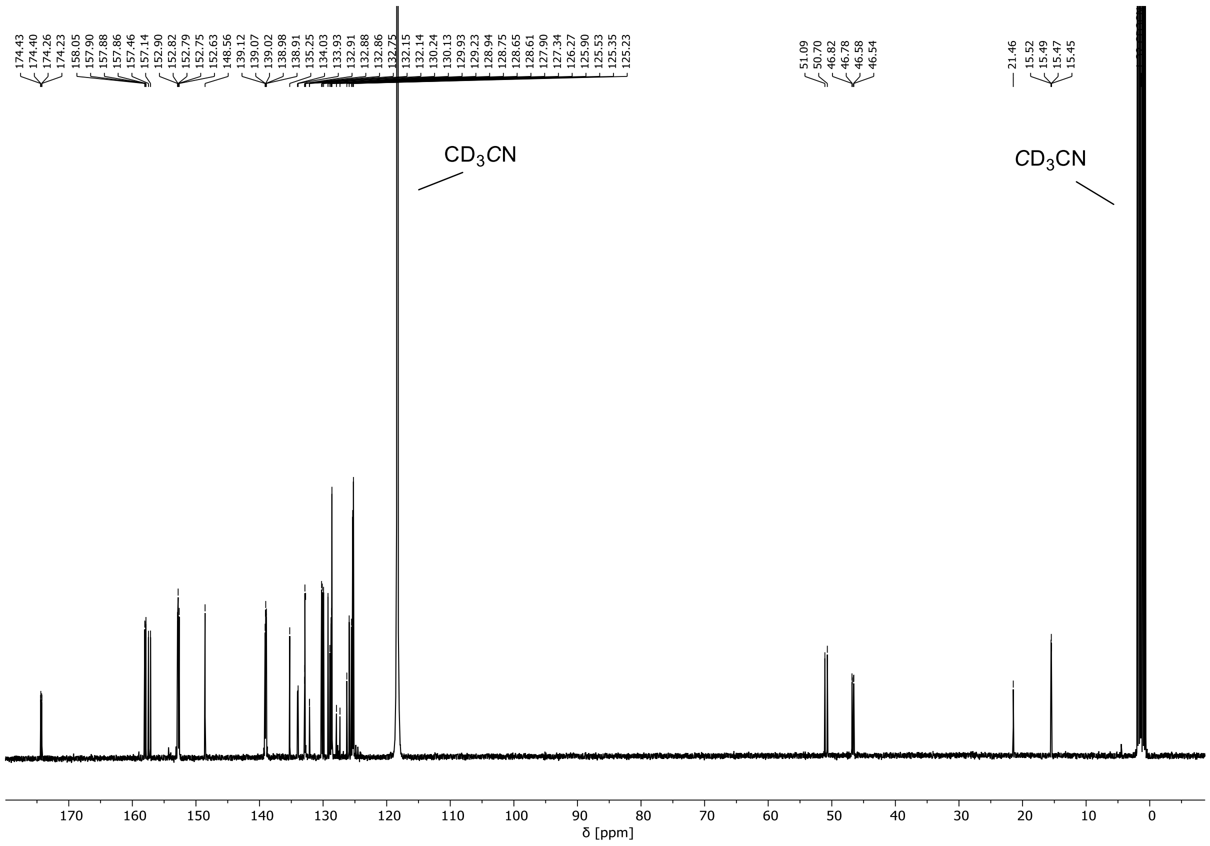

*Figure S29: $^{13}C\{^{1}H\}$ NMR spectrum (101 MHz, CD_3CN) of syn-[{AuCl}(**L1**∩**L2**){Ru(bpy)$_2$}][PF_6]$_2$ (**3**).*

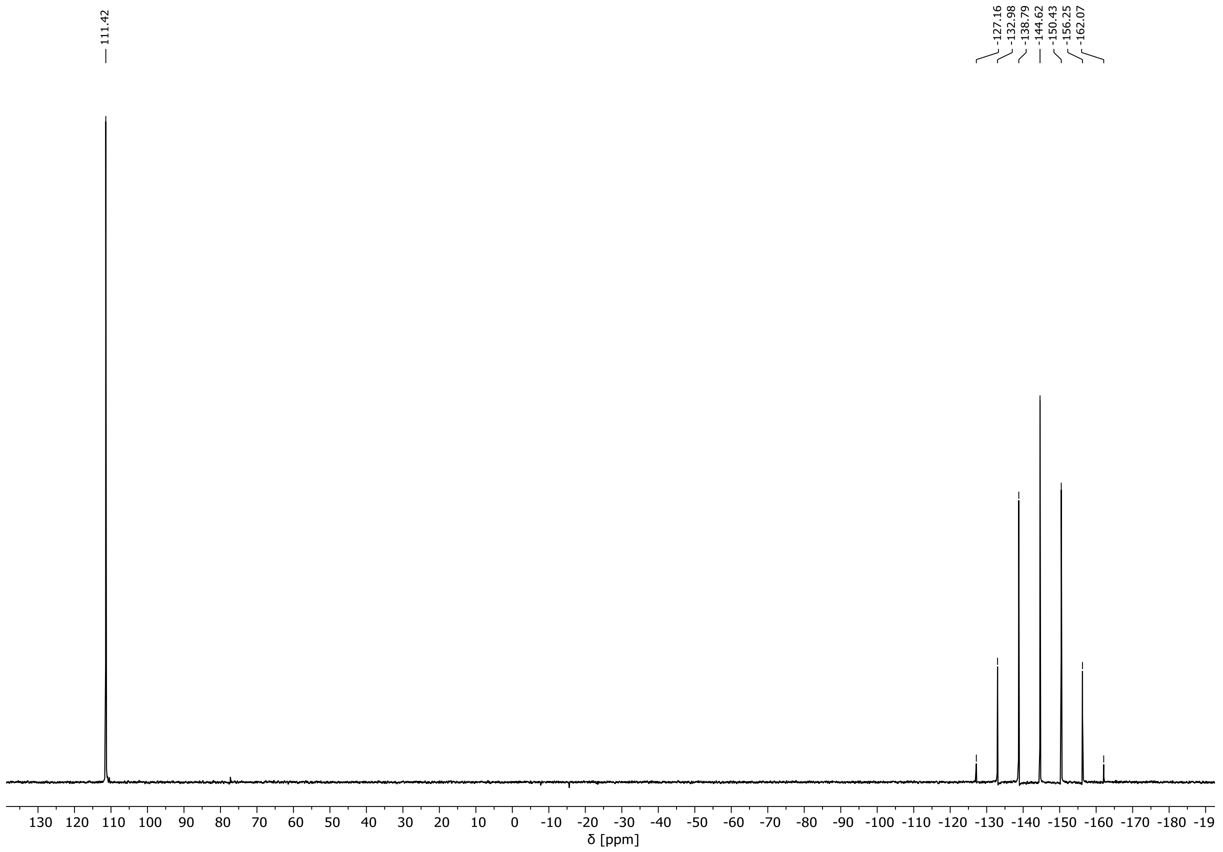

*Figure S30: $^{31}P\{^1H\}$ NMR spectrum (121 MHz, CD3CN) of syn-[{AuCl}(**L1**∩**L2**){Ru(bpy)$_2$}][PF$_6$]$_2$ (**3**).*

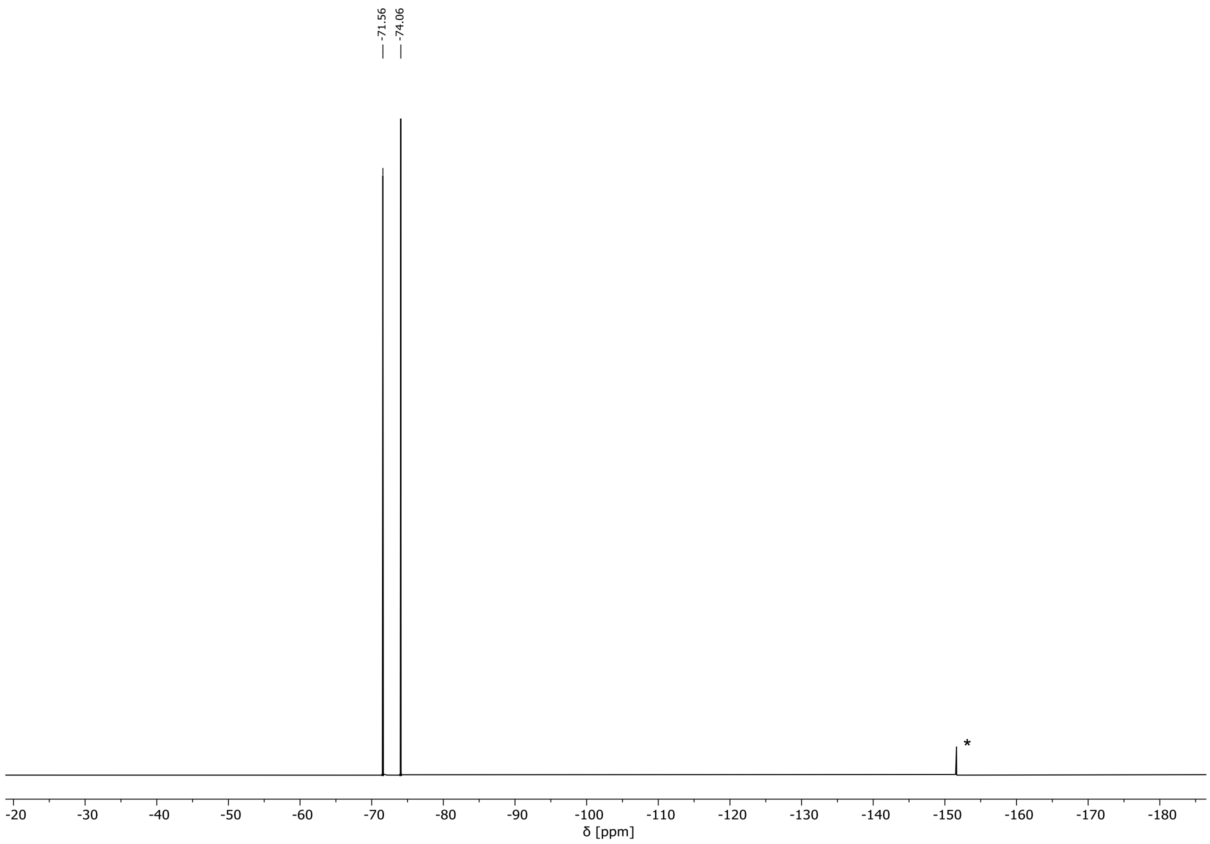

*Figure S31: $^{19}F\{^{1}H\}$ NMR spectrum (282 MHz, CD_3CN) of syn-[{AuCl}(**L1**∩**L2**){Ru(bpy)$_2$}][PF_6]$_2$ (**3**). * = BF_4.*

anti-[{AuCl}(**L1**∩**L2**){Ru(bpy)$_2$}][PF_6]$_2$ (**6**)

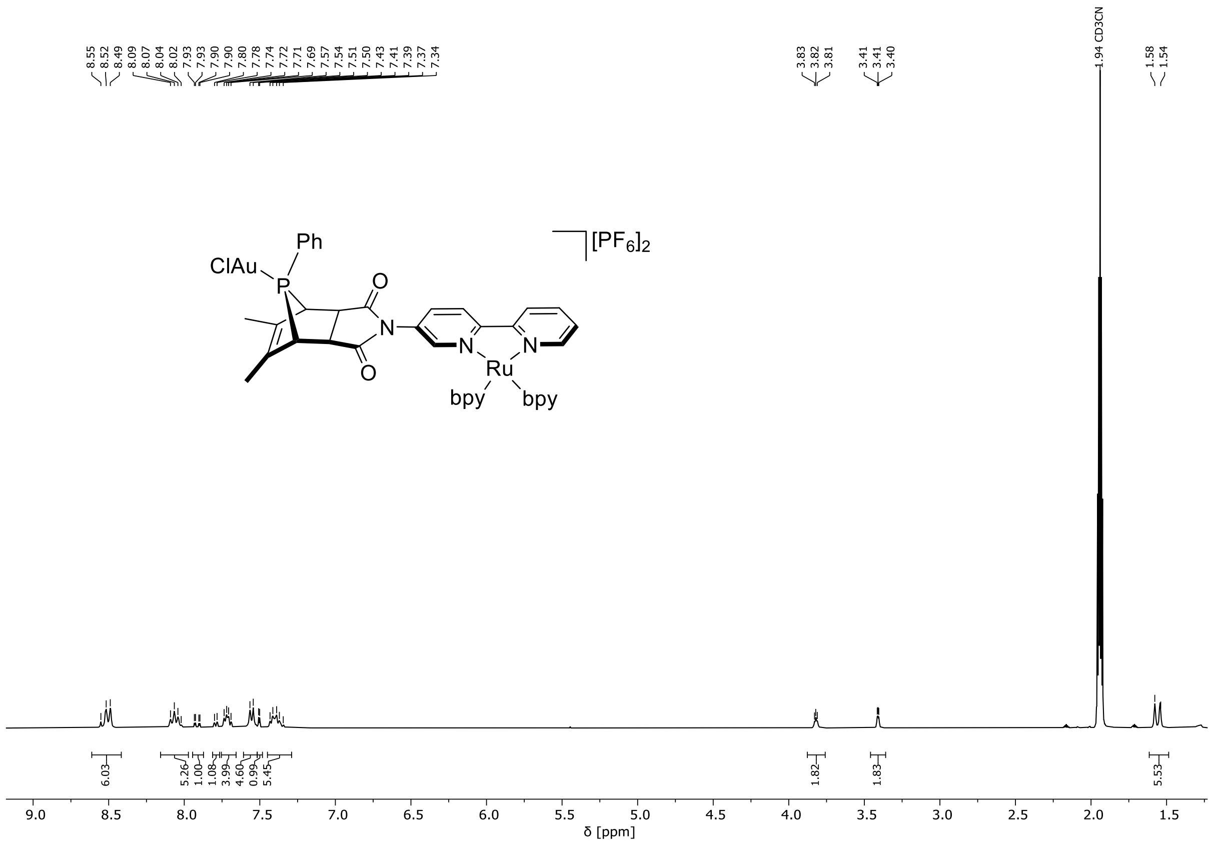

*Figure S32: ^{1}H NMR spectrum (300 MHz, CD3CN) of anti-[{AuCl}(**L1**∩**L2**){Ru(bpy)$_2$}][PF$_6$]$_2$ (**6**).*

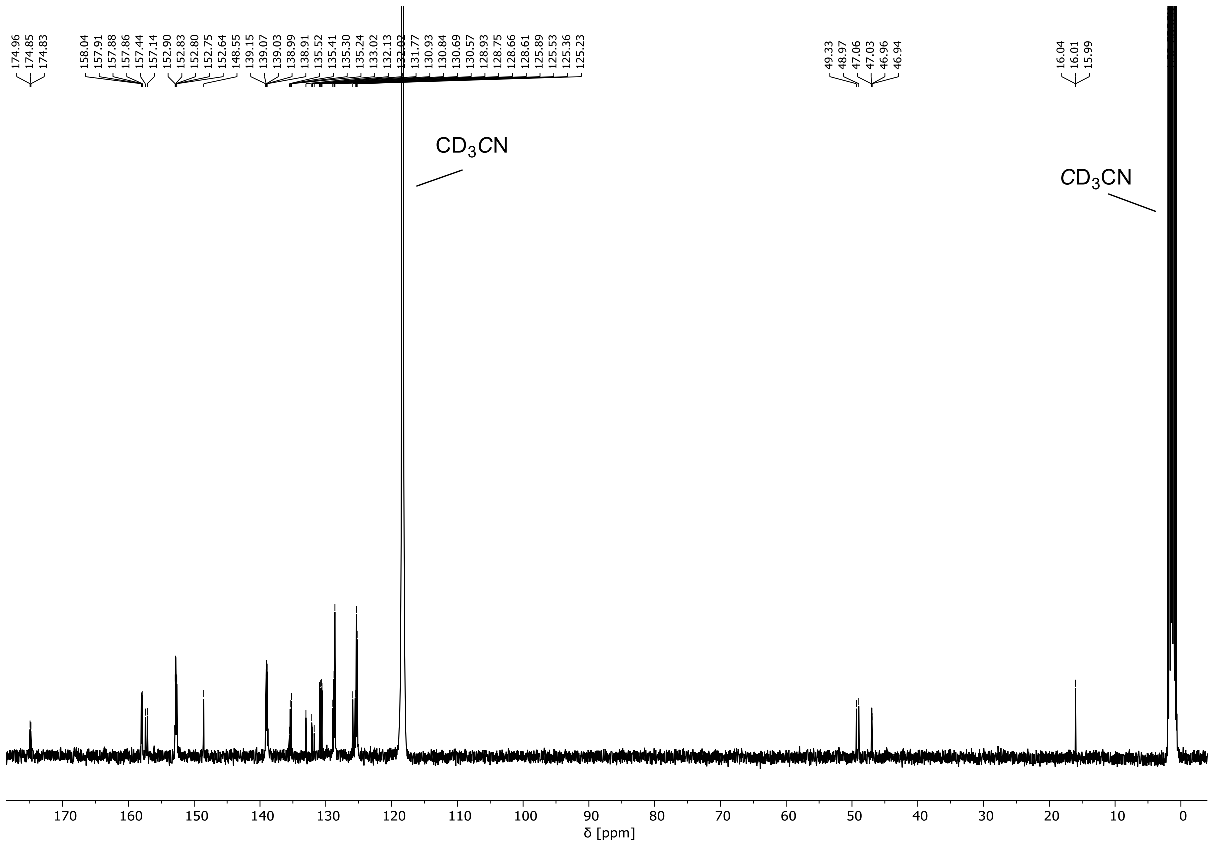

*Figure S33: $^{13}C\{^{1}H\}$ NMR spectrum (101 MHz, CD_3CN) of anti-[{AuCl}(**L1**∩**L2**){Ru(bpy)$_2$}][PF$_6$]$_2$ (**6**).*

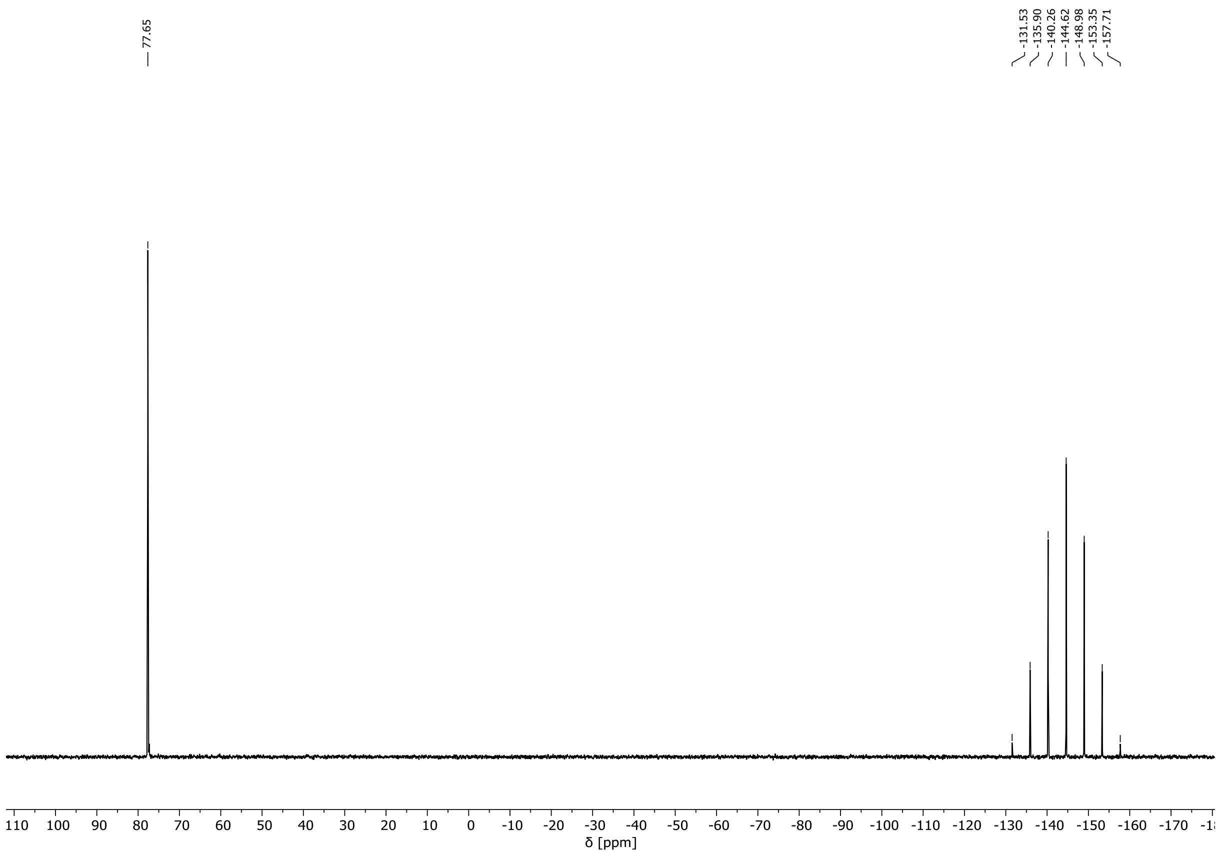

*Figure S34: $^{31}P\{^1H\}$ NMR spectrum (121 MHz, CD_3CN) of anti-[{AuCl}(**L1**∩**L2**){Ru(bpy)$_2$}][PF_6]$_2$ (**6**).*

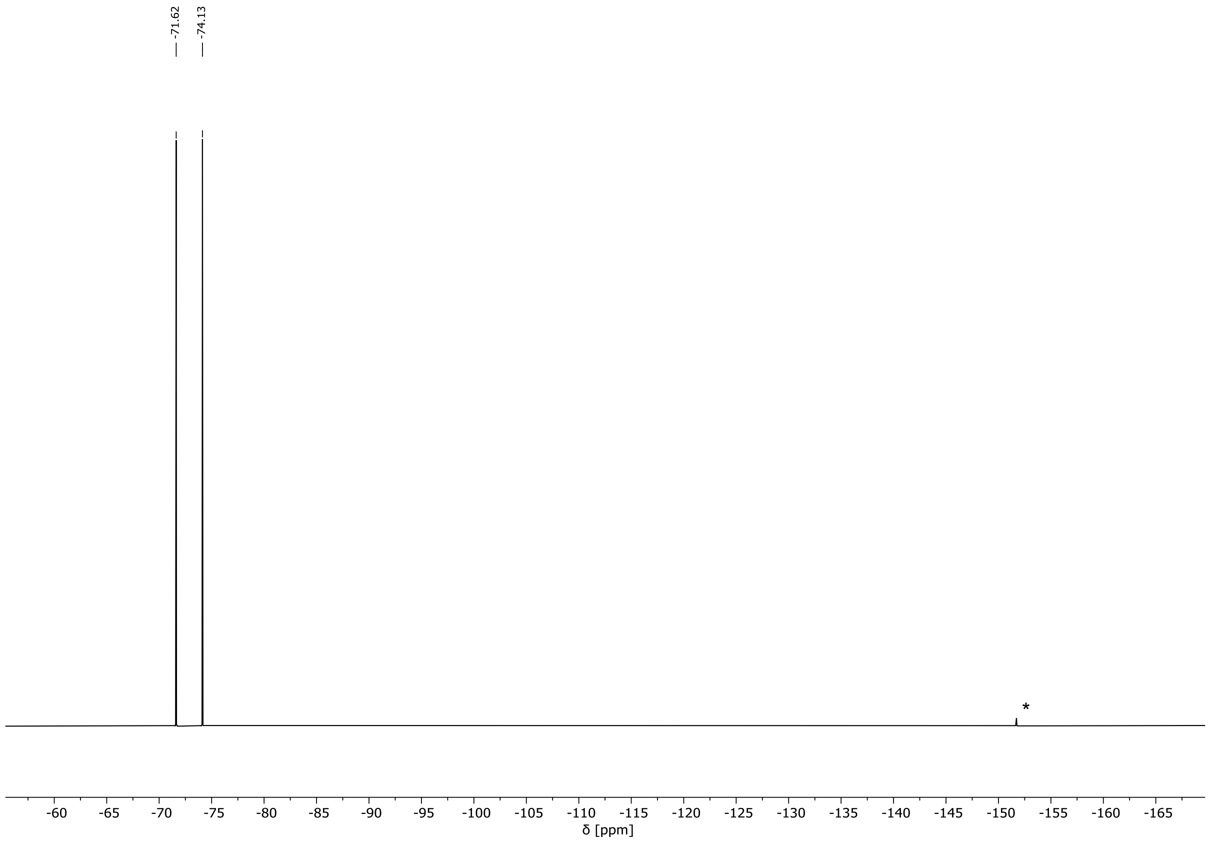

*Figure S35: $^{19}F\{^1H\}$ NMR (282 MHz, CD_3CN) of anti-[{AuCl}(**L1**∩**L2**){Ru(bpy)$_2$}][PF_6]$_2$ (**6**). * = BF_4.*

S7. Coordinates of calculated compounds

Coordinates of the minimum structures:

Syn-endo

48

Coordinates from ORCA-job orca

Au -0.31370749265678 4.51275133492338 17.03000251399760

P -0.79324842820586 4.10899915040696 14.87202905588136

Cl 0.21784144292820 4.92374646002313 19.22899368221155

O 3.45844980044506 3.03183823252549 12.82836843807122

C 5.86809593891238 5.16486132540318 10.12728752310989

O 1.70441774674136 7.26961523152407 12.21158963705305

N 2.83229406626516 5.22185979657873 12.29665954098658

C 5.03605064842742 5.00349801602994 11.24461428896528

H 5.42375453674588 4.56219598239061 12.17165200761035

C 3.68814891400920 5.40097887796482 11.16694588841322

C 4.02009530284715 6.12420789753916 8.87433386014237

C 5.36601326684875 5.72716439513867 8.94211885640918

C 0.51449667816146 3.18258703691143 13.87441678584346

H 0.73290571219901 2.14529777974111 14.18120981546867

C 3.17470605985207 5.95870957406586 9.98060959085728

C 2.78175388198700 4.01923388107107 13.04233696188040

C 1.13048668285261 5.59950205222202 13.91694510995198

H 1.26846294994452 6.25511418832975 14.79992445396640

C -0.39519239729675 5.42111735651951 13.58483293524507

H -0.96542244522900 6.36530024337059 13.62737392350368

C 1.89009393776872 6.18429160471824 12.72851869179182

C -0.48430907363466 4.63209327813431 12.27645418247636

C 1.70595249453540 4.17473639291825 14.11112796766071

H 2.15382435048661 4.01321466439650 15.11227392028320

C 0.02124010790665 3.37501212806904 12.44326281830447

C	-2.42068176886180	3.36188832650963	14.54444690131136
C	0.20722833587359	2.29455455869843	11.42675870425956
H	1.28023467871879	2.02112877337541	11.33580464265549
H	-0.16244343839139	2.59094522713391	10.42688315475890
H	-0.33136018811841	1.37231894669879	11.73442209713797
C	-0.96099205795811	5.29374671312106	11.02167041561339
H	-2.01841859427755	5.62611414062734	11.11328735071209
H	-0.89054204999647	4.62375698882403	10.14399941029698
H	-0.36758248700070	6.20985898927732	10.81498256041324
C	-3.31016176547721	3.84012183017330	13.56074369903318
H	-3.04070299591510	4.70779795547908	12.94369701408653
C	-4.55120850026942	3.21158400585248	13.37555432776225
H	-5.24548342293846	3.59220119829782	12.61119012346459
C	-2.79019363276126	2.26216847079158	15.34989729231321
H	-2.11039673568047	1.91203647410710	16.14339587127674
C	-4.90695686016638	2.10335441140831	14.16256394072078
H	-5.88074149053669	1.61275108896480	14.01317560322705
C	-4.02590213593403	1.62983123910480	15.15018640352003
H	-4.30683117363965	0.77065252617370	15.77757251355823
H	3.61928525650696	6.56581701751575	7.94946800346627
H	6.92088683747337	4.85041162152962	10.18779825926030
H	2.12328746083619	6.26758904969941	9.93185011277446
H	6.02452204567259	5.85622356572037	8.06994914829180

Syn-exo

48

Coordinates from ORCA-job orca

Au	0.13479191361061	5.94732938691370	16.28513612586972
P	-0.36408959073550	4.84376551878409	14.40032568562078
Cl	0.57814731264325	7.02468725200555	18.27114592370117
O	3.61861244136700	4.78902028874407	14.74247371533501
O	1.07089609635508	8.38234764838031	13.31048205212318
N	2.59691201636987	6.82252011288854	14.17302798271384
C	0.89290789781128	3.78108366607018	13.46818327427670
H	1.36845229850660	2.95862812867203	14.03075045667326
C	2.82445647326421	5.42905020304464	14.08166090549266
C	1.06446409809914	6.06784476455855	12.50679144379597
C	-0.43179423123214	5.66865558318385	12.69970220245003
H	-1.12824929167607	6.51736846637899	12.58275011921594
C	1.52905170479415	7.25797115052416	13.34943086071159
C	-0.68265777314351	4.44569526129812	11.81409751286533
C	1.90681916719909	4.86894621683801	12.99130037477694
C	0.06117995412566	3.38590449706165	12.24553148350408
C	-1.87845796593627	3.83603879076048	14.53332502309128
C	0.12712831653659	1.99755004307178	11.69660959763706
H	1.17108520878287	1.70600397560667	11.45094755947492
H	-0.48706697019775	1.87841013123732	10.78381217661125
H	-0.23784666281623	1.26582613166971	12.45048525017478
C	-1.64312679160609	4.51683024609256	10.67149508876170
H	-2.67031785218254	4.72722134189885	11.04234190177198
H	-1.67781986713453	3.57319907788760	10.09442014836320
H	-1.38355855228282	5.34030095576725	9.97168196090656
C	-3.13230636866029	4.40675061757360	14.23556392141884
H	-3.19815304957995	5.45172824517816	13.89529007684562
C	-4.30065905185004	3.64266094837608	14.37963423628565

H	-5.27776044662409	4.09041414132713	14.14299471277362
C	-1.80357132702430	2.50910958271775	15.00244014605566
H	-0.82890640612973	2.06817854000377	15.26260370553560
C	-4.22380929002654	2.31356691622793	14.82948665558199
H	-5.14151293105952	1.71710224224346	14.94356155258139
C	-2.97551404885385	1.75016839197976	15.14443023288769
H	-2.91310403670457	0.71318157013697	15.50770303407334
C	3.26966427443721	7.68308807639649	15.09666192180226
C	3.62788312076683	8.98362316558261	14.69193280553699
H	3.39675131351672	9.32555945194068	13.67513776990726
C	4.26091698134197	9.83845117589039	15.60454899852273
H	4.53572099247471	10.85678014052374	15.29048799483672
C	4.53785450711268	9.40215899483419	16.91151826435683
H	5.02840963380022	10.07959094387125	17.62684093390708
C	4.17873862466013	8.10328018189409	17.30377504292742
H	4.37239518592888	7.75766345949848	18.32970910481590
C	3.54509089488367	7.23735318926319	16.40205557048380
H	3.25920354486823	6.22617245587682	16.71153650146914
H	1.22988360704651	6.32496008302033	11.44108340522068
H	2.55336492515320	4.43065864630425	12.20476458625431

Anti-endo

48

Coordinates from ORCA-job orca

P -1.31999199591437 4.11647541403706 14.53853773418435

O 3.17285415479797 2.87834806775436 13.11523688368278

C 5.95180483185259 4.87290961461967 10.67292940906934

O 1.74005173954049 7.21664986708055 12.37079471333915

N 2.72229653839951 5.09868251610588 12.53111331918350

C 5.00387486454991 4.75102309794297 11.69932690763379

H 5.26796400337449 4.27152922463821 12.65037368797053

C 3.69808999339449 5.23616376593310 11.49658505224466

C 4.30253449858627 5.96707353620667 9.26264755311235

C 5.60705664005958 5.48245316185758 9.45515503157257

C 0.13861469359603 3.18529531134423 13.79727675062178

H 0.26459736095368 2.12695745738874 14.08319427258098

C 3.34193114542819 5.84208146827486 10.27655308662561

C 2.52545402012254 3.89843677988768 13.25495752250289

C 0.87416131562149 5.55863087713545 13.96039491800123

H 0.95262322859726 6.19529413031619 14.86356186294068

C -0.60380093742508 5.47750652096435 13.45474207426693

H -1.13259627337782 6.44585074751676 13.43828869281515

C 1.79789668209425 6.11249221890705 12.87744848763140

C -0.60747464296972 4.71169358836254 12.12756140698514

C 1.33995562357200 4.10406460243379 14.19322446642656

H 1.66077486111607 3.90591684453182 15.23494540502248

C -0.18577064847501 3.42597726767332 12.31924013102813

Au -3.38004664471455 3.37417343982849 14.05947614291819

C 0.01046626839071 2.33840751955890 11.31361563139194

H 1.05667289915260 1.96575480365609 11.33600321692276

H -0.22003311652624 2.67482129991539 10.28538093072869

H -0.64692351241816 1.47353187510836 11.54748156414335

C	-1.00286395555601	5.39568622347450	10.85900242750226
H	-2.07178978354189	5.69743788655014	10.90104861878601
H	-0.86626038505212	4.74569622647896	9.97435370832775
H	-0.41262463554367	6.32498909536370	10.71186439085596
H	4.02503363398931	6.44529378720590	8.31120523888326
H	6.97111947829975	4.48927013058382	10.83019263496008
H	2.32249109624634	6.21971836451412	10.13026005102832
H	6.35584945700229	5.57949229225984	8.65464338520475
Cl	-5.47700254181627	2.60069473023797	13.52056111129968
C	-0.93250068402218	4.49782291697686	16.28236759856241
C	-1.14331783010255	5.79654651035785	16.79007713217830
H	-1.52336857431684	6.59376816777196	16.13176842578296
C	-0.87498966516965	6.07357453676338	18.13965898305587
H	-1.03926149782507	7.08851347515851	18.53205755420970
C	-0.40191964067541	5.05726229862384	18.98722960486349
H	-0.19228400099014	5.27671404064231	20.04497094235756
C	-0.20297785068597	3.75902218341057	18.48654395414450
H	0.15954143437481	2.95984615890226	19.15059552911147
C	-0.47056476525743	3.47603888638807	17.13835848100334
H	-0.32433688073648	2.45461706935507	16.75259337233432

Anti-exo

48

Coordinates from ORCA-job orca

P	-0.62047549866592	4.66095822458006	14.39650854343313
O	3.42907104063162	4.54349641750347	15.00465048807996
O	1.10822443084368	8.23251005108258	13.37294183880303
N	2.48922366285396	6.64217001251143	14.44994917622615
C	0.76965190181723	3.65190523325358	13.60398109482251
H	1.17346818850496	2.80611392597036	14.18667076541251
C	2.69569651553853	5.23762933822993	14.32428983097883
C	1.07438273624803	5.92111616387701	12.64554400181308
C	-0.44437722631596	5.57847172968482	12.75259501552635
H	-1.11516465887778	6.43760245904196	12.57934221506431
C	1.52639380205778	7.09624594301902	13.50214467445920
C	-0.68437188336643	4.35838925883382	11.85408148835538
C	1.83949920872946	4.70601040718767	13.18252671122674
C	-0.00231893033812	3.27502367738477	12.33336584011612
Au	-2.55444218440870	3.55215394006558	14.69179621540296
C	0.01609581485935	1.87240132723297	11.81939704382072
H	1.05379384950760	1.51530362051457	11.64548370036797
H	-0.55095480634846	1.76650701622763	10.87542873881811
H	-0.43989086510210	1.18826548835340	12.56769000185110
C	-1.60241165463790	4.44702226349081	10.67883824544324
H	-2.63621825082080	4.67283708991179	11.01968733513376
H	-1.63494868899370	3.50282942450862	10.10328407839663
H	-1.30379265433445	5.26580458649408	9.98959189337361
C	3.02813480067701	7.45043925940879	15.50998233750881
C	2.69446642649047	8.82191466859009	15.60623700520387
H	2.05693829004373	9.27881540687136	14.84480403017622
C	3.16086923428454	9.58198660581956	16.68727562652611
H	2.88204265570025	10.64516387029298	16.74696171132647

C	3.96662121723460	9.00653697824852	17.68120868294463
H	4.32540265947124	9.60946174032638	18.52909229473649
C	4.32039441780158	7.65372228256880	17.56786925220429
H	4.96340340174053	7.18391561079125	18.32778617333529
C	3.86427563874166	6.87538187943983	16.49549549291299
H	4.13890469892410	5.81975935697703	16.42431329176839
H	1.30770499546686	6.16813472882378	11.59015756842654
H	2.52227684671851	4.24267072684007	12.44187984203660
Cl	-4.51429296467310	2.37435486313982	14.94388394994033
C	-0.04109449878696	5.65405246065347	15.81399189531215
C	-0.46674740653643	6.99157380169821	15.95826530212092
H	-1.07302901314865	7.47015592888267	15.17476738691892
C	-0.09539705598504	7.72205484143387	17.09695210322125
H	-0.41096874861423	8.77107115215047	17.19593993989761
C	0.68032397404048	7.12137576955669	18.10152238051007
H	0.98012917748458	7.70159570967426	18.98616112611063
C	1.09116897286808	5.78546181431921	17.96470303536241
H	1.70653063233556	5.31454369980338	18.74540968019574
C	0.72734389397221	5.04618247157781	16.82943012157408
H	1.05507390436651	4.00071677315150	16.72783083280286

References

[1] J. Koziskova, F. Hahn, J. Richter, J. Kožíšek, *Acta Chimica Slovaca* **2016**, *9*, 136.

[2] O. V. Dolomanov, L. J. Bourhis, R. J. Gildea, J. A. K. Howard, H. Puschmann, *J. Appl. Crystallogr.* **2009**, *42*, 339.

[3] G. M. Sheldrick, *Acta Cryst. A* **2015**, *71*, 3.

[4] G. M. Sheldrick, *Acta Cryst. A* **2008**, *64*, 112.

[5] G. M. Sheldrick, *Acta Cryst. C* **2015**, *71*, 3.
